# Experimental Treatment of Ebola Virus Disease with TKM-130803: A Single-Arm Phase 2 Clinical Trial

**DOI:** 10.1371/journal.pmed.1001997

**Published:** 2016-04-19

**Authors:** Jake Dunning, Foday Sahr, Amanda Rojek, Fiona Gannon, Gail Carson, Baimba Idriss, Thomas Massaquoi, Regina Gandi, Sebatu Joseph, Hassan K. Osman, Timothy J. G. Brooks, Andrew J. H. Simpson, Ian Goodfellow, Lucy Thorne, Armando Arias, Laura Merson, Lyndsey Castle, Rebecca Howell-Jones, Raul Pardinaz-Solis, Benjamin Hope-Gill, Mauricio Ferri, Jennifer Grove, Mark Kowalski, Kasia Stepniewska, Trudie Lang, John Whitehead, Piero Olliaro, Mohammed Samai, Peter W. Horby, for the RAPIDE-TKM trial team

**Affiliations:** 1 Centre for Tropical Medicine and Global Health, University of Oxford, Oxford, United Kingdom; 2 Military 34 Hospital, Republic of Sierra Leone Armed Forces, Freetown, Sierra Leone; 3 College of Medicine and Allied Health Sciences, Freetown, Sierra Leone; 4 GOAL Global, Dun Laoghaire, Ireland; 5 International Severe Acute Respiratory and Emerging Infection Consortium Coordinating Centre, University of Oxford, Oxford, United Kingdom; 6 Rare and Imported Pathogens Laboratory, Public Health England, Porton Down, United Kingdom; 7 Division of Virology, Department of Pathology, University of Cambridge, Cambridge, United Kingdom; 8 Cardiff and Vale University Health Board, Cardiff, Wales, United Kingdom; 9 Tekmira Pharmaceuticals, Burnaby, British Columbia, Canada; 10 Department of Mathematics and Statistics, Lancaster University, Lancaster, United Kingdom; 11 UNICEF–UNDP–World Bank–WHO Special Programme for Research and Training in Tropical Diseases, Geneva, Switzerland; Mahidol-Oxford Tropical Medicine Research Unit, THAILAND

## Abstract

**Background:**

TKM-130803, a small interfering RNA lipid nanoparticle product, has been developed for the treatment of Ebola virus disease (EVD), but its efficacy and safety in humans has not been evaluated.

**Methods and Findings:**

In this single-arm phase 2 trial, adults with laboratory-confirmed EVD received 0.3 mg/kg of TKM-130803 by intravenous infusion once daily for up to 7 d. On days when trial enrolment capacity was reached, patients were enrolled into a concurrent observational cohort. The primary outcome was survival to day 14 after admission, excluding patients who died within 48 h of admission.

After 14 adults with EVD had received TKM-130803, the pre-specified futility boundary was reached, indicating a probability of survival to day 14 of ≤0.55, and enrolment was stopped. Pre-treatment geometric mean Ebola virus load in the 14 TKM-130803 recipients was 2.24 × 10^9^ RNA copies/ml plasma (95% CI 7.52 × 10^8^, 6.66 × 10^9^). Two of the TKM-130803 recipients died within 48 h of admission and were therefore excluded from the primary outcome analysis. Of the remaining 12 TKM-130803 recipients, nine died and three survived. The probability that a TKM-130803 recipient who survived for 48 h will subsequently survive to day 14 was estimated to be 0.27 (95% CI 0.06, 0.58). TKM-130803 infusions were well tolerated, with 56 doses administered and only one possible infusion-related reaction observed. Three patients were enrolled in the observational cohort, of whom two died.

**Conclusions:**

Administration of TKM-130803 at a dose of 0.3 mg/kg/d by intravenous infusion to adult patients with severe EVD was not shown to improve survival when compared to historic controls.

**Trial registration:**

Pan African Clinical Trials Registry PACTR201501000997429

## Abstract 2

In a single-arm trial, Peter Horby and colleagues probe the effectiveness of a small interfering RNA-based drug intended to treat Ebola virus disease.

## Editors' Summary

**Background:**

Ebola virus disease (EVD) is a frequently fatal disease that first appeared in human populations in 1976 in central Africa and that recently caused thousands of deaths in West Africa. Ebola virus is transmitted to people from wild animals and spreads in human populations through contact with the bodily fluids (including blood, saliva, and urine) and organs of infected people and through contact with bedding and other materials contaminated with bodily fluids. The symptoms of EVD, which start 2–21 days after infection, include fever, headache, vomiting, diarrhea, and internal and external bleeding. Infected individuals are not infectious until they develop symptoms, but they remain infectious as long as their bodily fluids contain virus, which can be several weeks. Infectious virus can persist in the semen of male survivors and suspected male-to-female sexual transmission was reported to have occurred five months after resolution of EVD. In West Africa supportive care—given under strict isolation conditions to prevent the spread of the virus—may improve survival, but there is no proven, specific treatment for EVD.

**Why Was This Study Done?:**

Several potential treatments for EVD have looked promising in animal studies, including TKM-130803, a drug that prevents the production of two essential viral proteins. In rhesus monkeys, the active component of TKM-130803 provided 100% protection against Makona Ebola virus, the virus variant responsible for the West African EVD outbreak. Here, the researchers evaluate the effectiveness of TKM-130803 in a single-arm phase 2 clinical trial. During the West African EVD outbreak, experts designed the RAPIDE (Rapid Assessment of Potential Interventions and Drugs for Ebola) clinical trial platform to speed up the development of treatments for EVD. Using this platform, prioritised drugs go straight into a single-arm phase 2 trial in which people with EVD are administered a selected drug to generate early evidence of the drug’s effectiveness or ineffectiveness. For the assessment of the TKM-130803, a modified RAPIDE approach identifies evidence of lack of effectiveness in the phase 2 trial, by assessing whether a “futility” boundary—a pre-specified survival probability threshold at 14 days after admission—is reached.

**What Did the Researchers Do and Find?:**

Adults with laboratory-confirmed EVD volunteered to participate in the trial, which was undertaken in Sierra Leone. Trial participants were given TKM-130803 once daily by intravenous infusion for up to seven days, in addition to supportive care. The primary outcome was survival to 14 days after admission, excluding patients who died within 48 hours of admission. Using historical data on survival rates for 1,820 people with EVD, the researchers designed their trial so that if the survival probability of the trial participants was greater than 0.55 (that is, if the chance of a patient being alive after 14 days was greater than 55%), TKM-130803 would be regarded as promising and worthy of further evaluation; if the survival probability of the participants was less than or equal to 0.55, then the futility boundary would be reached and enrollment stopped. After 14 adults had received TKM-130803, the pre-specified futility boundary was reached, and enrollment was stopped. Two patients who received TKM-130803 died within 48 hours of admission and were excluded from the primary outcome analysis. Of the remaining patients who received TKM-130803, nine died and three survived. The researchers estimated that TKM-130803 recipients who survived for 48 hours after being admitted had a probability of surviving to 14 days of 0.27.

**What Do These Findings Mean?:**

These findings show that TKM-130803 given once daily at the dose used in this trial did not improve survival in patients with EVD compared to historic controls. This result contrasts with the protective effect of TKM-130803 and related formulations in non-human primates “challenged” (infected) with Ebola virus. One reason for this difference may be that, whereas in the animal studies, experimental drugs were given soon after viral challenge, in the phase 2 trial, the drug was given when the patients had advanced disease. That is, the failure of TKM-130803 to achieve a survival probability exceeding 0.55 in this study may be because the experimental drug had an insufficient antiviral effect in the face of the high viral loads and existing organ damage in these patients. In patients with advanced disease, it could be that the target survival rate was set too high to detect a small or even moderate beneficial effect of TKM-130803. Thus, further work is needed to assess whether the lack of effectiveness seen in this trial is generalizable to other patient subgroups (for example, patients with less advanced disease and lower viral loads) in other treatment settings.

**Additional Information:**

This list of resources contains links that can be accessed when viewing the PDF on a device or via the online version of the article at http://dx.doi.org/10.1371/journal.pmed.1001997.

- The World Health Organization (WHO) provides information about EVD, information about potential EVD vaccines and therapies, and regular updates on the West African EVD epidemic; the WHO website also provides information about efforts to control Ebola in the field and personal stories from people who have survived EVD
- The UK National Health Service Choices website provides detailed information on EVD
- The US Centers for Disease Control and prevention also provides information about EVD
- The Ebola treatment center in Sierra Leone where this trial was undertaken is run by the international humanitarian organization GOAL Global, whose website provides detailed information about fighting EVD in Sierra Leone
- More information about this trial is available

## Introduction

An advisory panel convened in August 2014 by the World Health Organization (WHO) concluded that unregistered experimental products that showed promising results in laboratory and animal models should be evaluated in humans with Ebola virus disease (EVD) [1]. The pathophysiology of severe EVD in humans is characterised by vascular leakage, shock, coagulopathy, and multi-organ injury, with severity closely correlated with Ebola virus (EBOV) RNA levels in blood (henceforth referred to as “viral load”) [2–7]. Antiviral agents have therefore been a main focus of drug development for EVD. One of the lead experimental therapies prioritised for evaluation by WHO was TKM-100802 (Tekmira Pharmaceuticals, British Columbia, Canada), a lipid nanoparticle (LNP) formulation of small interfering RNAs (siRNAs) directed against the gene products encoding two viral proteins: L polymerase (Lpol), involved in transcription and replication of *Zaire ebolavirus*, and Viral Protein-35 (VP35), involved in suppression of the host immune response [8].

TKM-100802 has been evaluated in guinea pig, non-human primate (NHP), and human phase 1 clinical trials [9]. The TKM-100802 Investigational New Drug Application (IND) is currently on partial clinical hold by the US Food and Drug Administration, whereby TKM-100802 may be used in individuals with confirmed or suspected EBOV infection but may not be administered to normal healthy individuals. The basis for the clinical hold in normal healthy individuals was concern about cytokine release syndrome or infusion-related reactions. Cytokine release syndrome is a pro-inflammatory reaction that occurs when activated lymphocytes and/or myeloid cells release soluble immune mediators following administration of certain therapeutic agents, especially monoclonal antibodies. Onset can be rapid (within hours of administration) and can be life-threatening [10]. TKM-100802 has been administered to five patients with EVD medically evacuated to the US and Europe, and to one individual as post-exposure prophylaxis (personal communication, Mark Kowalski, Tekmira Pharmaceuticals). Since the product was administered on a compassionate basis to these individuals and because the patients simultaneously received other experimental products, it has not been possible to assess the efficacy or safety of TKM-100802 in the treatment of EVD [11,12].

TKM-130803 is a new formulation of TKM-100802 in which the siRNA component has been adapted by two nucleotide substitutions in the VP35 siRNA and a single nucleotide substitution in the L-polymerase siRNA to ensure specificity to the West African Makona variant of *Zaire ebolavirus* causing the 2014–2015 West African outbreak. The siRNA drug component of TKM-130803 is termed siEbola-3 and is formulated with lipid excipients (LNP1 composition) to form LNPs. In rhesus monkeys infected with a lethal challenge of Makona variant EBOV, administration of siEbola-3 formulated with a different LNP (LNP2) and dosed at 0.5 mg/kg/d for 7 d resulted in 100% (3/3) survival when commenced 72 h post-inoculation, a point in the disease course where viral RNA levels up to 10^6^ RNA copies/ml can be detectable by blood sampling [13].

The Rapid Assessment of Potential Interventions and Drugs for Ebola (RAPIDE) clinical trial platform was developed in 2014 to assess potential treatments for EVD. The platform allows a multi-stage approach, and the concept is described elsewhere [14]. This report describes the RAPIDE-TKM trial, an open-label, non-randomised, single-arm trial to generate early evidence of the effectiveness of TKM-130803.

## Methods

### Ethics Statement

The trial was approved by the Sierra Leone Ethics and Scientific Review Committee and the Oxford Tropical Research Ethics Committee. Approval to conduct the trial and import the trial drug was granted by the Pharmacy Board of Sierra Leone. The Committee for Medicinal Products for Human Use of the European Medicines Agency was asked for an opinion on the use of TKM-130803 in humans with EVD and was of the view that conducting a clinical trial of TKM-130803 in the context of the Ebola outbreak was acceptable. The UK Department for International Development and GOAL Global approved for the trial to be conducted at the Port Loko Ebola treatment centre (ETC). An independent data monitoring committee (IDMC) reviewed data on a sequential basis and reviewed any reported adverse events or other safety concerns. The trial was conducted in compliance with the International Conference on Harmonisation guidance on good clinical practice, and the Pharmacy Board of Sierra Leone conducted a good clinical practice compliance inspection during the trial. Written informed consent was obtained for all participants, including those enrolled in the observational cohort.

### Trial Setting

The trial was conducted between 11 March and 15 June 2015 at the Port Loko (Mathaska) ETC in Sierra Leone. The ETC was operated by the international humanitarian organisation GOAL Global. The on-site laboratory was operated by Public Health England.

### Patients

Patients with laboratory-confirmed EVD aged 18 y or older were eligible for enrolment. Exclusion criteria were as follows: underlying disease or condition that could jeopardise the safety of the participant or other individuals, patient determined by the treating physician on admission to be for end-of-life care only, intravenous access not possible, use of any other investigational or non-registered product within 30 d prior to trial enrolment or planned use during the trial period, and inability to comply with protocol requirements. There were additional criteria to receive TKM-130803 (those not meeting these criteria could participate in the observational cohort): female patients aged 18–49 y were required to have a negative beta-HCG pregnancy test prior to enrolment, women who were lactating had to agree to stop breastfeeding, sexually active participants had to agree to use condoms for at least 3 mo following discharge. Pregnant and breastfeeding women could not be included initially due to an absence of fertility and reproductive toxicity study data for TKM-130803 or its predecessor compound, TKM-100802. Children could not be included initially because TKM-130803 had never been used in children, and the bio-distribution and pharmacokinetics are not known in this population. It was planned that, after the first 15 patients had received TKM-130803, the possibility of enrolling these patient groups would be considered by the IDMC following a review of all available safety data. A patient could be enrolled anytime within 48 h of first arriving at the ETC with a confirmed diagnosis or within 48 h of being informed of a *Zaire ebolavirus*–positive PCR result if this occurred whilst admitted in the centre.

### Study Drug

The study drug (TKM-130803) was a liquid (non-lyophilised) formulation of siEbola-3 with LNP1. TKM-130803 was administered at a dose of 0.3 mg/kg/d for 7 d by intravenous infusion at a rate of 1.25 ml/min over 2 h, for a total infused volume of 150 ml. The dose of 0.3 mg/kg/d for 7 d was selected based on the safety and pharmacokinetics of TKM-100802 in a single ascending dose study in healthy adult volunteers, in which the maximum tolerated dose was determined to be 0.3 mg/kg. In NHPs infected with EBOV Kikwit in a fatal infection model, 100% survival was observed following administration of TKM-100802 at 0.5 mg/kg/d and 66% survival was observed following administration of TKM-100802 at 0.2 mg/kg/d (personal communication, Mark Kowlaski, Tekmira Pharmaceuticals). Thus, the dose of TKM-130803 selected for this trial was determined to be appropriate in terms of balancing safety and potential clinical benefit. In the event of suspected drug-related toxicity or change in the patient’s clinical condition, the dose of TKM-130803 could be reduced to a minimum of 0.24 mg/kg/d.

### Non-trial Treatment

ETC clinicians provided standard supportive care in accordance with established GOAL Global treatment guidelines for EVD. This included routine malaria testing and treatment, empirical antibiotics, antihelminthics, antiemetics, anti-diarrhoeal therapy, pain relief, oral and intravenous fluid therapy, and electrolyte supplementation, as appropriate.

### Primary Outcome

The primary outcome was survival in patients who received TKM-130803 at a dose of 0.3 mg/kg/d in addition to standard care, assessed 14 d after admission, excluding patients who died within 48 h of admission. Survival at day 14 was chosen since most EVD deaths occur within 14 d of admission to an ETC, and this early time point allowed a rapid assessment of treatment effect.

### Trial Design

The trial was an open-label, non-randomised, single-arm trial with a concurrent observational study.

### Design Rationale

The general approach of the RAPIDE platform in evaluating potential treatments of EVD was to begin with a single-arm phase 2 trial to generate early evidence of effectiveness or ineffectiveness. If the initial phase 2 trial provided evidence of effectiveness (as assessed against a predetermined survival probability threshold at 14 d after enrolment), the phase 2 trial result would require confirmation in follow-up studies [14]. The additional ethical and practical considerations that influenced the decision to perform an initial single-arm trial have been discussed elsewhere [15].

The evaluation of TKM-130803 was, however, constrained by the fact that only 100 courses of the drug were available and that the incidence of new cases was falling dramatically in early 2015. This led to a modification of the phase 2 component of the RAPIDE approach and a realisation that, should testing be indicated beyond phase 2, it might have to take place during a subsequent outbreak of EVD. Therefore, for the TKM-130803 trial, the single-arm phase 2 design did not allow early stopping in the case of evidence of effectiveness, because enrolling the full 100 patients would maximise the precision of the final estimate of effectiveness. A futility design was used to allow early stopping in the event of evidence of futility or harm [16]. The trial would recruit up to 100 patients but would stop if the number of successes observed fell below a pre-specified threshold. To avoid early stopping due to enrolment of patients with very severe, late-stage EVD—who may not be expected to survive even with an effective antiviral therapy—the stopping rule was to be calculated after exclusion of enrolled patients who died within 48 h of admission to the ETC.

Because of the potential risk of infusion reactions, TKM-130803 was infused over a minimum 2-h period, during which clinical monitoring for infusion reactions took place. The intensity of required clinical monitoring and the challenges of care delivery within the ETC meant that the number of participants who could safely receive TKM-130803 infusions concurrently was limited. Therefore, the maximum number of patients receiving TKM-130803 on a single day was capped. Each day the clinical trial lead physician decided the maximum number of beds available for patients to receive TKM-130803 (“TKM beds”). If on any given day the number of patients eligible for and consenting to inclusion in the trial exceeded the number of available TKM beds, patients were randomly allocated to receive either TKM-130803 with standard care (as part of the TKM-130803 cohort) or standard care alone (as part of the observational cohort). Consent for inclusion in the trial included consent to randomisation and inclusion in the observational cohort if TKM bed capacity was reached. Random allocation was conducted using R (R Project for Statistical Computing).

### Statistical Analysis Plan

For the purposes of determining futility, the effectiveness of TKM-130803 was judged in terms of the probability that a patient allocated to receive TKM-130803 would survive to day 14 after admission, after excluding patients who died within 48 h of admission to the ETC. If the survival probability (*p*) was >0.55, then TKM-130803 would be regarded as “promising”; otherwise, it would be regarded as “not promising”. The power of the study (1 − beta) to detect that TKM-130803 was promising was 0.827 if the true success rate *p* was equal to 0.70, and 0.973 if *p* was equal to 0.75. If the trial ever reached a point at which significant evidence (at the one-sided 2.5% level) that *p* was greater than 0.55 could not be found, then continuation of the trial would be considered futile, and it would be stopped. The choice of 0.55 as the target for *p* was made following an analysis of individual-level data on 1,820 adult patients with PCR-confirmed EBOV infection from the 2014–2015 outbreak (personal communication, Annick Antierens, Médecins Sans Frontières). EBOV PCR cycle threshold (Ct) values and viral load data were not available for this historic cohort. The properties of the design were calculated exactly, based on the independent Bernoulli distributions of each patient outcome.

The data management centre was informed every time a patient was enrolled and, after 14 d, whether that patient did or did not survive. Every time that a day 14 report was received, the number of patients who had survived to day 14 was plotted against the number who had been entered into the trial, and the plotted point was compared with the futility boundary. Enrolment into the trial would be stopped if the futility boundary was reached. When the trial was completed, a point estimate and a 95% confidence interval for *p* was computed using the method of Jovic and Whitehead [17]. Provided that all 100 patients received TKM-130803 without the futility boundary being reached, the formal conclusion of the trial would be that TKM-130803 was promising, and this would happen with probability ≤ 0.025 if in fact the success rate *p* was ≤0.55.

### Safety Assessments

Safety assessments of patients included monitoring of vital signs (pulse rate, blood pressure, respiratory rate, temperature, and level of consciousness), symptoms, and the occurrence of serious adverse reactions (SARs) and suspected unexpected serious adverse reactions. Vital signs were assessed at the following time points: pre-infusion, during the infusion (preferably between 30 and 90 min after the start of infusion), at the end of infusion, and at approximately 1, 2, 4, and 8 h after the end of infusion, as well as at additional time points if indicated by the patient’s clinical condition. Trial staff observed patients directly throughout the entire infusion period. Trial assessments and observations were in addition to routine clinical assessments performed by the ETC’s clinicians.

To assess the feasibility and safety of dosing with TKM-130803 in an ETC in Sierra Leone, data on the first four enrolled patients were assessed by the IDMC prior to opening enrolment to additional patients. These first four patients (termed the safety cohort) were recruited sequentially, with each patient receiving at least three doses (or dying) before dosing of the next patient started. It was planned that the safety cohort could be expanded following advice from the IDMC, although this was not required.

### Laboratory Methods

#### Diagnostic Ebola virus PCR RNA extraction

In a flexible film isolator, viral RNA was extracted from EDTA–whole blood either (1) from 80 µl of plasma using the EZ1 Virus Mini Kit v2.0 (Qiagen) in conjunction with the EZ1 platform (Qiagen) or (2) manually from 50 µl of plasma using the QIAamp Viral RNA Kit (Qiagen). Intact MS2 phage was included in all extractions as an exogenous internal control for the downstream reverse transcription PCR (RT-PCR) step. Extracts were resuspended in 60 µl of AVE buffer (Qiagen), and diagnostic EBOV RT-PCR was carried out immediately. Residual extracts were frozen pending reverse transcription quantitative PCR (RT-qPCR).

#### Diagnostic RT-PCR analysis

Qualitative RT-PCR for detection of *Zaire ebolavirus* was performed using duplex RT-PCR with primers/probes directed against the Zaire EBOV nucleoprotein (FAM channel) and the MS2 genome (Alx532 channel; in-house assay) using TaqMan Fast Virus 1-Step Master Mix (Applied Biosystems) on a SmartCycler II platform [18]. Assays were conducted with the following cycling conditions: 50°C for 5 min (one cycle), 95°C for 20 s (one cycle), 95°C for 3 s and 60°C for 30 s (45 cycles). A single fluorescence read was taken at the end of each 60°C step. Samples with Ct > 40 and a positive internal control were interpreted as EBOV negative. Samples with Ct ≤ 40, with or without a positive internal control, were interpreted as EBOV positive.

#### Viral load determination by RT-qPCR

Viral loads were estimated by determining the level of nucleoprotein-containing RNA per millilitre of plasma based on a previously described assay [18]. RNA extracts were diluted 1:5 in 100 ng/µl yeast RNA dilution buffer (Ambion) for genome quantification in triplicate by one-step RT-qPCR. Briefly, diluted RNA extracts were mixed with TaqMan Fast Virus 1-Step 4X Master Mix (Applied Biosystems), primers NP1-F (TCTGACATGGATTACCACAAGATC) and NP1-R (GGATGACTCTTTGCCGAACAATC), and the NP1 probe (6-FAM-AGGTCTGTCCGTTCAA-MGB). One-step RT-qPCR was performed on a LightCycler 96 (Roche) beginning with reverse transcription at 50°C for 5 min, followed by heat denaturation at 95°C for 20 s and 50 cycles of 95°C for 3 s and 60°C for 30 s. The genome copy number was interpolated from a standard curve generated by serial dilution of a plasmid containing the NP1 amplicon, and was calculated per millilitre of plasma. The limit of detection for diluted RNA extracts was 1 × 10^3^ copies. For samples falling below the limit of detection, genome quantification was repeated using undiluted RNA.

#### Biochemistry and haematology

Biochemistry and haematology testing was introduced for all patients at the ETC only midway through the trial, due to constraints beyond the control of the study team. Haematology and blood chemistry assays were performed using an ABX Micros ES 60 haematology analyser (Horiba) and the Fuji DRI-CHEM NX500i platform (Fujifilm), respectively, according to the manufacturers’ instructions. Coagulation tests (activated partial thromboplastin time [APTT and APTT citrate] and prothrombin time [PT and PT citrate]) were carried out using a Hemochron Signature Elite Whole Blood Microcoagulation System (International Technidyne), according to the manufacturer’s instructions. All patients were tested for malaria using the SD Bioline Malaria Ag P.f test (Standard Diagnostics).

## Results

### Trial Patients

**Fig 1:**
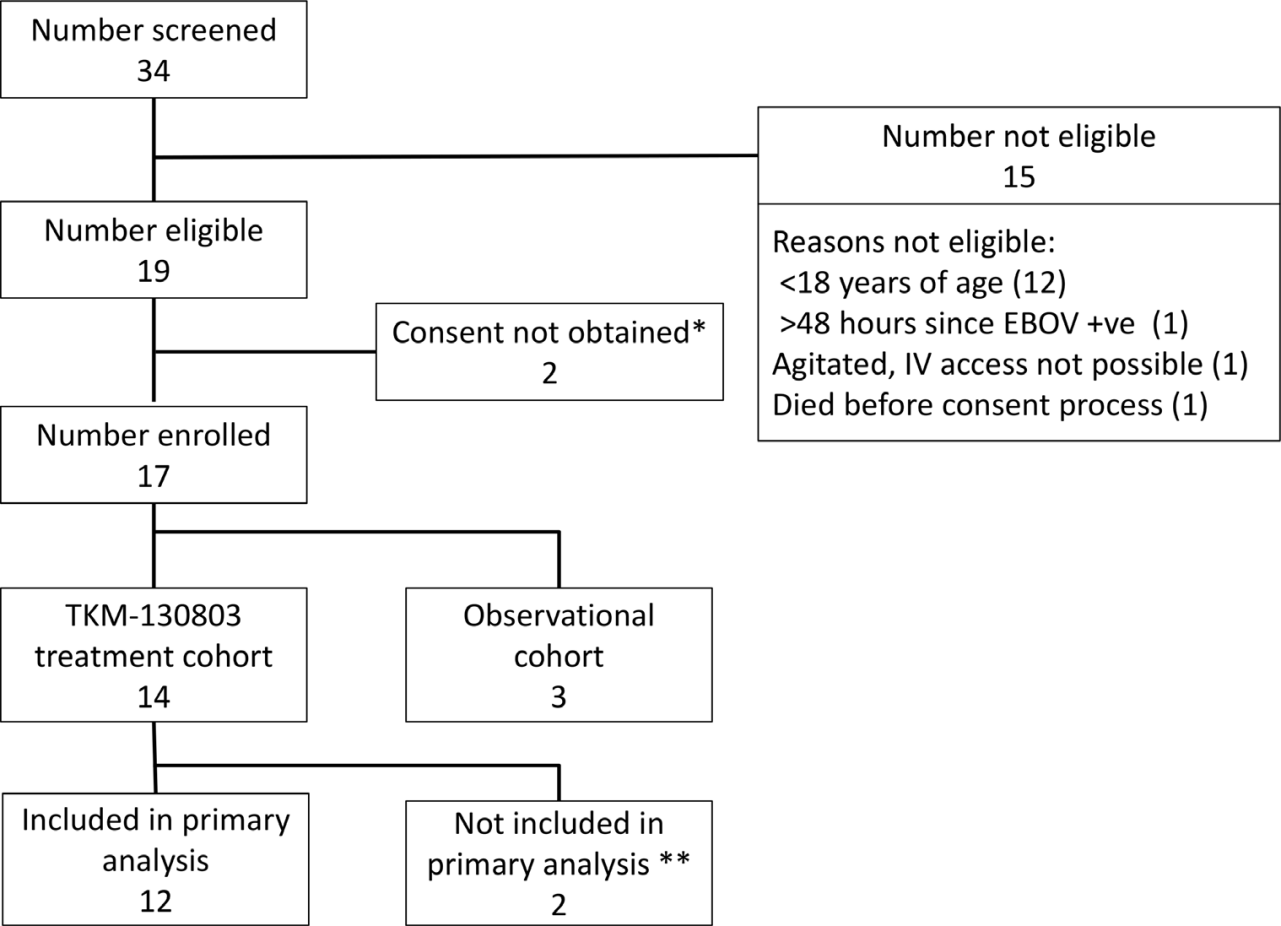
Patient screening and enrolment. *One patient did not give consent. One patient was not competent to give consent, and a suitable proxy to provide consent could not be identified within inclusion time limits. **Two patients who died within 48 h of admission were excluded from the primary outcome analysis, as specified in the protocol.

Thirty-four patients with confirmed EVD were admitted to the ETC during the 3-mo recruitment period, and 17 patients were enrolled (Fig 1). Fourteen patients were enrolled into the TKM-130803 cohort, and three were enrolled into the observational cohort. The three observational cohort patients were recruited during the initial safety cohort phase. TKM bed capacity was never exceeded following the initial safety cohort phase, and randomisation of patients for operational reasons was therefore not required after that initial phase. None of the enrolled patients shared a known close genetic relationship.

### Baseline Characteristics

The median age of the 17 patients was 36 y (range 20 to 85 y), and the median number of days from illness onset to admission was 2 d (range 0–4 d) for the TKM-130803 recipients and 5 d (range 4–6 d) for the observational patients (Table 1). Baseline values for vital signs and blood results were determined from the first set of post-enrolment collections in observational cohort patients and from the collections taken immediately before the first infusion in the TKM-130803 cohort. At baseline, the geometric mean EBOV load in all 17 participants was 1.56 × 10^9^ RNA copies/ml plasma (95% CI 5.75 × 10^8^, 4.23 × 10^9^). The pre-treatment geometric mean EBOV load in the 14 TKM-130803 recipients was 2.24 × 10^9^ RNA copies/ml plasma (95% CI 7.52 × 10^8^, 6.66 × 10^9^). The median time from admission to the ETC to receiving study drug was 23 h. Diarrhoea or vomiting was reported or observed at baseline in 11/17 patients, and 5/17 patients had evidence of bleeding complications on admission. Three patients had a positive malaria rapid test on admission and were treated with artesunate/amodiaquine. In six patients in whom renal function could be determined on admission, three had evidence of renal failure (serum creatinine ≥ 3-fold the upper limit of normal). All five patients in whom hepatic function could be determined on admission had results consistent with liver injury (aspartate aminotransferase ≥ 3-fold the upper limit of normal). Three of four patients in whom coagulation studies were performed on admission had abnormal coagulation profiles.

**Table 1:** Baseline demographic and clinical characteristics of trial population.

Characteristic	TKM-130803 Cohort															Observation Cohort			
	Patient ID														Summary	Patient ID			Summary
	001	004	005	007	020	021	022	025	027^†^	028	030^†^	031	032	034		002	003	006	
Age group (years)	30–39	20–29	30–39	20–29	30–39	20–29	≥70	30–39	30–39	30–39	60–69	50–59	40–49	40–49	35.5 (20–85)	40–49	30–39	40–49	40 (35–40)
Sex: male	Y	Y	Y	N	N	Y	Y	N	N	N	Y	Y	Y	Y	9 (64%)	N	Y	N	1 (33%)
Days since onset	4	2	4	3	1	1	2	2	1	1	3	0	NK	1	2 (0–4)	NK	4	6	5 (4–6)
Hours to TKM-130803/observation	20.5	65.4	41.2	43.2	21.2	23.5	38.4	21.9	23.3	22.8	18.5	18.5	30.7	16.2	23.05 (16.2–65.4)	46.2	21.7	28.5	28.5 (21.7–46.2)
Temperature (°C)	37.4	36.6	36.6	36.9	36.9	37.1	37.5	38.3	40.8	37.6	38.2	39.0	38.9	37.2	37.45 (36.6–40.8)	38.8	38.4	37.1	38.4 (37.1–38.8)
Weight (kg)	57	54	41	49	57	50	54	70	88	48	45	63	54	59	54 (41–88)	73	51	50	51 (50–73)
Heart rate (/min)	66	70	94	60	70	59	76	90	120	NK	120	84	77	83	77 (59–120)	80	102	72	80 (72–102)
Respiratory rate (/min)	16	38	22	20	18	20	24	18	NK	NK	40	22	NK	NK	21 (16–40)	32	27	22	27 (22–32)
Mean arterial pressure (mm Hg)	98.5	86	115	65	87	87	153	89	78.5	NK	70.5	73	100	93	87 (65–153)	106	119.5	80	106 (80–119.5)
Fever	Y	Y	Y	Y	Y	N	Y	Y	N	Y	Y	N	Y	Y	11 (79%)	Y	Y	Y	3 (100%)
Headache	Y	Y	Y	N	Y	Y	N	N	Y	N	Y	N	Y	N	8 (57%)	Y	N	N	1 (33%)
Fatigue/general weakness	Y	N	Y	Y	Y	N	Y	Y	Y	Y	Y	N	Y	N	10 (71%)	Y	Y	Y	3 (100%)
Joint or muscle pain/aches	Y	Y	Y	N	Y	N	Y	Y	Y	N	NK	N	N	N	7 (54%)	Y	Y	N	2 (67%)
Hiccoughs	N	N	Y	N	N	N	N	Y	Y	N	N	N	N	Y	4 (29%)	Y	N	N	1 (33%)
Loss of appetite/anorexia	Y	Y	Y	Y	N	Y	N	Y	Y	Y	Y	N	Y	Y	11 (79%)	N	N	Y	1 (33%)
Nausea	Y	N	Y	N	Y	N	N	Y	N	N	Y	N	Y	Y	7 (50%)	N	N	N	0 (0%)
Vomiting	Y	N	Y	N	Y	N	N	Y	N	N	Y	N	N	Y	6 (43%)	N	N	N	0 (0%)
Difficulty swallowing	N	N	N	N	N	N	N	Y	N	N	NK	N	N	N	1 (8%)	N	N	N	0 (0%)
Diarrhoea	Y	Y	Y	N	Y	N	N	Y	Y	Y	Y	N	Y	N	9 (64%)	N	Y	N	1 (33%)
Breathing difficulty	N	N	N	N	N	N	N	N	Y	N	Y	N	N	N	2 (14%)	Y	Y	N	2 (67%)
Cough	Y	N	N	N	NK	N	N	NK	Y	NK	Y	NK	NK	NK	3 (38%)	N	NK	N	0 (0%)
Chest pain	N	N	N	N	NK	N	N	Y	NK	NK	N	NK	NK	Y	2 (22%)	N	N	N	0 (0%)
Abdominal pain	N	N	N	N	Y	N	N	Y	N	N	Y	N	N	Y	4 (29%)	N	N	N	0 (0%)
Bleeding	N	Y	N	N	N	N	N	Y	N	N	Y	Y	N	N	4 (29%)	Y	N	N	1 (33%)
Died	N	Y	Y	N	Y	Y	Y	Y	Y	N	Y	Y	Y	Y	11 (79%)	Y	Y	N	2 (67%)

Summary data given as median (range) or number positive (percent).

^†^Excluded from final analysis since patient died within 48 h of admission.

N, no; NK, not known; Y, yes.

### Study Drug Received and Outcomes

**Fig 2:**
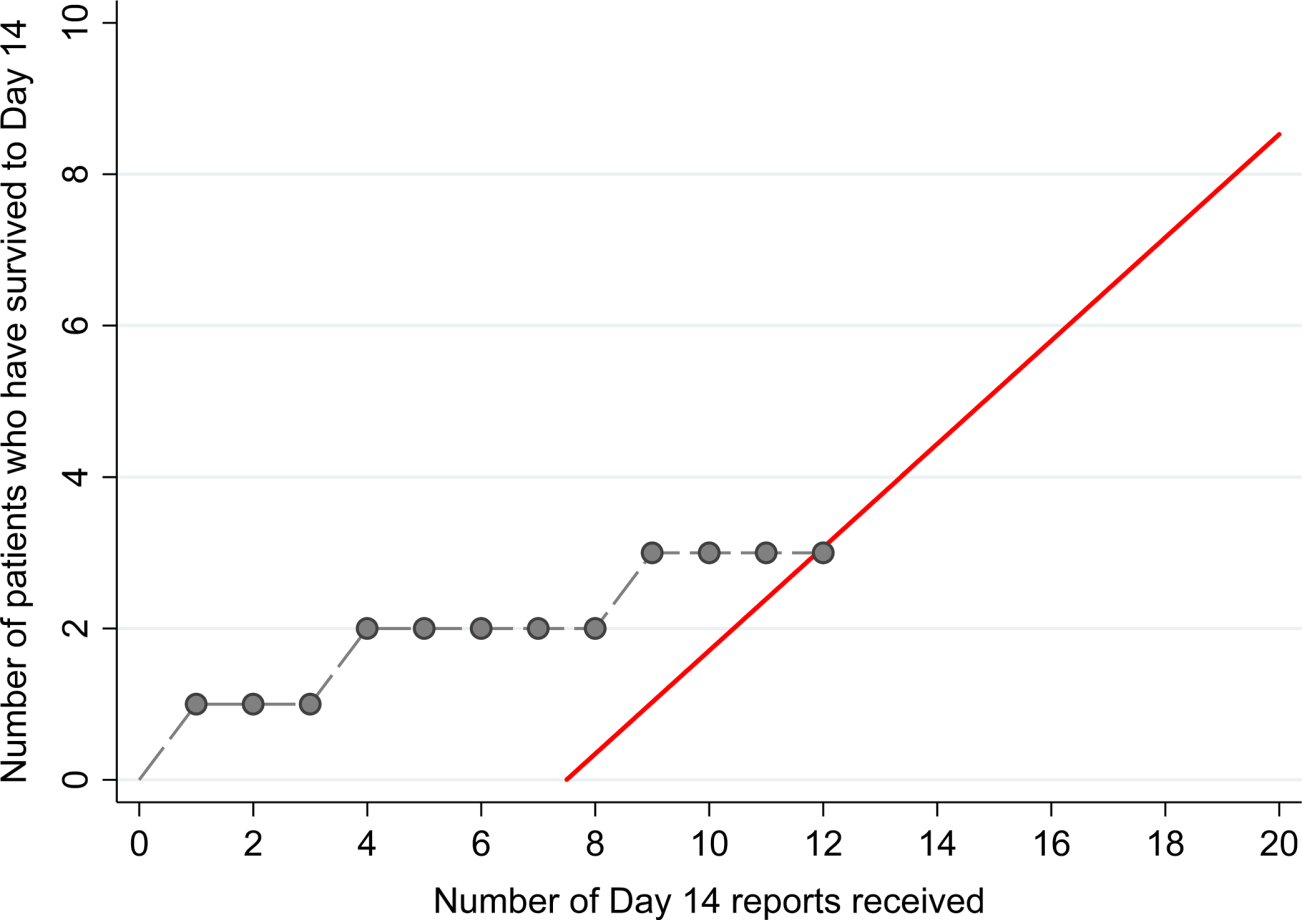
Survival plot with futility boundary for TKM-130803 recipients. The red line denotes the futility boundary. The points and dashed line denote the number of survivors at day 14 plotted against the number of day 14 reports.

**Fig 3:**
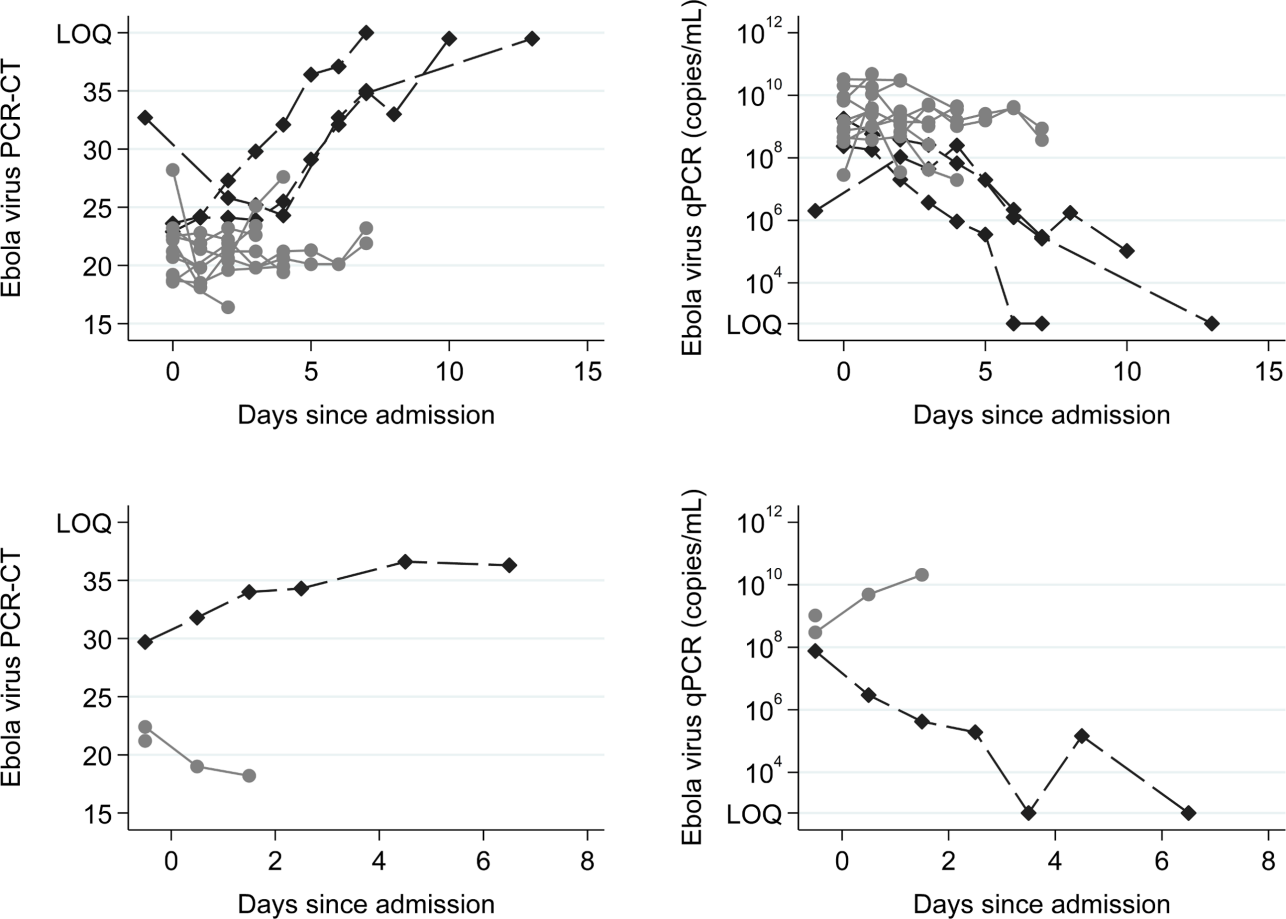
Ebola virus RT-PCR cycle threshold values and RNA copies/ml over time. Top row: TKM-130803 recipients. Bottom row: Observational patients. RT-PCR Ct upper limit of quantitation (LOQ) = 40. RT-qPCR lower limit of quantitation = 1,000 genome copies. The Ebola virus RT-qPCR quantification is expressed as the number of genome copies/millilitre of plasma. Black diamonds denote results for survivors. Grey circles denote results for non-survivors.

The 14 patients enrolled into the TKM-130803 cohort received between one and seven infusions of TKM-130803 (Table 2). Of these 14 patients, three survived to day 14 and were discharged from the ETC, and 11 died. Two patients died within 48 h of admission and were excluded from the primary outcome analysis (and the ongoing futility plot). Two patients died on 15 June 2015, at which point enrolment to the trial was stopped since the futility boundary had been reached, with only three of the twelve patients eligible for inclusion in the primary outcome analysis surviving to day 14 (Fig 2). All deaths were considered to be consistent with severe EVD. In participants who died, viral loads were high at admission and remained high over time (Fig 3); correspondingly, EBOV PCR Ct values were low at admission and remained low over time (Ct values are inversely proportional to viral load). Serial viral load and Ct data were available for two patients in the observational cohort; viral load steadily decreased in the survivor, whereas viral load increased in the patient who died. The final point estimate of the probability that a patient receiving TKM-130803 who survives for 48 h will subsequently survive to day 14 was 0.27 (95% CI 0.06, 0.58). Two of the three patients in the observational cohort died.

**Table 2:** Timelines, TKM-130803 doses received, and outcomes.

Patient ID	Cohort	Day of Onset	DOA	DOA +1	DOA+2	DOA +3	DOA+4	DOA +5	DOA+6	DOA +7	DOA +8	Outcome
203-001	TKM	-4	EVD+	Dose1*	Dose2	Dose3*	Dose4	Dose5	Dose6	Dose7		Alive & Discharged DOA +15
203-002	OBS	Day of onset unknown	EVD+			Died					** **	Died
203-003	OBS	-4	EVD+			Died					** **	Died
203-004	TKM	-2		EVD+		Dose1 Died					** **	Died
203-005	TKM	-4		EVD+	Dose1	Dose2	Dose3 Died				** **	Died
203-006	OBS	-6	EVD+									Alive & Discharged DOA +9
203-007	TKM	-3	EVD+		Dose1	Dose2	Dose3	Dose4	Dose5	Dose6	Dose7	Alive & Discharged DOA +13
203-020	TKM	-1	EVD+	Dose1	Dose2	Dose3	Dose4	Died				Died
203-021	TKM	-1	EVD+	Dose1	Dose2	Dose3	Dose4	Died				Died
203-022	TKM	-2		EVD+	Dose1	Dose2	Died					Died
203-025	TKM	-2	EVD+	Dose1	Dose2 Died							Died
203-027 †	TKM	-1	EVD+	Dose1	Died							Died
203-028	TKM	-1	EVD+	Dose1	Dose2*	Dose3	Dose4	Dose5	Dose6	Dose7		Alive & Discharged DOA +11
203-030 †	TKM	-3	EVD+	Dose1*	Died							Died
203-031	TKM	0		EVD+ Dose 1	Dose2	Dose3	Dose4	Dose5	Dose6	Dose7 Died		Died
203-032	TKM	Day of onset unknown		EVD+ Dose 1	Dose2	Dose3	Dose4	Dose5	Dose6	Dose7 Died		Died
203-034	TKM	-1	EVD+	Dose1	Dose2	Dose3 Died						Died

Legend

OBS = Observational Cohort

TKM = TKM Cohort

†Excluded from final analysis since patient died within 48 hours of admission

Day of Onset= First reported day of onset of symptoms of Ebola virus disease

DOA = Day of admission

EVD+ = Day on which patient received EVD RT-PCR positive result

* Under-dosing event due to loss of study drug volume during additional unanticipated line priming: 203-001 Dose 1=0.28mg/kg; 203-001 Dose 3 =0.28mg/kg. Subject 203-028 Dose 2=0.25mg/kg. Subject 203-030 Dose 1=0.24mg/kg.

### Adverse Events

**Fig 4:**
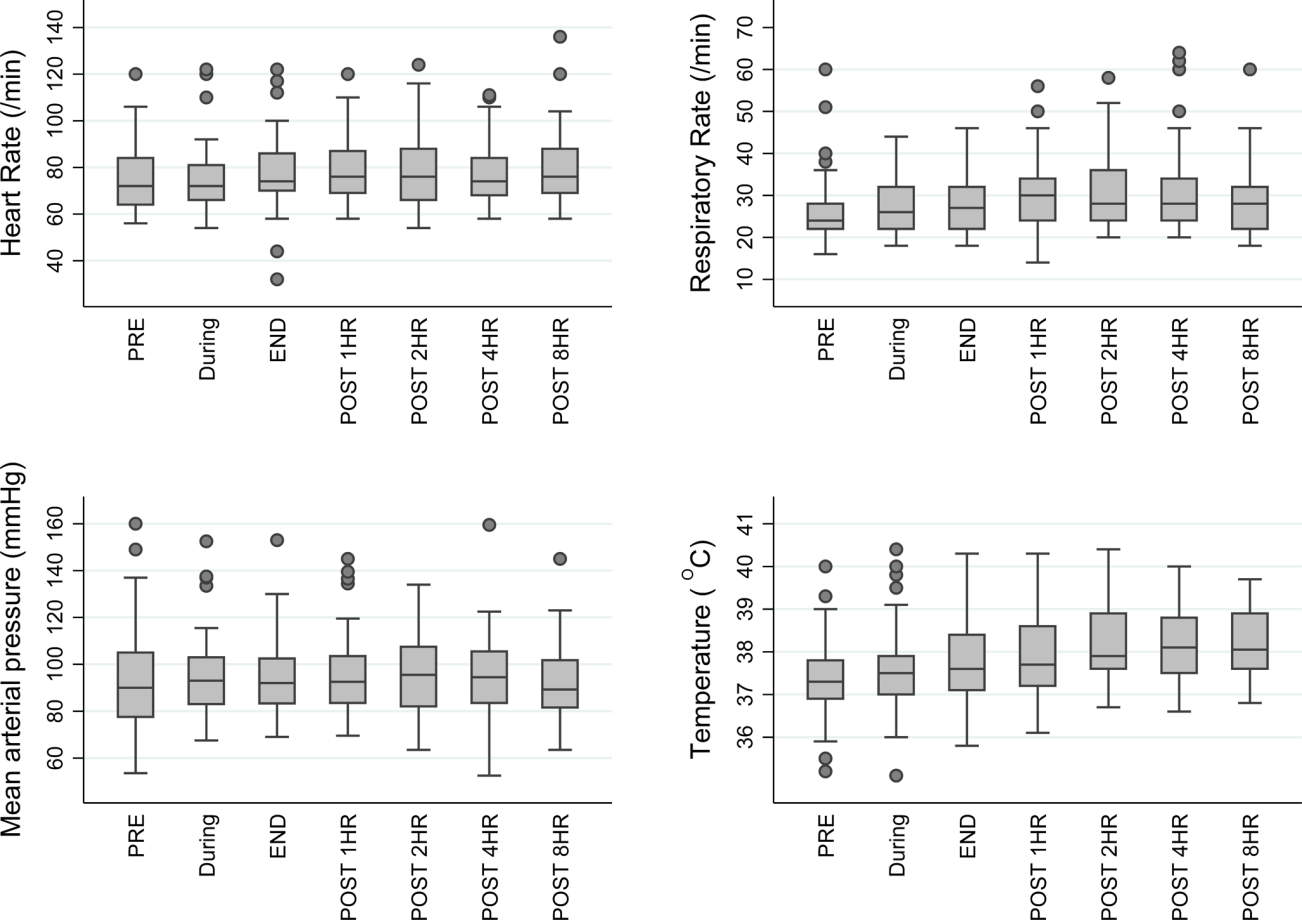
Box and whisker plot of vital signs in TKM-130803 recipients, before, during, and after TKM-130803 infusions. Heart rate, respiratory rate, mean arterial blood pressure, and tympanic temperature in patients administered TKM-130803 at the following time points: immediately prior to TKM-130803 infusion (PRE), during the infusion, immediately at the end of the infusion (END), and at 1, 2, 4, and 8 h after the end of the infusion. The middle line shows the median value, the box shows the interquartile range, and the whiskers spread from the lower to the upper adjacent values. Outside values, that is, observations that are larger/smaller than the upper/lower adjacent values, are shown as circles.

A total of 56 infusions of TKM-130803 were administered. Adverse reactions consistent with acute cytokine release syndrome were not seen during or following any of the infusions, and none of the infusions required termination or slowing of the infusion rate (Fig 4) [10]. As such, the infusions of TKM-130803 were well tolerated. One patient (203–025) was observed to have worsening tachypnoea in the 48 h following the second TKM-130803 infusion, but new onset or worsening of additional symptoms or signs that might indicate infusion-related cytokine release syndrome (tachycardia, flushing, headache, hypotension, chills, nausea, and vomiting) were not observed in this patient. The event was reported to the IDMC as a SAR because of the temporal relationship with the administration of the study drug, but it was also felt the event was compatible with progression of EVD.

## Discussion

Our trial has shown that the administration of TKM-130803 at a dose of 0.3 mg/kg/d in adults with predominantly severe EVD and high viral loads does not improve survival compared to historic survival rates. This result contrasts with the protective efficacy of various formulations of this product observed in NHPs challenged with a lethal dose of EBOV [9,13]. There are a number of potential reasons for the difference between the NHP study results and our results. In the animal challenge studies of siEbola-3, the first infusion was administered between 30 min and 3 d after the lethal viral challenge, with day three roughly corresponding to the first day of clinical illness and viral RNA detection (ranging from just detectable to 10^6^ RNA copies/ml) in serum in the NHP challenge model used (1,000 plaque-forming units administered intramuscularly) [19,20]. Although survival was 100% when the first siEbola-3 infusion was commenced 72 h post-infection, studies of the earlier formulation TKM-100802 found that survival declined with time from infection to first infusion, with 83% survival when the first infusion was started 24–48 h post-infection, declining to 67% survival at 72 h, and 0% survival at 96 h [13,21]. In our trial, the mean number of days from reported illness onset to first infusion was 2 d (range 0–4 d), although there is uncertainty about the reliability of the onset dates reported by participants. This is shorter than the time from illness onset to admission to ETC in several large analyses of patients with EVD in West Africa, which was 5–6 d, but assuming a mean incubation period of 10 d, our patients were still presenting approximately 12 d after exposure [2,4,22–26]. As such, we were administering the study drug to patients later in the infection and disease course than in the NHP models.

All of the 14 patients who received TKM-130803 had ≥1 × 10^8^ RNA copies/ml plasma prior to their first infusion. Although data are currently limited, this level has been associated in other studies with a fatal outcome in >90% of patients [2,3]. Of the 11 TKM-130803 recipients who died, nine had ≥1 × 10^9^ RNA copies/ml prior to their first infusion, and seven presented with symptoms or signs reported by other studies to be associated with a high probability of death (haemorrhagic signs, hiccough, tachypnoea) [2,4,22,23,27]. Therefore, the failure of TKM-130803 to achieve a survival rate exceeding 0.55 may have arisen from an insufficient antiviral effect in the face of high viral loads and existing organ injury in patients presenting with advanced disease. Although data on viral load in our historic patient dataset are not available, it seems likely that the severity of illness of the patients recruited into this trial was greater than the average severity of illness of the historic dataset. As such, in this patient group, a target survival rate of 0.55 is likely to have been too high to allow the study to detect a small or moderate beneficial effect. Nevertheless, the survival probability of 0.27 (95% CI 0.06, 0.58) that we estimated is not encouraging. Whilst a randomised controlled trial could potentially determine whether a survival probability of 0.27 in the experimental treatment arm represents a survival improvement compared to concurrent controls of similar severity, a sample size of around 90 patients in each arm would be required to have 80% power to detect a difference in survival of 0.10 versus 0.27. There were insufficient patients to have conducted such a study, with only one additional patient with confirmed EBOV infection admitted to the treatment centre following closure of the trial. At the location and time our study was conducted, there was also no possibility to determine effectiveness in less severe cases, since less severe cases were not presenting to the ETC. Although the fatal cases in our trial on average presented sooner in their illness than the survivors, this paradox of better survival in patients presenting later has been reported by others, and likely reflects a survival bias, whereby those with the most severe disease do not survive long enough to present late [4].

The optimal dose of TKM-100802 in NHP studies was 0.5 mg/kg/d for 7 d, whereas we administered a dose of 0.3 mg/kg/d based on observed tolerability data in healthy adult volunteers and an assumption that the pharmacokinetics of TKM-100802/TKM-130803 (which share the same LNP composition) in NHPs, healthy human volunteers, and patients with EVD was similar [13]. We did not have sufficient product, patients, or time to conduct dose ranging or dose comparative studies. We do not know whether the dosing regimen used in this trial resulted in adequate drug concentrations or whether higher doses may have resulted in a therapeutic benefit. In addition, the formulation we used differs in the LNP component from that reported in the NHP work of Thi et al. [13]. The lipid excipients used in TKM-130803 are the same as those used in previous healthy human volunteer studies (formulated as TKM-100802), whereas the LNPs used by Thi et al. in NHP studies have not been assessed for safety in healthy human volunteers and were therefore not available for use in this trial.

The trial cannot identify whether the drug is both ineffective and harmful since the futility rule terminated the trial when there was evidence, as pre-specified, that the survival in those receiving TKM-130803 was no better than historic survival rates. Fifty-six separate TKM-130803 infusions were administered, and the patients were monitored closely for adverse events. Contrary to our expectations based on healthy volunteers and repatriated EVD patients given TKM-100802, the infusions were well tolerated, and no obvious cytokine release reactions were observed. Only one SAR, worsening tachypnoea, was reported. This patient had severe EVD, as evidenced by a high viral load, a coagulation disorder, and bloody diarrhoea on admission. Since tachypnoea was present prior to the first TKM-130803 infusion, and because tachypnoea is common in severe EVD (possibly related to metabolic acidosis or pulmonary oedema secondary to vascular leakage), the SAR may not have been causally related to drug administration [5,12,27–31]. Use of TKM-100802 in three medically evacuated patients has been reported, with infusion-related reactions of fever and rigors noted in two, and with the drug being discontinued in one of these patients after six doses due to concerns that the drug may have been contributing to clinical deterioration [11,12]. TKM-100802 and TKM-130803 share the same lipid excipient, and the siRNA components are identical except for minor nucleotide sequence differences. However, TKM-100802 is a lyophilised product, and TKM-130803 is a ready-to-use liquid formulation; therefore, the lyoprotectant excipients present in TKM-100802 are absent from TKM-130803. This might explain the observed differences in the incidence of infusion-related reactions and is consistent with the finding that in human whole-blood cultures TKM-130803 had similar or less capacity than TKM-100802 to cause release of IL-6 and MCP-1 pro-inflammatory cytokines (personal communication, Mark Kowalski, Tekmira Pharmaceuticals). It is also possible that the immune response is dampened in patients with later-stage EVD infection. Overall, TKM-130803 was well tolerated in this study, and the clinical progression of all of the 12 patients who died in the TKM-130803 cohort was consistent with severe EVD with sustained high viral loads.

The RAPIDE studies were set up to rapidly triage potential therapies for EVD, to eliminate agents that are not effective and allow resources to be concentrated on agents with greater promise. The single-arm design with a pre-specified futility boundary and sequential analysis was able to rapidly identify a low probability of survival and cease recruitment in this study, thus minimising harm to patients and risks to healthcare workers and improving the probability that ongoing trials of other interventions could recruit sufficient EVD patients to reach a conclusion.

In summary, administration of TKM-130803 at a dose of 0.3 mg/kg/d to adult patients with EVD and predominantly high levels of viral RNA in blood was well tolerated but did not improve survival compared to historic controls. Further work is needed to assess whether the lack of observed effectiveness is generalisable to other patient subgroups in other treatment settings. Additionally, the potential influence of drug formulation and dose requires further investigation.

## Supporting Information

S1 Text Trial protocol. (PDF)

S2 Text CONSORT checklist. (DOC)

## Abbreviations

Ct: cycle threshold
EBOV: Ebola virus
ETC: Ebola treatment centre
EVD: Ebola virus disease
IDMC: independent data monitoring committee
LNP: lipid nanoparticle
NHP: non-human primate
RT-qPCR: reverse transcription quantitative PCR
RAPIDE: Rapid Assessment of Potential Interventions and Drugs for Ebola
RT-PCR: reverse transcription PCR
SAR: serious adverse reaction
siRNA: small interfering RNA
WHO: World Health Organization
