# Supplementary material for: Experimental Treatment of Ebola Virus Disease with TKM-130803: A Single-Arm Phase 2 Clinical Trial
Source: PLoS Med. 2016 Apr 19;13(4):e1001997. doi: 10.1371/journal.pmed.1001997 (PMC4836798; doi:10.1371/journal.pmed.1001997)
Supplement: S1 Text — (PDF) [file pmed.1001997.s001.pdf]

This supplement contains the following items:

1. Original protocol, final protocol, summary of changes.

Trial Title: Rapid Assessment of Potential Interventions & Drugs for Ebola (RAPIDE) – TKM

Scientific Title: Open-label, single arm trial to investigate the efficacy of TKM-130803 with a concurrent observational study of Ebolavirus Disease in Sierra Leone.

Short title: Evaluation of TKM Treatment for Ebolavirus Disease

Date and Version No: 18 February, 2015 v 2.7

Chief Investigator: **Peter Horby**
Centre for Tropical Medicine and Global Health
Nuffield Department of Medicine Research Building
University of Oxford
Old Road Campus, Roosevelt Drive
OXFORD, OX3 7FZ

Country Principal Investigator: **Foday Sahr** 34 Military Hospital, Ministry of Health and Sanitation Freetown, Sierra Leone

Co-Investigators: **James Russell**, Sierra Leone College of Medicine and Allied Health Sciences

G.F. Deen, Sierra Leone College of Medicine and Allied Health Sciences

Stephan Gunther, Bernard Nocht Institute for Tropical Medicine

Tim Brooks, Rare and Imported Pathogens Laboratory, Public Health England

Trudie Lang Global Health Network, Centre for Tropical Medicine and Global Health, University of Oxford

Piero L Olliaro Newton-Abraham Visiting Professor, University of Oxford.

Jake Dunning (Field Clinical Lead) Centre for Tropical Medicine and Global Health, University of Oxford

John Whitehead Department of Mathematics and Statistics, Lancaster University

Sponsor: University of Oxford

Joint Research Office, Block 60, Churchill Hospital, Oxford, OX4 7LE

Funder: Wellcome Trust

PARCT: PACTR201501000997429

TABLE OF CONTENTS

1. KEY TRIAL CONTACTS 4
2. SYNOPSIS 6
3. ABBREVIATIONS 8
4. INTRODUCTION 9
5. DRUG INFORMATION 10
5.1. Efficacy data against EBOV 10
5.2. Safety and tolerability 10
5.3. Dosing and dosing rationale 11
6. OBJECTIVES AND OUTCOME MEASURES 11
7. TRIAL DESIGN 12
7.1. Trial design 12
7.2. Trial design rationale 12
7.3. Eligibility for TKM 13
7.4. Trial enrolment 13
7.5. Participant inclusion criteria 15
8. TRIAL PROCEDURES 16
8.1. Screening and informed Consent 16
8.2. Procedures for provision of TKM 16
8.3. Laboratory Samples 17
8.4. Procedures for standard care 17
8.5. Treatment Interruption 17
8.6. Discontinuation of Trial Treatment 17
8.7. Withdrawal from the Study 18
9. DATA COLLECTION 18
9.1. Clinical data acquisition 18
9.2. Follow up Assessments (Day 14, Day 30, Months 3, 6, 12) 19
9.3. Laboratory Assessments 20
10. TRIAL DRUGS 21
10.1. Formulation 21
10.2. Storage and Accountability 22
11. SAFETY REPORTING 22
11.1. Definitions for safety reporting 22

11.2. Procedures for safety reporting ... 22
12. STATISTICS ... 23
12.1. Description of Statistical Methods ... 23
12.2. The Number of Participants ... 24
12.3. Criteria for the Termination of the Trial ... 24
12.4. Final Analysis ... 26
12.5. Inclusion in Analysis ... 26
12.6. Procedure for Accounting for Missing, Unused, and Spurious Data. ... 26
12.7. Rationale for statistical design choice ... 26
13. DATA MANAGEMENT ... 27
13.1. Data Capture ... 27
13.2. Access to Data ... 27
14. QUALITY ASSURANCE PROCEDURES ... 27
15. ETHICAL AND REGULATORY CONSIDERATIONS ... 28
15.1. Regulations, Guidelines and Approval ... 28
15.2. Considerations in Informed Consent ... 28
15.3. Participant Confidentiality ... 30
15.4. Sample Confidentiality and Management ... 30
15.5. Risks and Benefits ... 31
15.6. Expenses ... 31
15.7. Contemporaneous Studies ... 32
15.8. Alternatives to Study Participation ... 32
15.9. Community Engagement ... 32
16. FINANCE AND INSURANCE ... 32
16.1. Funding ... 32
16.2. Insurance ... 32
17. PUBLICATION POLICY ... 33
18. REFERENCES ... 34

1. KEY TRIAL CONTACTS

**Chief Investigator**	Associate Professor Peter Horby<br><br>Centre for Tropical Medicine and Global Health<br>Nuffield Department of Medicine Research Building<br>University of Oxford<br>Old Road Campus, Roosevelt Drive<br>OXFORD<br>OX3 7FZ<br><br>Tel: 07990 560237<br>Email: Peter.horby@ndm.ox.ac.uk
**Sponsor**	University of Oxford<br>Joint Research Office<br>Block 60, Churchill Hospital<br>Oxford, OX4 7LE
**Statistician**	Professor John Whitehead<br><br>Department of Mathematics and Statistics<br>Fylde College<br>Lancaster University<br>Lancaster LA1 4YF<br><br>Tel: 01524 389967<br>Email: j.whitehead@lancaster.ac.uk
**Committees**	**Trial Steering Committee Voting Members:**<br><br>**Samuel Kargbo**<br>Ministry of Health and Sanitation, Freetown, Sierra Leone<br><br>**Mohamed Samai,**<br>College of Medicine and Allied Health Sciences, Freetown, Sierra Leone<br><br>**Foday Sahr** 34 Military Hospital, Ministry of Health and Sanitation Freetown, Sierra Leone<br><br>**Stephen Kennedy**<br>Pacific Institute for Research and Evaluation, Monrovia, Liberia<br><br>**Fred Binka** (Independent Member)<br>University of Health and Allied Sciences, Ho, Ghana<br><br>**Peter Horby** (Chief Investigator)<br>Centre for Tropical Medicine and Global Health, Oxford, United Kingdom

International Severe Acute Respiratory and emerging Infections Consortium

John Whitehead (Trial Statistician)
Lancaster University, Lancaster, United Kingdom

Rob Fowler (Independent Member)
Sunnybrook Health Sciences Centre, Toronto, Canada

Nicholas White (Independent Member - Chairperson)
Mahidol-Oxford Research Unit, Bangkok, Thailand

Non-Voting Members

Ana Maria Henao-Restrepo (WHO Representative)
World Health Organization, Geneva, Switzerland

Independent Data Monitoring Committee (IDMC) Members:

David Lalloo (Chair), Professor of Tropical Medicine; Dean of Clinical Sciences and International Public Health, Liverpool School of Tropical Medicine

Clement Adebamowo, Director Office of Strategic Information and Research Institute of Human Virology in Nigeria

Donald Berry, Founder and senior Statistical Scientist, Berry Consulting

Nicolas Opoku, Director Onchocerciasis Chemotherapy Research Centre, Hohoe, Ghana

Peter Smith, Professor of Tropical Epidemiology, London School of Hygiene and Tropical Medicine

Durodami Radcliffe Lisk, Professor Consultant Neurologist, Ministry of Health Coordinator, Epilepsy Association of Sierra Leone, College of Medical and Applied Health (COMAH), Sierra Leone

2. SYNOPSIS

Trial Title	**Rapid Assessment of Potential Interventions & Drugs for Ebola (RAPIDE) – TKM**	
Scientific Title	Open-label, single arm trial to investigate the efficacy of TKM-130803 with a concurrent observational study of Ebolavirus Disease in an outbreak setting in West Africa	
Internal ref. no. (or short title)	Evaluation of TKM-130803 Treatment for Ebolavirus Disease	
Clinical Phase	2	
Trial Design	Open-label, single arm trial, with a concurrent observational study	
Trial Participants	Patients with confirmed Ebolavirus disease attending the participating treatment centre	
Planned Sample Size	Up to 100 adult patients (with possible late inclusion of paediatric patients)	
Treatment duration	1 week	
Follow up duration	12 months follow up	
	Objectives	Outcome Measures
Primary	TKM: To evaluate the impact of TKM treatment on early mortality in EVD<br><br>Observational: To characterize the early mortality of EVD in an ETC	Mortality at Day 14 (in those that survive first 48 hours after admission)<br><br>Mortality at Day 14 (in those that survive first 48 hours after admission)
Secondary	TKM<br>1. To evaluate the impact of TKM-130803 treatment of adults on:<br>a) Time to recovery<br>b) Late mortality<br>c) Viral load<br>d) EVD symptoms<br>e) EVD antibody response<br>f) Long term clinical recovery<br><br>2. i)To assess the safety of TKM treatment of adults<br>ii) To measure the pharmacokinetics (PK) of TKM following repeat dosing<br><br>Observational	TKM<br>a) Time to meeting ETC discharge criteria.<br>b) Mortality at Day 30 and months 3,6,12 after first dose of study treatment<br>c) Viral load<br>d) Presence and duration of symptoms (Days 1-14)<br>e) Convalescent anti-Ebolavirus IgG titer (Day 30)<br>f) Clinical assessment at months 3,6,12.<br><br>2. i) Incidence of SARs, key adverse events (Days 1-14) and monitoring of vital signs (pulse rate, blood pressure, respiratory rate, temperature) pre, during and at 0, 1, 2, 4 and 8 hours post end of infusion<br>ii) PK pre-dose and at the end of infusion on days 1, 3, 5 and 7<br><br>Observational

	To characterise the natural history of EVD in an ETC.<br>a) Time to recovery<br>b) Late mortality<br>c) Viral load<br>d) EVD symptoms<br>e) EVD antibody response<br>f) Long term clinical recovery	a) Time to meeting ETC discharge criteria.<br>b) Mortality at Day 30 and months 3, 6, 12 after admission<br>c) Viral load<br>d) Presence and duration of symptoms (Days 1-14)<br>e) Convalescent anti-Ebolavirus IgG titer (Day 30)<br>f) Clinical assessment at months 3,6,12
Investigational Medicinal Product	TKM-130803 (TKM)	
Formulation, Dose, Route of Administration	TKM is provided as 0.3 mg/kg, administered as a 2-hour intravenous infusion (IV), in a final volume of 150ml, once daily for seven days.<br><br>To assess the feasibility and safety of dosing with TKM-130803 in an ETU, data on the first four enrolled patients (the safety cohort) will be assessed by the IDMC prior to opening enrollment to additional patients. The safety cohort may be expanded following advice from the IDMC.<br><br>In the event of suspected drug-related toxicity or a change in the patient's clinical condition, the dose of TKM-130803 may be reduced to a minimum of 0.24 mg/kg once daily at the discretion of the treating physician. TKM will not be given to children or pregnant women during the initial phase of the study.<br><br>Administration of the drug will adhere to the relevant trial Standard Operating Procedure (SOP) document.	

3. ABBREVIATIONS

AE	Adverse event
CI	Confidence Interval
Coags	Coagulation studies
CRF	Case Report Form
EBOV	Ebolavirus
EDTA	Ethylenediaminetetraacetic acid
ETC	Ebola Treatment Centre
EVD	Ebolavirus Disease
FBC	Full blood count
GCP	Good Clinical Practice
IB	Investigators Brochure
ICF	Informed Consent Form
ICH	International Conference on Harmonisation
IDMC	Independent Data Monitoring Committee
IMP	Investigational Medicinal Product
ITT	Intention to Treat
IV	Intravenous
LFT	Liver function tests
MAD	Multiple Ascending Dose
MSF	Medecins Sans Frontieres
OXTREC	Oxford Tropical Research Ethics Committee
PI	Principal Investigator
PK	Pharmacokinetic
REC	Research Ethics Committee
RNA	Ribonucleic acid
RT-PCR	Reverse transcription polymerase chain reaction
SAD	Single Ascending Dose
SAE	Serious Adverse Event
SAR	Serious Adverse Reaction
SOP	Standard Operating Procedure
SUSAR	Suspected Unexpected Serious Adverse Reactions
TKM	TKM-130803
TSC	Trial Steering Committee
UEC	Urea, Electrolytes, Creatinine
WHO	World Health Organisation

4. INTRODUCTION

The size and scale of the on-going Ebola Virus Disease (EVD) outbreak is unprecedented, and has been declared a Public Health Emergency of International Concern.[1] EVD is among the most virulent infectious agents known: an analysis of data on 3343 confirmed and 667 probable EVD cases collected during the current outbreak in Guinea, Liberia, Nigeria, and Sierra Leone estimates a case fatality rate of 70.8% (95% confidence interval [CI], 69 to 73) among persons with known clinical outcome of infection.[2] A figure that is consistent with estimates of other authors.[3] Figure 1 shows the key time points in the clinical course of EVD in this outbreak.[2]

Figure 1. Estimated mean (median) time course in days of Ebolavirus infection in this outbreak[2]

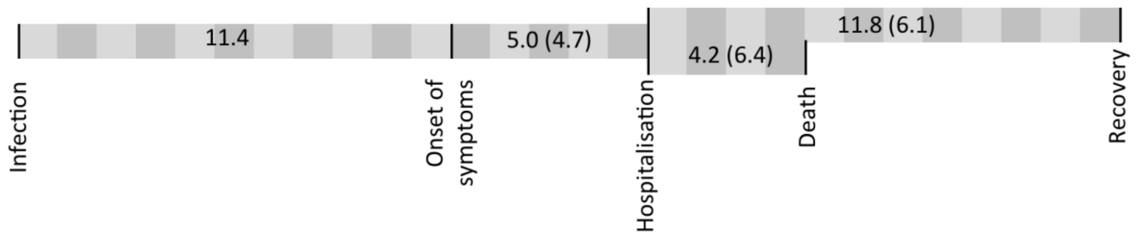

The current Ebolavirus strain causing the outbreak in West Africa is related to the Ebola Zaire strain and genetic analysis suggests the current outbreaks in Guinea, Sierra Leone and Liberia are all related to a single transmission event from a zoonotic source followed by human-to-human transmission.[4] EVD is characterized by a febrile illness dominated by fatigue and gastrointestinal symptoms that can be complicated by shock, haemorrhage and multi-organ failure. However, clinical presentation and severity is variable, with some patients remaining relatively well, able to ambulate and self-feed throughout their illness, whilst others progress rapidly to a fatal outcome.[2,5]

The pathogenesis of EVD is incompletely understood[6,7] but high levels of viral replication and the detection of virus in multiple body tissues is typical of severe disease.[8,9] Coagulopathy, disruption of endothelial function and increased inflammatory responses are also associated with severe EVD.[6,10-12] The association between high levels of viraemia and EVD severity suggests that therapies that target viral replication may benefit patients. Several experimental products that target Ebolavirus replication have shown some efficacy in animal studies.[13-15] However, whilst several therapeutic interventions have shown promise in the laboratory and in animal studies, none have been tested for efficacy and safety in humans with EVD. A World Health Organization (WHO) expert panel recently concluded unanimously that "investigators have a moral duty to evaluate these interventions in the best possible clinical studies that can be conducted under the circumstances of the epidemic."[16]

The aim of this protocol is to assess the effectiveness of the drug TKM-130803 (produced as TKM-130803 by Tekmira Pharmaceuticals) for the treatment of EVD.

The Trial Steering Committee selected TKM as one of the leading drugs suitable for urgent evaluation from a possible list of 10 candidate therapies. The criteria for selection were based upon a Target Product Profile that considered existing clinical and pre-clinical data supporting safety and efficacy, immediate availability and scalability, and an acceptable treatment regimen.

The purpose of this trial is to establish whether TKM is a promising treatment for Ebola Virus Disease.

5. DRUG INFORMATION

TKM-130803 is a drug that has been developed specifically to target EBOV. TKM-130803 is a lipid nanoparticle formulation of 2 small interfering RNAs (siRNAs) directed against Ebolavirus L polymerase and Viral Protein-35 (VP-35). These two proteins are involved in transcription and replication of EBOV. TKM-130803 is a new formulation of drug TKM-100802 (formerly TKM-100201), which has been adapted to improve its specificity to the Guinea variant viral strain causing the current outbreak by a two nucleotide swap in the VP35 siRNA and a single nucleotide swap in the L-pol siRNA to ensure complete match to the emergent strain. TKM-100201 and TKM 100802 have been evaluated in guinea pig, non-human primate, and human Phase 1 clinical trials. TKM-130803 treatment of Rhesus monkeys at 72 hours following challenge with a lethal dose of Ebola virus Guinea 2014 led to 67% survival (2/3 animals). TKM-100802 drug has received authorization for emergency use in EVD patients from the US Food and Drug Administration. 100 treatment courses of TKM-130803 are available for this trial, in a wet (non-lyophilized) formulation.

5.1. Efficacy data against EBOV

In-vitro activity of TKM-100802 was established using two variants of EBOV infecting a human hepatoma cell line. The EC50 ranged between <0.003nM and 0.04nM. The therapeutic index was determined to be between 1513 and >18954 for treatment commencing one hour following viral inoculation. Molecular analysis confirmed the proposed mechanism of activity of the two siRNAs. In-vivo trials have established that TKM-100802 has an inhibitory impact on virus replication in EBOV-infected cells, as well as a survival benefit in EBOV-infected guinea pigs and rhesus macaques[17,18]. In rhesus monkeys infected with a lethal challenge of Zaire EBOV, 100% survival has been observed in animals that received TKM-100802 at 0.5 mg/kg/day for 7 days, with 67% survival at 0.2 mg/kg/day for 7 days. In rhesus monkeys infected with a lethal challenge of Guinea variant EBOV at 0.5 mg/kg/day for 7 days, TKM-130803 (siEbola-3 with LNP1) resulted in 33% survival (1/3) with treatment commencing at 48 hours post inoculation, and 67% (2/3) with treatment commencing at 72 hours post inoculation.

5.2. Safety and tolerability

TKM-130803 has the same formulation as TKM-100802 except for two nucleotide swaps in the VP35 siRNA and a single nucleotide swap in the L-pol siRNA. Therefore the safety and tolerability profile is expected to be similar to TKM-100802. For a detailed description of the safety and tolerability of TKM-100802, please refer to the Investigator Brochure and additional safety summary. Adverse events (AEs) considered most likely to occur with TKM-130803 include discomfort, bruising, and bleeding at the site of infusion, and adverse effects analogous to those observed in animal studies, including coagulopathies, renal impairment, hepatobiliary injury and acute phase reaction.

In a human single ascending dose trial of TKM-100201 administered over one hour to healthy participants, infusion-related reactions, notably cytokine release syndromes (CRS) was observed. Clinical manifestations included flushing, headache, fever, hypotension, chills, nausea, and vomiting. Most of the reported treatment related AEs were consistent with a transient inflammatory response that begins within 6 hours after infusion and dissipates in most cases by 24 hours post-dosing. This interpretation is consistent with laboratory findings of transient elevations in some cytokines (e.g., MCP-1, IL-6, IL-1ra, IL-8) in some patients measured 2 and 6 hours post-dosing, returning to at or near baseline levels by 24 hours. In

addition, focal and reversible pain has been observed in association with liposomal or LNP therapeutics, particularly in the back, sacrum and sternum.

Fertility and reproductive toxicity studies have not been performed with TKM-100802 or TKM-130803.

5.3. Dosing and dosing rationale

In rhesus monkeys infected with a lethal challenge of EBOV, 100% survival has been observed in animals that received TKM-100802 at 0.5 mg/kg/day for 7 days, with 67% survival at 0.2 mg/kg/day for 7 days. In rhesus monkeys infected with a lethal challenge of Guinea variant EBOV at 0.5 mg/kg/day for 7 days, TKM-130803 (siEbola-3 with LNP1) resulted in 33% survival (1/3) with treatment commencing at 48 hours post inoculation, and 67% (2/3) with treatment commencing at 72 hours post inoculation. Comparisons of drug exposure data from monkeys and healthy human volunteers indicate that plasma exposure parameters (C_{max} and AUC) are approximately equivalent between humans and monkeys when dosed at the same dose level of TKM-100802 (based on weight; mg/kg). Thus, it is anticipated that dose levels of at least 0.2 mg/kg/day will be required to meet exposure targets derived from efficacious dose levels currently used in monkey EBOV infection studies.

The no-observed-adverse-effect-level (NOAEL) of TKM-100802 in a repeat-dose toxicity study in cynomolgus monkeys was 0.25 mg/kg/day after daily 1-hour intravenous infusion of TKM-100802 for 14 days. The total cumulative dose (3.5 mg/kg) at the NOAEL is equivalent to the maximum cumulative dose proposed in humans (i.e., 0.5 mg/kg/day × 7 days; 3.5 mg/kg). The highest dose level (1.0 mg/kg/day for 14 days) was well tolerated in monkeys, but resulted in microscopic findings in the liver, spleen, and kidneys, which were not severe, but precluded consideration of this dose level as a NOAEL.

The safety and pharmacokinetics of TKM-100802 have been investigated in a single ascending dose (SAD) phase of Study TKM-EBOV-002 (A Placebo-Controlled, Single-Blind, Single Ascending Dose Study With Additional Multiple Ascending Dose Cohorts To Evaluate The Safety, Tolerability, and Pharmacokinetics of TKM-100802 In Healthy Human Volunteers) over a dose range from 0.075 mg/kg to 0.5 mg/kg. The maximum tolerated dose (MTD) was determined to be 0.3 mg/kg, using a conservative toxicity grading scale for healthy volunteers.

TKM-130803 will be administered by IV infusion at a rate of 1.25 mL/min, administered over approximately 120 minutes for a total volume of 150mls administered. For infusion related adverse reactions, the infusion rate may be slowed or interrupted at the discretion of the treating physician. If the infusion is interrupted and resumed, the total infusion should be completed within 4 hours of infusate preparation. The patient will be in recumbent position for dose administration. TKM will be administered via a dedicated peripheral IV or central line.

For full details of infusion preparation and administration please refer to the relevant trial Standard Operating Procedure manual.

6. OBJECTIVES AND OUTCOME MEASURES

	Objectives	Outcome Measures

Primary	TKM: To evaluate the impact of TKM treatment on early mortality in EVD<br><br>Observational: To characterize the early mortality of EVD in an ETC	Mortality at Day 14 (in those that survive first 48 hours after admission)<br><br>Mortality at Day 14 (in those that survive first 48 hours after admission)
Secondary	TKM<br>1. To evaluate the impact of TKM-130803 treatment for adults on:<br>a) Time to recovery<br>b) Late mortality<br>c) Viral load<br>d) EVD symptoms<br>e) EVD antibody response<br>f) Long term clinical recovery<br><br>2. i)To assess the safety of TKM treatment for adults.<br>ii) To measure the pharmacokinetics (PK) of TKM following repeat dosing<br><br>Observational<br>To characterise the natural history of EVD in an ETC.<br>a) Time to recovery<br>b) Late mortality<br>c) Viral load<br>d) EVD symptoms<br>e) EVD antibody response<br>f) Long term clinical recovery	TKM<br>1. a) Time to meeting ETC discharge criteria.<br>b) Mortality at Day 30 and months 3,6,12 after first dose of study treatment<br>c) Viral load<br>d) Presence and duration of symptoms (Days 1-14)<br>e) Convalescent anti-Ebolavirus IgG titer (Day 30)<br>f) Clinical assessment at months 3,6,12.<br><br>2. Incidence of SARs, key adverse events (Days 1-14) and monitoring of vital signs (pulse rate, blood pressure, respiratory rate, temperature) pre, during and at 0, 1, 2, 4 and 8 hours post end of infusion<br>ii) PK pre-dose and at the end of infusion on days 1, 3, 5 and 7<br><br>Observational<br>a) Time to meeting ETC discharge criteria.<br>b) Mortality at Day 30 and months 3,6,12 after admission<br>c) Viral load<br>d) Presence and duration of symptoms (Days 1-14)<br>e) Convalescent anti-Ebolavirus IgG titer (Day 30)<br>f) Clinical assessment at months 3,6,12.

7. TRIAL DESIGN

7.1. Trial design

This research consists of a single-arm, open-label trial of TKM and a concurrent observational trial.

Ongoing observation of the clinical manifestations and outcomes of patients in ETCs is valuable, especially when data acquisition is undertaken in a systematic manner as part of trial.

7.2. Trial design rationale

Due to production capacity limits there are currently only 100 doses of TKM-130803 available for this trial.

The risk of infusion reactions means that TKM-130803 must be infused over a minimum 2-hour period during which clinical monitoring for infusion reactions is necessary. The intensity of clinical monitoring required means that infusions will, in most cases, only be undertaken in the morning (when the day shift

of clinical staff and the trial staff are all on site). Even then, the challenges of clinical care delivery within an ETC means that the number of participants who can receive TKM-130803 infusions and be safely monitored on any one day may be limited by staffing availability. ETCs may also vary in their capacity to administer TKM-130803 based on fluctuating patient-staff ratios, and inpatient numbers. Therefore, the maximum number of patients receiving TKM-130803 on any one day will need to be capped. The lead physician at the ETC will notify the maximum number of monitored beds available for the trial on any given day. Once the bed availability is known, if the number of patients eligible for TKM-130803 exceeds capacity they will be allocated to receive either the drug with standard care (as part of an interventional group) or standard care (as part of an observational cohort). The methodology for allocating cohorts is described in section 7.3.

7.3. Eligibility for TKM

Since TKM-130803 has never been used in children and the bio-distribution and pharmacokinetics is not known in this population, in the initial stage of the study, eligibility to the TKM arm will be restricted to patients 18 years of age or older until the first 15 patients have been enrolled and treated. At that time, all available and relevant safety data will be reviewed by the IDMC and a recommendation may be made to extend the eligibility to patients 5 years or older or to some other intermediate age between 5 to 18 years.

Similarly, there have been no fertility or reproductive toxicity studies on TKM – 130803. Hence pregnant and breastfeeding women will also be initially excluded from the trial. After the first 15 patients have been enrolled and treated, the data will be reviewed by the IDMC and the IDMC may recommend to include pregnant women or continue to exclude them from this trial. If the IDMC recommend inclusion of pregnant women, Ethical Review Board approval will be sought.

TKM is administered as an infusion. If reliable IV access cannot be obtained for a patient, they will not be eligible to receive the trial drug nor take part in the observational cohort.

7.4. Trial enrolment

Trial enrolment and allocation to treatment or observation cohort will take place as described in Figure 2 and below.

1. All ETC patients with a laboratory confirmed case of EBOV who can tolerate trial procedures, do not have organ failure and in whom IV access is possible will be eligible for enrolment into the observational trial.
2. Patients will also be assessed for eligibility for drug allocation according to the criteria in section 7.3.
3. Allocation into this drug eligible cohort will occur by the following process.
    i. Following arrival of the trial staff each morning, the maximal number of study beds (b) available for patients to receive study drug will be determined by the ETC clinical lead and the trial clinical lead.
    ii. Following consent of patients who have arrived or received PCR confirmation in the preceding 48hrs, each eligible patient will be sequentially allocated a number (n) from 1 to N, where N is the number of patients eligible.

iii. b random integers between 1 and N inclusive will be selected using statistical software. These patients will be allocated to the treatment cohort. The remainder of patients will remain in the observational cohort (when N>b).

iv. Whenever possible, the drug will be provided within 2 hours of trial team arrival in the morning, in order to provide adequate patient monitoring during the working day.

Figure 2. Process of assessing eligibility and patient allocation to treatment or observational cohort

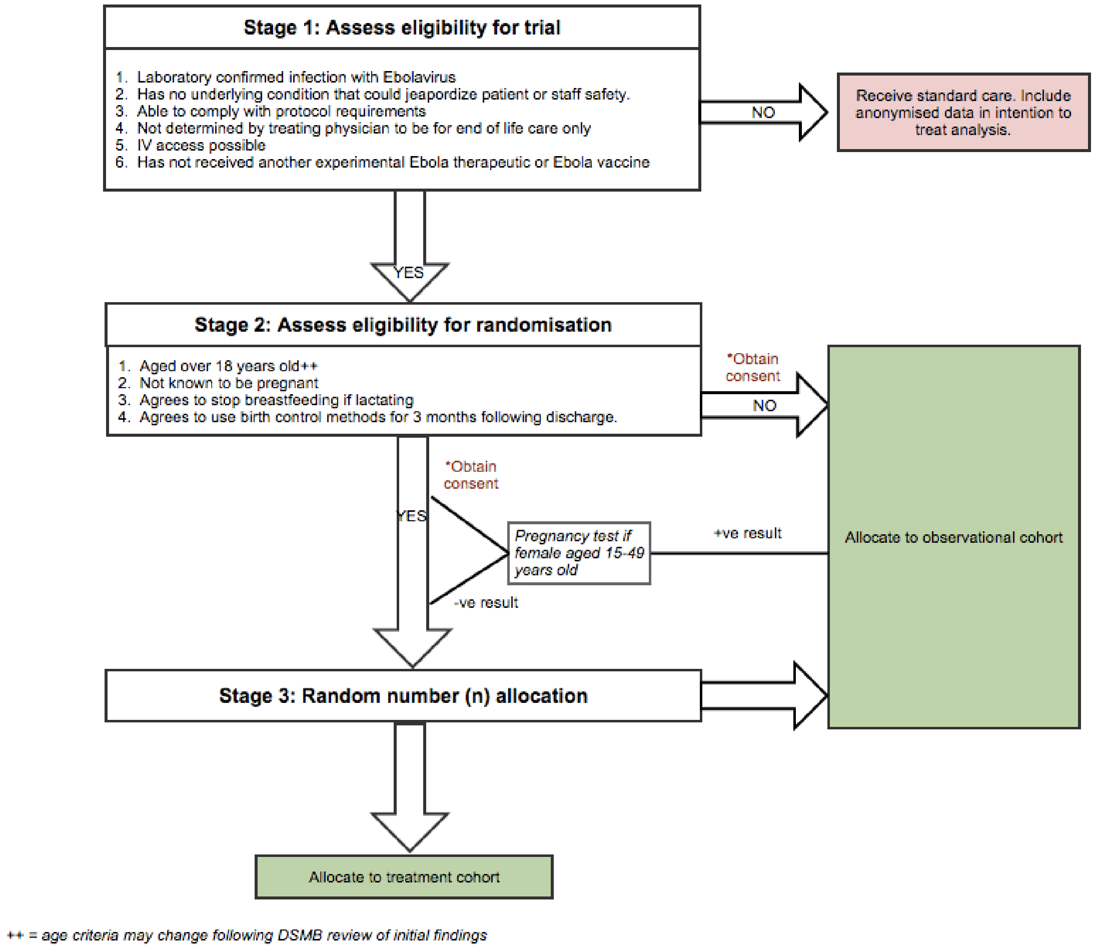

To assess the feasibility and safety of dosing with TKM-130803 in an ETU, data on the first four enrolled patients will be assessed by the IDMC prior to opening enrollment to additional patients. These patients (termed the safety cohort) may be recruited from one or multiple study sites. Each patient will receive at least 3 doses before dosing of the next patient starts. If a patient dies prior to receiving 3 doses, recruitment of the next patient may occur earlier. The IDMC will review data according to the schedule in the table 1 below. If the 4th patient dies prior to receiving 3 doses, the IDMC review may be brought forward. The safety cohort may be expanded following advice from the IDMC.

Table 1: Safety Cohort recruitment schematic.

													IDMC			
Patient 1	X	X	X	X	X	X	X									
Patient 2				X	X	X	X	X	X	X						
Patient 3							X	X	X	X	X	X	X			
Patient 4										X	X	X	X	X	X	X
Patient 5														X	X	X
Patient 6														X	X	X

**Patients 5 and 6 are recruited once recruitment is open following review by the IDMC.*

7.5. Participant inclusion criteria

The eligibility is a two-step process, first for the trial and then for the allocation to the drug eligible cohort

Eligibility criteria for the trial (stage 1):

- Laboratory confirmed infection with Ebolavirus
- No critical organ failure
- IV access possible
- No use of any investigational or non-registered product within 30 days prior to study enrolment or planned use during the study period.

From within this cohort, additional eligibility criteria for randomization (stage 2):

- Adult aged > 18 years (although the age range may be extended to >5years old following IDMC review of initial findings).
- Not pregnant (although this might change after IDMC review of the first 15 patients)
- Not breastfeeding (for lactating women)
- Agrees to use birth-control for 3 months post study.

The study of TKM treatment will concern the outcomes of all patients who are allocated to TKM treatment. For these patients, Day 1 will be the day of admission to the treatment centre. The primary response for the trial will be survival to Day 14 (yes or no). Patients who are allocated to TKM treatment and survive the first 48 hours after admission but do not actually receive it or who fail to complete the course of therapy, for any reason, will nevertheless be included in this primary analysis.

The observational study will include all patients who are admitted to the treatment centre during the period of the trial, including those considered eligible for TKM treatment but who are not allocated to it due to the limited number of available treatment beds. For the analysis of the outcomes of these patients, Day 1 will be the day of admission to the treatment centre.

Data from participants who for any reason are ineligible for the trial or refuse to participate will be included (anonymously) in the primary analysis of the observational group. All patient outcome data is routinely recorded in all ETCs and shared anonymously with WHO, therefore the use of anonymised day 14 outcome data is in line with current practices.

8. TRIAL PROCEDURES

8.1. Screening and informed Consent

Patients with reverse transcription polymerase chain reaction (RT-PCR) confirmed Ebolavirus RNA (confirmed patients) will be screened for trial eligibility and invited to give informed consent to participate, according to the procedures outlined in section 15.2. Staff will not discuss informed consent with patients (or the parents/representatives of patients <18 years old) who are not eligible for enrolment in any trial arm. The number of patients who are ineligible will be recorded anonymously. Study dosing, sampling and data collection will begin only after enrolment.

A patient may be enrolled in the study anytime within 48 hours of first arriving at the ETC with a confirmed diagnosis, or, within 48 hours of being informed of Ebolavirus positive PCR result, if this occurs while already at the centre. Therefore, if a patient has been excluded for a reason that resolves within this period, they may be approached upon resolution of the reason for exclusion. If a patient wishes to delay their decision regarding consent, they may enrol at any time within the 48 hour period.

A list of eligible patients will be produced each day (following receipt of the laboratory results).

8.2. Procedures for provision of TKM

Patients randomized into the treatment cohort will receive TKM-130803 in addition to the supportive therapy provided to all patients.

TKM-130803 will be administered at a dose of 0.3mg/kg by IV infusion at a rate of **1.25 mL/min** administered over approximately 120 minutes in a total volume of 150ml. Infusions should be administered using an infusion pump, to ensure a well-controlled rate of infusion.

For full details of infusion preparation and administration please refer to the trial Standard Operating Procedure for drug administration.

The recruitment process for the safety cohort is described in Section 7.4, and the checking of safety data from the previous patient against safety rules set by the IDMC prior to dosing the next patient. The IDMC will review data from the complete cohort prior to recruitment being opened to all suitable patients. The safety cohort may be expanded following advice from the IDMC.

Doses will be administered once daily, for seven days. In the event of suspected drug-related toxicity or a change in the patient's clinical condition, the dose of TKM-130803 may be reduced to a minimum of **0.24 mg/kg** once daily at the discretion of the treating physician. If the infusion is interrupted and resumed, the total infusion should be completed within 4 hours of infusate preparation. The patient will be in recumbent position for dose administration.

Standard safety assessments of patients will include monitoring of vital signs (pulse rate, blood pressure, respiratory rate, temperature), symptoms and SARs. Vital signs will be assessed at the following approximate time points: pre-infusion, during the infusion (preferably between 30-90 minutes), at the end of infusion, and at 1, 2, 4 and 8 hours post end of infusion, as well as at additional time points if indicated by the patient's clinical condition.

There are two potential adverse outcomes that require close clinical supervision.

1. Infusion related reactions may occur following commencement of the infusion. As the drug is given once a day, the timing of administration will be set to coincide with the presence of the trial staff in the treatment area. This will allow monitoring of symptoms and a decision to slow or halt the infusion if necessary. Pre-medication such as paracetamol may be given. IM adrenalin will also be made available for the event of an anaphylactic reaction.

2. Cytokine release syndrome may occur several hours after the infusion commences. Health care workers involved in care of patients receiving TKM will receive additional training to recognise the constellation of symptoms that are associated with it (flushing, headache, fever, hypotension, chills, nausea and vomiting). Management of a cytokine related event will prioritise immediate care (i.e. stopping of infusion (if ongoing), provision of IV fluids) and prompt medical review of the infusion speed and dose. Additional drugs (including steroids) may be given to ameliorate symptoms, at the treating physician's discretion. Procedures for safety reporting are outlined in 11.2.

8.3. Laboratory Samples

All patients, both in the observational and TKM cohort, will have regular blood samples drawn and analysis performed; see Section 9.2 Laboratory Assessments. Also, any residual volumes will be stored. Those in the observational cohort will have all the laboratory tests as for those in the TKM cohort; malaria, pregnancy (for women of childbearing age), ebola testing, electrolyte, renal and hepatic function testing. However, they will not have PK samples taken; these will only be taken from patients receiving the trial drug.

8.4. Procedures for standard care

The supportive therapy received by patients will not be affected by participation, or non-participation, in the trial. All patients will receive standard supportive therapy and this will be recorded. Treatment provided by clinical staff is based on the ETC guidelines for management of EVD. This includes oral and/or intravenous fluid resuscitation, empirical antibiotics, antimalarial agents, and also symptom control, which includes antiemetics, antipyretics/analgesics, and anti-diarrhoeal agents, as appropriate to the patient's symptoms.

8.5. Treatment Interruption

In the event of a SUSAR or other significant treatment related event, the treating physicians will determine if study treatment will continue on the basis of clinical picture and severity and resolution of the event. Consultation with the Independent Data Monitoring Committee will be made in the case of uncertainty to determine if the treatment should be discontinued.

If there is an indication of a cytokine response in the safety cohort (first 4 patients) then recruitment will be reviewed. The IDMC will review data from the complete cohort prior to recruitment being opened to all suitable patients. The safety cohort may be expanded following advice from the IDMC.

8.6. Discontinuation of Trial Treatment

Each participant has the right to discontinue use of study treatment at any time. In addition, the Investigator may discontinue treatment at any time if the Investigator considers it necessary for any reason including:

- Ineligibility (either arising during the trial or retrospectively having been overlooked at screening)
- An adverse event which requires discontinuation of the trial medication
- Disease progression which requires discontinuation of the trial medication
- Patient or physician decision

The reason for any discontinuation of treatment will be recorded in the case record form.

8.7. Withdrawal from the Study

Patients, or parents/guardians/representatives when they have given consent on behalf of the patient, are free to withdraw consent and stop study participation at any time for any reason. In this case, the patient's clinical management will continue to be provided according to standard care and will not be affected by the decision to withdraw. Outcome data and the reason for withdrawal will be recorded when possible. Data collected up to the date of withdrawal will be used in study analysis.

9. DATA COLLECTION

9.1. Clinical data acquisition

Study dosing, sampling and data collection will begin after enrolment. Figure 3 shows the clinical data acquisition schedule.

Patient demographic data and medical history will be collected for all patients at the time of enrolment in the trial. While a participant is an inpatient, information will be collected daily on signs and symptoms, medications and blood products received, and the results of non-trial blood samples. For patients who are receiving TKM that day, detailed records of physiological monitoring and adverse event monitoring (outlined in section 8.2) will be provided.

These data will be collected daily for participating patients up to Day 14, or discharge, or death, whichever occurs first. Patients who achieve discharge criteria before day 14 will stop the inpatient testing schedule upon discharge.

For patients not enrolled in the study, only anonymous survival data will be collected.

Discharge criteria are:

- 72 hours without fever or significant symptoms AND
- A significant improvement in clinical condition AND
- Able to feed, wash and walk independently AND
- Ebolavirus RNA PCR test negative

Figure 3: Clinical data acquisition procedures

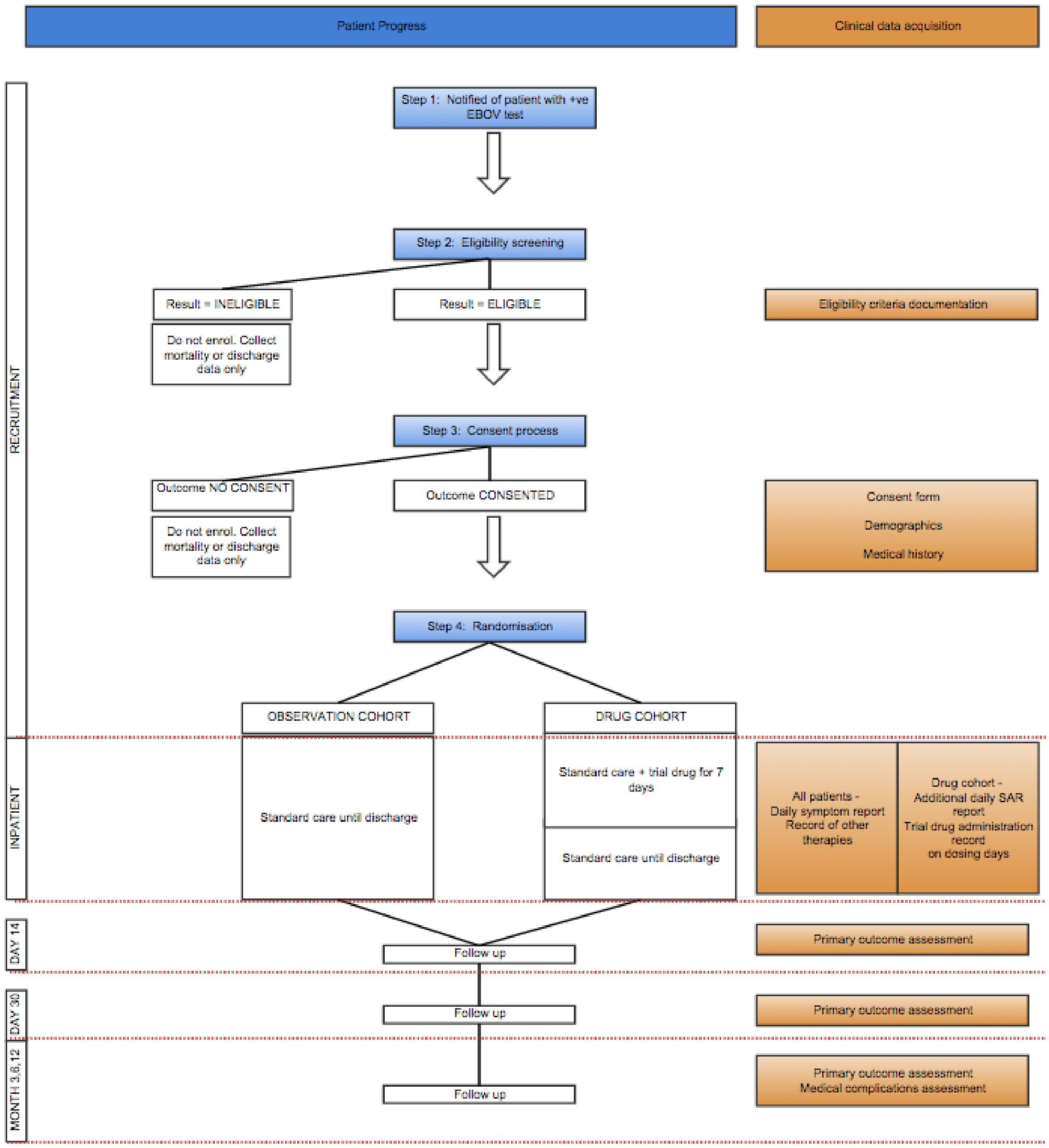

9.2. Follow up Assessments (Day 14, Day 30, Months 3, 6, 12)

Survival at Day 14 is the primary end point of this trial, and defined as survival until past midnight (00:00hrs) on Day 1. Follow up assessments will be conducted on Days 14 and 30 and at 3, 6 and 12 months. Patients who have been discharged before these dates will be issued a mobile telephone for the purposes of follow-up. Patients will be invited to return to the treatment centre for the Day 14 and 30 visits and then subsequent follow up will occur by phone or at a treatment centre. When patients are not able or willing to attend a follow-up visit on Day 14 and 30 at the treatment centre, field workers will follow up by phone. Remaining follow up visits will be arranged at a survivor's clinic or suitable local health facility. Patient status on follow-up assessment days will be collected (admitted, met discharge criteria, deceased). In addition, visits at 3, 6, and 12 months will seek information on symptoms post recovery.

Primary/secondary outcome data for these visits can be collected at any point after the respective days.

9.3. Laboratory Assessments

The timing of laboratory sampling for research purposes is outlined in Figure 4..

Figure 4: Scheduling sample for trial specimens – TKM study.

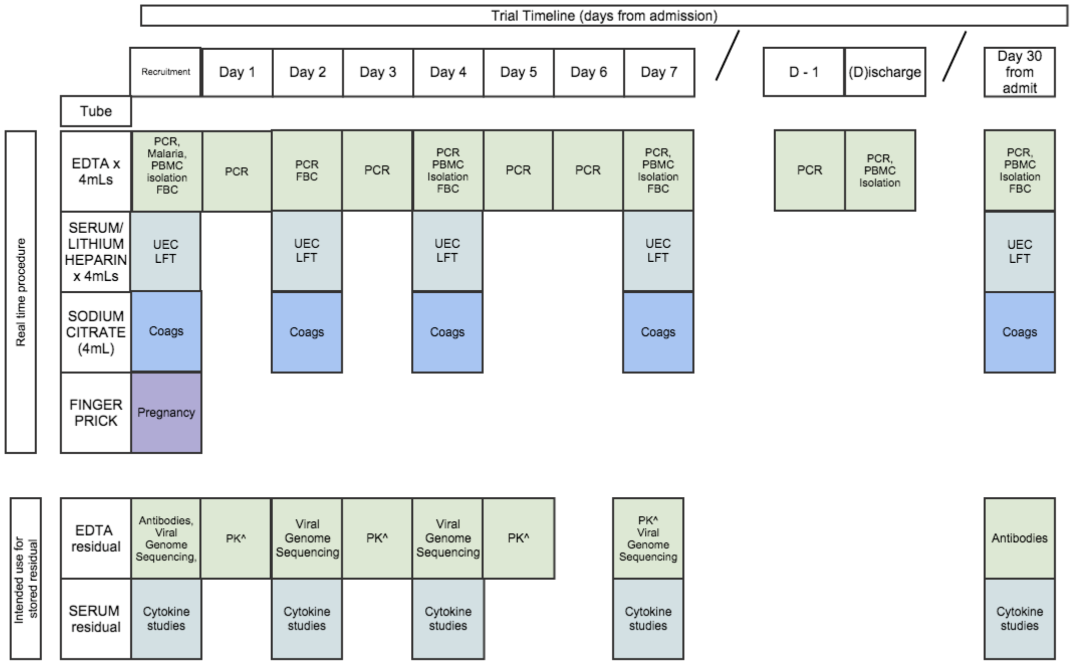

* Timing assumes that patients commence infusion on day 1, following the recruitment day. If commencement of infusion occurs at a later time, the schedule will be altered to reflect this.
^ PK testing only for interventional protocol and if laboratory capacity exists.

The only essential blood tests are the diagnostic EBOV positive PCR on admission and repeat EBOV negative PCRs prior to discharge. Adult and pediatric samples will be reduced in volume according to standard procedures such that no patient will have more than 0.6 mls/kg (>1% blood volume) taken on any one day, nor more than 2.4 mls/kg (approximately 3% of blood volume) taken in any four week period. Standard care samples will be prioritized over research samples if volume reduction is required. Ability to take samples is dependent on staff availability, the availability of suitable laboratory facilities and caseload. Research samples and standard care samples may be reduced where required, to maintain care standards and staff safety, and reflect the assays that can be performed by the laboratory attached to the ETC. Other chemical or biological assays may be performed using the same blood volumes if available and useful to inform patient safety or study outcomes.

The same sampling schedule applies to all patients irrespective of their allocation in either the observational or TKM cohort, with the exception of pharmacokinetic drug testing. This will only be performed on patients receiving the trial drug.

Malaria testing: Malaria diagnostic tests will be performed on the triage blood sample as part of standard care. The results of these tests will be recorded.

Pregnancy testing: For women of childbearing age (15-49 years) a chromatographic βHCG test will be performed on the triage blood sample. Urine testing is also acceptable.

Ebola testing: Patients will have blood samples collected at triage for EVD diagnosis by PCR and prior to discharge to confirm clearance of viral RNA, both as standard care. In addition, a study only EDTA will be taken daily for the first 7 days of study involvement for PCR. The PCR result for all samples, will be shared

with the clinical team in real time. Samples obtained for PCR are typically ≤4 ml whole blood in an EDTA tube; finger or heal-pricks of blood on a dry swab are sometimes obtained when venepuncture is not possible. Ebolavirus RNA will be detected by PCR, as per local clinical laboratory protocols. Additionally, plasma anti-Ebolavirus antibody titers will be determined by analysis of residual volumes by partner laboratories. This is to determine whether the treatment influences the subsequent production of specific antibodies.

Electrolyte, renal and hepatic function testing. Additional testing on days 1, 2, 4 and 7 will investigate electrolytes, and renal and hepatic function. Electrolyte disturbances are a common complication of Ebola. Results from toxicological studies indicate TKM may cause hepatic impairment, although this has not been shown in human trials. This information will be provided to clinical staff in real time.

Pharmacokinetic testing. Pharmacokinetic (PK) testing will only be undertaken on patients receiving the study drug. In order to assess the PK of TKM following repeat dosing, it is recommended that blood samples be obtained for PK analysis, if feasible, depending on the capabilities of the ETC, the availability of appropriate laboratory facilities and the clinical condition of the treated individual. It is recommended that PK samples be obtained pre-dose and again at the end of the infusion, on Days 1, 3, 5, and 7 of treatment. The PK samples should be obtained, processed and stored according to the instructions provided separately, entitled, “Instructions for the Collection, Processing, and Storage of Blood Samples for Pharmacokinetic (PK) Testing” - PK Manual.

Residual volumes. Residual volumes of all samples will be stored and subsequently shipped to international partner laboratories for confirmatory testing and quantification. If volume remains, additional testing relevant to the pathogenesis of EVD or effects of the study treatment will be performed. Residual cells or buffy coat and plasma supernatant buffy coat may be retained for host genetic studies to identify susceptibility and severity markers. Details of sample storage, shipment and custody are in Section 10.2.

10. TRIAL DRUGS

10.1. Formulation

The investigational product, TKM-130803 Injection is presented as an aqueous dispersion of nucleic acid/lipid particles (also referred to as Lipid Nanoparticles, LNP) in a 10 mL United States pharmacopeia (USP) Type I borosilicate glass vial with a Fluorotec®-faced butyl rubber stopper and an aluminum flip-off cap. TKM-130803 is a formulation of siEbola-3 drug substance with lipid excipients. The nucleic acid/lipid particles (LNPs) have an average size of approximately 60 to 90 nm.

The nominal mg/mL drug concentration of the specific batch of TKM-130803 will be recorded on each vial and also on the corresponding storage cartons. **TKM-130803 vials should remain in the cartons protected from light and stored refrigerated at 2 to 8°C** (35.6 to 46.4°F). **DO NOT FREEZE.**

TKM will be administered by a two hour IV infusion. The patient will receive 150mL of TKM-130803 dosing solution in 0.9%NaCl. An infusion pump should be used when administering TKM-130803 to ensure a well-controlled rate of infusion.

For full details of infusion preparation and administration please refer to the relevant trial Standard Operating Procedure manual.

10.2. Storage and Accountability

TKM vials should be stored refrigerated at 2 to 8°C (35.6 to 46.4°F). They must not be frozen.

Inventory, dispensing and accountability of study treatment will be tightly controlled. Treatment dispensed to the Confirmed ward, but not consumed by patients will not be returned due to infection control procedures and will be destroyed. Full accountability procedures and logs will be detailed in the corresponding study reference manual.

11. SAFETY REPORTING

Due to the nature of the symptoms of EVD (see **Table 2**) [2] and the relatively small sample size it will be very difficult to differentiate between symptoms of the disease and events due to treatment, and the trial will have limited power to do this. Our investigation of safety will therefore focus on Serious Adverse Reactions (SARs) and Suspected Unexpected Serious Adverse Reaction (SUSARs) and key adverse events (e.g. diarrhoea, vomiting, bleeding, allergic reaction, anaphylaxis, cytokine release, flushing, hypotension and pain (chest pain, back pain, abdominal pain and headache). We will collect data using the CRF on these symptoms if they are new or worsening including, as far as possible, a severity grading in line with the CTCAE (Common Terminology Criteria for Adverse Events). These will only be reported to TOG/IDMC in accordance with the safety reporting as described below.

11.1. Definitions for safety reporting

Serious Adverse Reaction (SAR) – A serious untoward and unintended response in a participant to the study treatment, which is related (or has a reasonable possibility of being related) to any dose administered to that participant.

To qualify as "serious" the response must meet one of the following criteria:

- results in death
- is life-threatening
- requires inpatient hospitalisation or prolongation of existing hospitalisation
- results in persistent or significant disability/incapacity
- consists of a congenital anomaly or birth defect.

Other 'important medical events' may also be considered serious if they jeopardise the participant or require an intervention to prevent one of the above consequences.

For this protocol, due to the expected high death rate due to EVD, medical events that are not considered related to any dose of study treatment are *not* considered to be Serious Adverse Events (SAEs).

Suspected Unexpected Serious Adverse Reaction (SUSAR) - A serious adverse reaction, the nature and severity of which is not consistent with the information about the medicinal product in question set out in the investigator's brochure (IB).

11.2. Procedures for safety reporting

All SARs will be reported by the site to the operational team within 48 hours. In discussion with the site staff, the operational team will assess the SAR for expectedness and relatedness and report all SUSARs to the independent data monitoring committee (IDMC) immediately. The operational team will also report any SARs and SUSARs to all the relevant parties (such as IRBs, regulatory authorities and Tekmira) as required within 7 days.
Evaluation relatedness of reactions will consider the opinion of the site staff and the expected signs, symptoms and events associated with Ebolavirus disease or trial treatment according to the relevant Investigators' Brochure. Evaluation of expectedness will be made with reference to the Investigator's brochure. Serious infusion reactions will be reported. While the clinical syndrome of cytokine release may mimic other symptoms of EBOV, if suspected and serious, it will be reported to the TOG/IDMC.

Table 2. *Symptoms of Ebolavirus disease (WHO Ebola Response Team, 2014[2]; ISARIC WHO Viral Haemorrhagic Fever CRF)*

Known symptoms and clinical events of EVD:	
Death	Coma or unconsciousness
Fever	Unexplained bleeding
Fatigue	Hematemesis
Loss of appetite	Blood in stool
Vomiting	Bleeding gums
Diarrhoea	Bloody nose
Headache	Bloody cough
Abdominal pain	Other bleeding
Joint pain	Bleeding at injection site
Muscle pain	Blood from vagina
Chest pain	Blood in urine
Cough	Bleeding under the skin/bruising
Difficulty breathing	Back pain
Difficulty swallowing	Decreased urine output
Conjunctivitis	Lower chest wall indrawing
Sore throat	Hearing impairment
Confusion	Tinnitus
Hiccups	Seizures
Jaundice	Hepatomegaly
Eye pain	Splenomegaly
Rash	Lymphadenopathy

12. STATISTICS

12.1. Description of Statistical Methods

The trial is designed to determine whether TKM is a promising treatment for Ebola. No early stopping in the case of evidence of efficacy is proposed, as there are currently only 100 courses of TKM available so early general roll out of the treatment is not possible. Enrolling the full 100 patients will maximize the precision of the final estimate of efficacy.

A futility design (Whitehead and Matsushita, 2003) is therefore used to allow early stopping in the event of evidence of futility or harm. This approach will recruit up to 100 patients, but will stop if the number of successes observed so far falls below a certain threshold.

In order to avoid early stopping due to enrollment of patients with very severe, late Ebola virus disease, who may not be expected to be salvaged even by an effective antiviral therapy, the stopping rule will be calculated after exclusion of enrolled patients who die within 48 hours of admission to the ETU.

For the purposes of determining futility, the effectiveness of the treatment will be judged in terms of the probability that a patient allocated to TKM treatment will survive to Day 14, after excluding patients who die within 48 hours of admission to the ETU. This probability will be denoted by p. Note that p represents the true value of the probability rather than any estimate that might be found from the trial data. The value p = 0.55 will be used for guidance. If p ≤ 0.55, then the treatment will be regarded as 'not promising' and the trial will be terminated.

The choice of guide value for p has been made following an analysis of individual level data on patients from a Ebola Treatment Centres from this current outbreak at Gueckedou, Foya, Kailahun and Elwa 3. Data on the outcomes of 1592 patients with laboratory confirmed Ebolavirus infection from these four centres are shown in Table 3. Only data from adults have been included, and data on patients who died on the day of admission or on the following day have been omitted.

Table 3: *Estimates and 95% confidence intervals for the probability of surviving to Day 14 following admission to the treatment centre, excluding patients who die on the date of admission or on the day after*

	**Gueckedou**	**Foya**	**Kailahun**	**Elwa 3**	**Overall**
Number of subjects	431	233	361	567	1592
Lower limit	0.431	0.383	0.455	0.447	0.459
Estimate	0.478	0.446	0.507	0.489	0.484
Upper limit	0.525	0.510	0.559	0.530	0.508

As a result of this analysis, p = 0.55 has been set as the success rate threshold above which TKM would be considered promising. A margin has been allowed between the upper confidence limit found in Table 2 for the overall data and this threshold. This allows for any drift towards better outcomes as treatment centres become more experienced and less congested, and ensures that only a non-trivial treatment effect is considered promising.

While children are initially excluded from the TKM arm of the trial, if the eligibility criteria is expanded to age >5 following IDMC review of preliminary results, TKM trial data from adults and children will be jointly analysed, since the availability of TKM is restricted to 100 treatment courses and a stratified analysis is infeasible. Case fatality is highest in children aged < 5 years, after which it declines with age reaching a minimum in persons aged about 15-20 years. Therefore the inclusion of subjects aged 5-17 years is not expected to inflate the case fatality, and the futility threshold of p ≤ 0.55 is expected to remain valid.

12.2. The Number of Participants

The maximum number of participants available to evaluate the drug efficacy is 100. The data will be analysed sequentially and stopping rules applied.

12.3. Criteria for the Termination of the Trial

Research sites will inform the data management centre every time that a patient is allocated to TKM treatment. Fourteen days after admission, they will report to the data management centre whether that

patient did, or did not, survive to Day 14. Every time that a Day 14 report is received on a patient, a point will be plotted on Figure 5**Error! Reference source not found.**. This is plot of the number of survivals (S) reported so far against the number of Day 14 reports received (n), after excluding from both S and n those patients allocated to TKM treatment who died within 48 hours of admission. The plot is compared with the lower red boundary shown. If the plot reaches this boundary, the trial will be stopped with the conclusion that treatment with TKM is not promising. This safety plot will ensure a rapid reaction if TKM proves to be ineffective or harmful in the short term.

If the plot continues above the boundary until the green boundary is reached, corresponding to Day 14 reports being received from 100 patients, then it will be concluded that TKM is a promising treatment. If the trial has to be terminated before 100 patients have received TKM and before the lower red boundary is reached, and if stopping is due to a shortage of eligible patients rather than to any safety concerns, the final dataset will be analysed. If the outcomes of patients recruited are good enough it remains possible for TKM to be found promising, even following an incomplete study. Nevertheless, every effort will be made to complete the study of 100 patients if the lower boundary is not crossed and no important safety concerns emerge.

Figure 5*. The stopping rule for the trial*

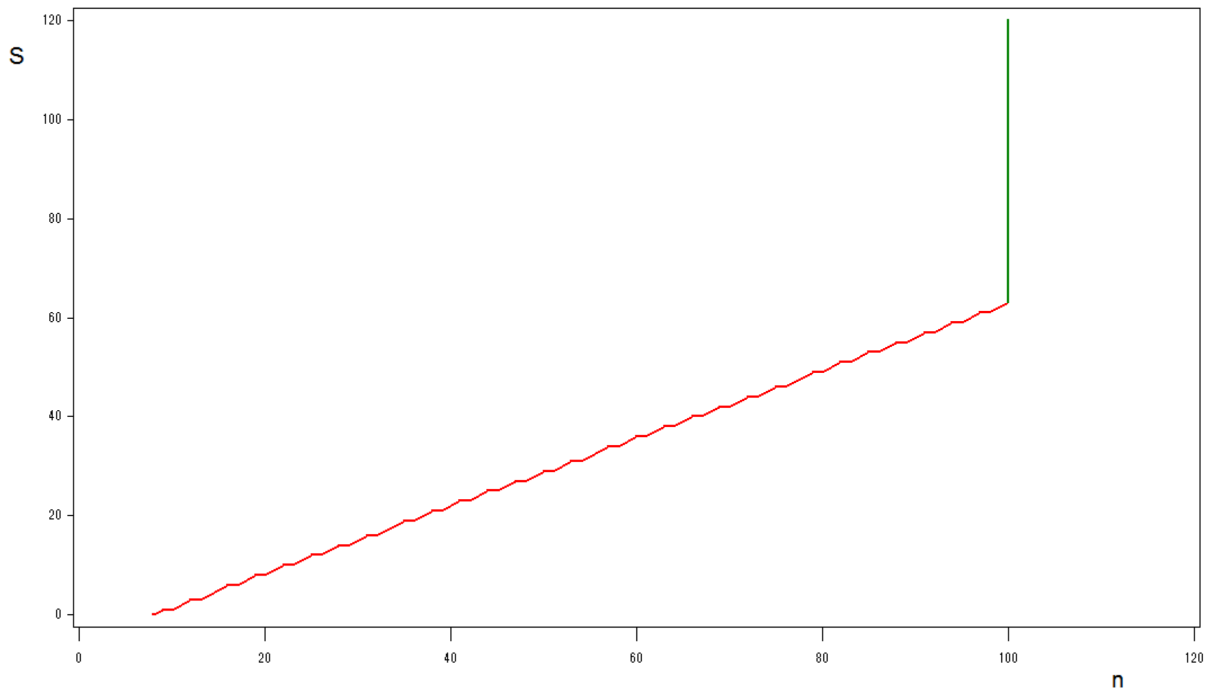

The properties of the design have been calculated exactly, based on the independent Bernoulli distributions of each patient outcome. These are shown in Table 4 and depict the specified probability of reaching the most appropriate conclusion for a range of values of p. It can be seen that the probability is declaring TKM to be promising is set at 0.025 according to this design. Recommending a treatment associated with such a low probability of survival to Day 14 would be considered a type I error, and the risk of this has been set at the conventional value of 0.025. The power of correctly identifying TKM as promising when p = 0.70 is 0.827.

Table 4: Probabilities of claiming that TKM is promising for the phase II trial of Treatment TKM

True probability of success (p)	Probability of claiming that TKM is promising
0.50	0.002
0.55	0.025
0.60	0.158
0.65	0.487
0.70	0.827
0.75	0.973
0.80	0.998
0.85	1.000
0.90	1.000

12.4. Final Analysis

When the trial has been completed, a point estimate and a 95% confidence interval will be computed for p using the method of Jovic and Whitehead,[19] which allows for the sequential nature of the trial.

12.5. Inclusion in Analysis

It is important to note that the primary sequential analysis described uses only data from patients who were recruited more than 14 days previously. Although it may be known that some more recently recruited patients have already died, their data will not yet be used, to avoid bias due to information on death being available sooner than information on survival. Enrolled patients who die within 48 hours of admission to the ETU will be excluded from the analysis.

12.6. Procedure for Accounting for Missing, Unused, and Spurious Data.

While considerable effort will be made to ensure all patients are followed up at Day 14, Day 30, month 3, 6 and 12 (through return visits to clinic, phone, email or field-researcher visits), some patients may not be contactable. Leeway will have to be allowed for late reporting, but this should be limited: it may be that data on survival to Day 14 is not entered until Day 16 in order to avoid remaining bias due to late reporting of survival. However, if it turns out that a patient died on Day 15 or later, *then they are survivors* for the purpose of this study. If they walked away on Day 7, seemingly cured, it might be assumed that they survived to Day 14. Some judgment will necessarily be used here.

12.7. Rationale for statistical design choice

In the context of the current EVD outbreak, a simple, robust and rapid trial is essential. Mortality dominates all other patient endpoints. Most deaths occur within 14 days of diagnosis, and so survival (or not) to Day 14 represents the most relevant clinical endpoint. The situation at Ebola Treatment Centres is highly challenging, and so this study places minimal additional responsibilities on them.

A design with a lower stopping boundary is proposed so that, if the trial is to conclude that TKM is not promising, this can be done as quickly as possible. If all treated patients die before Day 14, then only 8 patients will be required. No early stopping in the case of evidence of efficacy is proposed, as there are currently only 100 courses of TKM available so that no early general roll out of the treatment is possible. Rapid reporting of minimal information would appear to be feasible, and in keeping with the urgency of

the situation. Until the trial is terminated, data managers need only compare the number of survivals observed so far with the stopping boundary**Error! Reference source not found.**. A table of values will be provided for this purpose.

The approach described is a form of the futility design, as described by Whitehead and Matsushita [23] The analysis is based on an orderings approach introduced by Fairbanks and Madsen.[24] Exact calculations have been made for this particular design, using SAS, and this package will be used for the analysis.

13. DATA MANAGEMENT

13.1. Data Capture

Patient files will serve as source clinical and drug dosing data for the study. Due to infection control procedures, paper documents cannot be taken outside of the high-risk containment zone; therefore records may be dictated to a secondary document outside of the zone and the primary document incinerated. Where possible, electronic data entry will capture source data directly or source documents will be photographed/scanned and archived. Original data files from the site and research laboratories will provide source data for laboratory testing results. Data recorded by clinical staff as a part of the standard case investigation form will be used to support the classification of disease severity as well as for validation and comparison of study data.

Data will be entered to a central study database on MACRO software. Data management, reporting and storage within this trial will comply with the requirements of European Union data protection laws, ICH Good Clinical Practice and FDA 21 CFR Part 11.

13.2. Access to Data

Data sharing will be under the aegis of the Trial Steering Committee and will adhere to the principles of rapid, open access as outlined in the World Health Organisation report on Ethical considerations for use of unregistered Interventions for Ebolavirus disease.[25]

14. QUALITY ASSURANCE PROCEDURES

Governance of the trial will be through the Trial Steering Committee (TSC), the Trial Operation Group (TOG) and the Independent Data Monitoring Committee (IDMC).

- The TSC will be the primary decision making body, with membership from each partner institution, a senior representative from each participating country, and other independent experts. Members of the TSC are given in Section 1 (members may change if necessary). Decisions of the Committee will be by consensus. WHO will be part of the Committee on an ex-officio basis. Terms of reference for the TSC will set out the full details of the membership, operations and remit.
- The TOG will be led from the ISARIC Coordination Centre in Oxford and report to the TSC. This team will provide operational oversight and day-to-day management of the trial to ensure compliance to follow up and critical factors such a retention and data quality. This team will comprise those with responsibility for the operational activities and so include representation from each site, data management, trial operations, training and logistical support, laboratory sample processing and handling.

- The IDMC will be responsible for reviewing the data from the study. Membership will include individuals with statistical, clinical and trial expertise. An IDMC charter will fully set out the committee's remit, membership and full definition of their operational conduct in terms of reviewing efficacy and safety data and reporting their recommendations to the TSC.

The trial will be conducted in accordance with the current approved protocol, relevant regulations and standard operating procedures. Regular monitoring will be performed to ensure quality control. Data will be evaluated for compliance with the protocol and accuracy. Following written standard operating procedures, the monitors will verify that the clinical trial is conducted and data are generated, documented and reported in compliance with the protocol and the applicable regulatory requirements.

The MACRO software will be configured to automatically cross-validate across entire patient records according to the specific requirements. Data Clarification requests will be built in to the system. The data validation and quality processes will comply with the new FDA and EU recommendations for data level trial monitoring and therefore allow for data quality to be assured in real time as a mechanism for onsite monitoring.

Investigators and clinical staff treating centre are required to provide direct access to source data/documents for trial-related monitoring, audits, ethics committee review and regulatory inspection. All trial related and source documents must be kept for at least five years after the end of the trial.

15. ETHICAL AND REGULATORY CONSIDERATIONS

15.1. Regulations, Guidelines and Approval

The Investigators will ensure that this study is conducted in compliance with the current revision of the Declaration of Helsinki and the applicable principles of International Committee on Harmonization Good Clinical Practice Guidelines. Regulatory approval to conduct the study and import investigational products will be obtained from national authorities. The protocol, informed consent form and investigators brochure will be submitted to the Sierra Leone Ethics and Scientific Review Committee , the Oxford Tropical Research Ethics Committee, and where necessary, the Ebola Treatment Centre funding organisations ethics board, for written approval.

The Chief Investigator will submit and, where necessary, obtain approval from the above parties for all substantial amendments to the original approved documents.

15.2. Considerations in Informed Consent

STANDARD CARE UPON PRESENTATION: Patients are triaged to Suspected and Probable wards upon presentation based on symptom and contact history. Blood is drawn for Ebolavirus testing according to standard care at the site. Results are reported back to the clinical staff, who then visit patients to discuss the results. When laboratory results positive for Ebolavirus are received from the laboratory, a senior member of medical staff and a psycho-social worker visit the patient to discuss the results. Clinical and social counseling are provided as the patient is moved to the Confirmed ward. Recruitment to the study will not interfere with the standard care process. Research staff will be available to support ETC workers for study related tasks and to ensure that standard care is not disrupted by study procedures.

PROCESS FOR TAKING INFORMED CONSENT: Written informed consent to participate will be required from all participants or their representative. The staff who undertake the discussion of test results will decide when the patient should be approached regarding the study. This discussion should occur as soon as possible after the availability of confirmed results, but at a time when the patient is emotionally stable and the staff feel that they are capable of considering the risks and benefits of participation. Informed consent can be delayed for up to 48 hours following arrival to the ETC.

All eligible patients (section 7) should be offered the opportunity to participate in the study. When the clinical staff and/or social workers agree that an adult patient is mentally and physically fit to discuss and consider participation in the study, the patient or the parent/guardian of a patient under 18 years, will be approached. If an eligible patient has reduced consciousness or is unresponsive to discussion due to illness severity, an appropriate representative will be approached for consent if available. Appropriate representatives will be selected according to the standard Ebola Treatment Centre processes for determining who can make decisions on a patient's behalf. In the case of a child who has no parent/guardian or in the case of a patient who is unfit to consent and there are no appropriate representatives available proxy consent will be sought from an independent adult, or group of adults (two or three) who have agreed to act together to give proxy consent. This person/ group member could be a doctor who is not a member of the research team, or other suitable adult willing to act in this role, who has already agreed to undertake this role and has been fully briefed on the trial.

Consent will be requested by the local study staff or another suitable qualified and trained person authorised to do so by the Principal Investigator.

ADULTS: Adult patients for the purpose of consent are defined as ≥18 years of age. Adults who are deemed fit to discuss and consider participation by the medical and social staff will give consent independently. If an adult is not considered mentally and physically fit to give informed consent, consent will be sought from a close relative who attends the clinic or an appointed representative. When consent is obtained from a relative, the patient will be approached for consent if at any time during study participation their condition improves such that they can consider consent.

CHILDREN: Children are patients less than 18 years of age. Consent for children to participate in the study will be sought from the parents or guardians of the child or an appointed representative in the case of a child without a contactable parent/guardian. Study participation will be discussed at the discretion of the clinical and social staff with children of suitable physical and mental fitness as well as emotional maturity. When consent is obtained from a representative, a parent/guardian will be approached for consent if at any time during study participation the study staff are able to contact them.

DISCUSSING CONSENT: Participants or their representative will be presented with an informed consent form that explains the purpose, methods, risks and benefits of the study. The form will be available in relevant written local languages. If the patient or representative cannot read, the form will be read to them in the presence of a witness when available. If a consent form is not available in a language familiar to the participant, staff will verbally translate the form into the language of the patient. Standardization of translation will be supported by the use of recorded verbal translations with appropriate language and explanations in local terms that will be used to train study staff. When possible, a second member of staff will be present during the informed consent discussion to ensure that translations are appropriate, the form is read accurately, that all of the patient's questions have been addressed and to act as witness when possible. This will be facilitated by the paired working system at the treatment centres, but may not always be possible due to human resource limitations and limited shift times.

DOCUMENTING CONSENT: Patients or their representative will be asked to sign and date an informed consent form prior to participation. Those unable to write will be asked to make a mark on the signature line only. If a witness was present during the consent process s/he will co-sign the consent form to confirm the accurate reading of the form and the consent of the participant/parent/guardian/representative. If consent for an adult patient is given by a representative, patients will be asked to sign a separate informed consent form when/if they are deemed physically and mentally fit at a later point during the study. When a second staff is present for the informed consent discussion, s/he will sign the informed consent form. A child's assent to participate will not be recorded.

ARCHIVING OF INFORMED CONSENT FORMS: Due to infection control procedures, paper cannot be transferred externally from the confirmed wards. Therefore informed consent forms will be photographed and archived electronically, then destroyed according to waste disposal procedures for contaminated substances. Patients will be given either a printed copy of the original signed form if possible or an unsigned copy to retain upon discharge.

15.3. Participant Confidentiality

The trial staff will ensure that the participants' anonymity is maintained. All study documents and samples will be labeled with an anonymous study code. Identifying information collected as a part of this study will remain confidential. For the purpose of ensuring data integrity and to facilitate quality assurance, study records will link to clinic files, which include patient's name and other identifying information. Participants' names will be recorded at the time of enrolment to allow for their identification at follow-up visits. However identifiable information will be linked to stored data or samples only by a protected Master List. This list will not be shared outside of the site study staff and no identifying information will be transferred between sites. All CRFs and samples will be labeled with a study identification number only and stored in suitable secure locations. Only persons who have signed the locally appropriate data protection training will have access to the password-protected computer where entered data is stored. After conclusion of the project, data will be removed from the computers and stored in a safe place. Any scientific publications or reports will not identify any patient by name or initials. When the research team reviews their notes, they are also bound by professional confidentiality. All study data will be stored in secure databases only accessible to study staff. Study sponsors and health authorities will be given controlled access for the purpose of audit when necessary.

15.4. Sample Confidentiality and Management

Aliquots of blood will be stored initially at the site clinical diagnostic laboratory. Subsequently, aliquots will be shipped to national and/or international partner laboratories according to national and international regulations. Samples will be anonymised with a unique study identifier prior to shipping and any patient identifiable data will have been removed. The Country Principal Investigator and Chief Investigator will have access to the enrolment log linking study identifiers with patient identifiers. Material transfer agreements and export/import licenses (where required) will be obtained, and international regulations on storage and shipping of hazardous samples will be followed. Sample custody will be maintained by the investigators and decisions regarding use and transfer of samples will be made by the TSC. Research samples will be stored indefinitely and approval from the sponsor and/or ethics committees, as appropriate, will be sought prior to destruction. The study investigators are responsible for biological deactivation and ensuring appropriate destruction of any residual materials.

15.5. Risks and Benefits

There are no approved treatments for EVD and the standard of care remains supportive care and treatment of complications. An analysis of data on 3343 confirmed and 667 probable EVD cases estimates a case fatality rate of 70.8% (95% confidence interval [CI], 69 to 73).[2] Unpublished data from MSF indicates that the survival rate in laboratory confirmed cased of Ebolavirus infection admitted to Gueckedou Ebola Treatment Centre is not likely to be more than 50% overall (see statistics section).

The potential efficacy, compared with safety and tolerability of the trial drug, is outlined in section 5.2. Potential AEs that require increased clinical surveillance are outlined in 8.2.

Of note, the potential teratogenicity of TKM is unknown. To date, the fatality in foetus' of pregnant women with EVD is not known to be less than 100%. Pregnant women will not be included in the TKM trial initially. This will be reviewed by the IDMC following enrolment and treatment of the first 15 patients.

TKM has never been used in children. In the initial stage of the study eligibility to the TKM arm will be restricted to patients 18 years of age or older until the first 15 patients are enrolled and treated. At that time all available, relevant safety data will be reviewed by the IDMC and a recommendation may be made to extend the eligibility to patients 5 years or older or to some other intermediate age between 5 to 18 years, as recommended by the IDMC.

Potential provision to younger patients, children under the age of 5, will be discussed with an expert panel.

The risks of conducting this trial include the possibility that the operation of the Ebola Treatment Centre (ETC) where the trial is conducted is compromised by any of the following events: a.) the ETC is overwhelmed by suspected EVD cases referred or self-referring in the belief that an effective treatment is available at the ETC; b.) the ETC security is breached by persons seeking access to the investigation product; c.) the security and safety of the ETC and ETC staff are compromised if there is a perception that the investigational product or the clinical trial are harmful to individual or community interests.

Whilst these are significant risks they are not in themselves arguments for not conducting this trial, since these risks will exist for all ETCs operating in this setting, the risks may be most acute for the earliest trials conducted.

Mitigation of these risks will be achieved by the following activities: A) Community sensitization and participation in the run up to trial initiation. Experienced health promotion and community outreach workers will conduct this activity. B) Study drug will be stored in a locked metal cabinet within a secure building. C) Contingency plans for handling an increase in the number of suspected cases will be developed, including strict criteria for closing to new admissions at the site conducting the study and the identification of satellite ETC centers to accept patients that cannot be acceptable at the ETC conducting the trial. D) Evacuation plans will be in place in the event of a major security breach.

15.6. Expenses

Clinic and treatment costs are covered by the ETC funding organisation (and partner laboratories) for all patients presenting to the Ebola Treatment Centre regardless of study participation. The study drug will be supplied without cost to the patient. Patients will be issued a mobile phone for the purposes of follow-up. This phone and SIM card will become the property of the patient. Reasonable travel expenses and compensation for loss of earnings will be paid for attending follow-up at the Ebola Treatment Centre. No other funding or incentives for participation will be given to the patients.

15.7. Contemporaneous Studies

Patients cannot enroll to any other clinical trial that involves a therapeutic or care intervention while they are participating in this study. If participating sites are undertaking observational studies, patients may be co-enrolled provided the combined study procedures are considered safe and appropriate for the patients and there is no conflict of outcomes or endpoints between the studies.

15.8. Alternatives to Study Participation

All patients will be treated with the best available care regardless of study participation. Patients are free to decline participation in this study without effect on the standard care provided.

15.9. Community Engagement

Standard interventions likely to be used by the managing organisation include dissemination of health promotion messages to health authorities and communities regarding ETC activities and information on EVD transmission, prevention, reporting and response. Information on this research study would be added to these messages.

Messages to the community in EVD regions are disseminated by networks of Health Promotion workers who engage with local leaders that serve as primary sources of information for the communities. Radio broadcasting and printed information sheets and posters are also common tools. This standard protocol on dissemination of critical messages would be used to inform the communities about research done at the Ebola Treatment Centre. Information regarding the nature and purpose of the research would be distributed with a variety of tools across these networks.

Education on the current development status of EVD treatments, clinical research studies ongoing in West Africa and the scientific background and methods of the current project would be distributed to and discussed with the health authorities already engaged as a part of the Health Promotion activities.

Most treatment centres also provide psychological and social support to EVD patients. When presenting to the Ebola Treatment Centre, patients and families will be approached by a team social worker to facilitate the psychological process throughout admission, EVD confirmation, treatment and when required, death and bereavement. Patients who are discharged from the Treatment Centre will be supported in their return to the community. This team will be engaged in the research study according to the requirements of the sites to assist with explaining the nature and methods of the research, obtain informed consent, and discuss the study with the family.

16. FINANCE AND INSURANCE

16.1. Funding

The trial is funded by the Wellcome Trust.

16.2. Insurance

The University of Oxford has arranged appropriate insurances to provide for the University's responsibilities, as Sponsor, to research subjects; and, to cover the legal liabilities of the University to those

engaged by the University in the performance of this research. The University will also arrange, or arrange in conjunction with other participating partners those emergency medical repatriation facilities which can be achieved, subject to the exigencies of arranging such at the material time.

17. PUBLICATION POLICY

Results from the trial will be published in open access and the data will be available for sharing.

18. REFERENCES

1. WHO. Ebola Response Roadmap Situation Report 1. 29 August 2014. http://www.who.int/csr/disease/ebola/situation-reports/29-august-2014.pdf?ua=1. Accessed 3rd September 2014.
2. WHO Ebola Response Team. Ebola virus disease in West Africa--the first 9 months of the epidemic and forward projections. *N Engl J Med.* 2014;371(16):1481-1495.
3. Kucharski AJ, Edmunds WJ. Case fatality rate for Ebola virus disease in west Africa. *Lancet.* 2014;384(9950):1260.
4. Gire SK, Goba A, Andersen KG, et al. Genomic surveillance elucidates Ebola virus origin and transmission during the 2014 outbreak. *Science.* 2014;345(6202):1369-1372.
5. Fowler RA, Fletcher T, Fischer WA, 2nd, et al. Caring for critically ill patients with ebola virus disease. Perspectives from west Africa. *Am J Respir Crit Care Med.* 2014;190(7):733-737.
6. Mahanty S, Bray M. Pathogenesis of filoviral haemorrhagic fevers. *Lancet Infect Dis.* 2004;4(8):487-498.
7. Takada A, Kawaoka Y. The pathogenesis of Ebola hemorrhagic fever. *Trends Microbiol.* 2001;9(10):506-511.
8. Bausch DG, Towner JS, Dowell SF, et al. Assessment of the risk of Ebola virus transmission from bodily fluids and fomites. *J Infect Dis.* 2007;196 Suppl 2:S142-147.
9. Towner JS, Rollin PE, Bausch DG, et al. Rapid diagnosis of Ebola hemorrhagic fever by reverse transcription-PCR in an outbreak setting and assessment of patient viral load as a predictor of outcome. *J Virol.* 2004;78(8):4330-4341.
10. Ebihara H, Rockx B, Marzi A, et al. Host response dynamics following lethal infection of rhesus macaques with Zaire ebolavirus. *J Infect Dis.* 2011;204 Suppl 3:S991-999.
11. Rollin PE, Bausch DG, Sanchez A. Blood chemistry measurements and D-Dimer levels associated with fatal and nonfatal outcomes in humans infected with Sudan Ebola virus. *J Infect Dis.* 2007;196 Suppl 2:S364-371.
12. Wauquier N, Becquart P, Padilla C, et al. Human fatal zaire ebola virus infection is associated with an aberrant innate immunity and with massive lymphocyte apoptosis. *PLoS Negl Trop Dis.* 2010;4(10).
13. Oestereich L, Ludtke A, Wurr S, et al. Successful treatment of advanced Ebola virus infection with T-705 (favipiravir) in a small animal model. *Antiviral Res.* 2014;105:17-21.
14. Qiu X, Wong G, Audet J, et al. Reversion of advanced Ebola virus disease in nonhuman primates with ZMapp. *Nature.* 2014.
15. Warren TK, Wells J, Panchal RG, et al. Protection against filovirus diseases by a novel broad-spectrum nucleoside analogue BCX4430. *Nature.* 2014;508(7496):402-405.
16. Sayburn A. WHO gives go ahead for experimental treatments to be used in Ebola outbreak. *BMJ.* 2014;349:g5161.
17. Geisbert TW, Lee AC, Robbins M, et al. Postexposure protection of non-human primates against a lethal Ebola virus challenge with RNA interference: a proof-of-concept study. *Lancet.* 2010;375(9729):1896-1905.
18. Geisbert TW, Hensley LE, Kagan E, et al. Postexposure protection of guinea pigs against a lethal ebola virus challenge is conferred by RNA interference. *J Infect Dis.* 2006;193(12):1650-1657.
19. Jovic G, Whitehead J. An exact method for analysis following a two-stage phase II cancer clinical trial. *Stat Med.* 2010;29(30):3118-3125.
20. Clopper CJ, Pearson ES. The use of confidence or fiducial limits illustrated in the case of the binomial. *Biometrika.* 1934;26:404-413.
21. Bross IJ, Delaney MM. Significance tests for certain 2 x 2 tables. *Am J Hyg.* 1952;55(3):357-362.
22. Whitehead J. *The Design and Analysis of Sequential Clinical Trials*. Revised 2nd edition ed. Chichester: Wiley; 1997.

23. Whitehead J, Todd S. The double triangular test in practice. *Pharmaceutical Statistics.* 2004;3(1):39-49.

24. Fairbanks K, Madsen R. P values for tests using a repeated significance test design. *Biometrika.* 1982;69:69-74.

25. WHO. *Ethical considerations for use of unregistered interventions for Ebola viral disease. Report of an advisory panel to WHO.* [Online]. 2014. Available from: http://apps.who.int/iris/bitstream/10665/130997/1/WHO_HIS_KER_GHE_14.1_eng.pdf?ua=1. [Accessed 9th September 2014].

Trial Title: Rapid Assessment of Potential Interventions & Drugs for Ebola (RAPIDE) – TKM

Scientific Title: Open-label, single arm trial to investigate the efficacy of TKM-130803 with a concurrent observational study of Ebolavirus Disease in Sierra Leone.

Short title: Evaluation of TKM Treatment for Ebolavirus Disease

Date and Version No: 19 March, 2015 v 2.8

Chief Investigator: **Peter Horby**
Centre for Tropical Medicine and Global Health
Nuffield Department of Medicine Research Building
University of Oxford
Old Road Campus, Roosevelt Drive
OXFORD, OX3 7FZ

Country Principal Investigator: **Foday Sahr** 34 Military Hospital, Ministry of Health and Sanitation Freetown, Sierra Leone

Co-Investigators: **James Russell**, Sierra Leone College of Medicine and Allied Health Sciences

G.F. Deen, Sierra Leone College of Medicine and Allied Health Sciences

Stephan Gunther, Bernard Nocht Institute for Tropical Medicine

Tim Brooks, Rare and Imported Pathogens Laboratory, Public Health England

Trudie Lang Global Health Network, Centre for Tropical Medicine and Global Health, University of Oxford

Piero L Olliaro Newton-Abraham Visiting Professor, University of Oxford.

Jake Dunning (Field Clinical Lead) Centre for Tropical Medicine and Global Health, University of Oxford

John Whitehead Department of Mathematics and Statistics, Lancaster University

Sponsor: University of Oxford

Joint Research Office, Block 60, Churchill Hospital, Oxford, OX4 7LE

Funder: Wellcome Trust

PARCT: PACTR201501000997429

TABLE OF CONTENTS

1. KEY TRIAL CONTACTS 4
2. SYNOPSIS 6
3. ABBREVIATIONS 8
4. INTRODUCTION 9
5. DRUG INFORMATION 10
5.1. Efficacy data against EBOV 10
5.2. Safety and tolerability 10
5.3. Dosing and dosing rationale 11
6. OBJECTIVES AND OUTCOME MEASURES 12
7. TRIAL DESIGN 12
7.1. Trial design 12
7.2. Trial design rationale 13
7.3. Eligibility for TKM 13
7.4. Trial enrolment 13
7.5. Participant inclusion criteria 15
8. TRIAL PROCEDURES 16
8.1. Screening and informed Consent 16
8.2. Procedures for provision of TKM 17
8.3. Laboratory Samples 18
8.4. Procedures for standard care 18
8.5. Treatment Interruption 18
8.6. Discontinuation of Trial Treatment 18
8.7. Withdrawal from the Study 19
9. DATA COLLECTION 19
9.1. Clinical data acquisition 19
9.2. Follow up Assessments (D14, D30, Months 3, 6, 12) 20
9.3. Laboratory Assessments 21
10. TRIAL DRUGS 22
10.1. Formulation 22
10.2. Storage and Accountability 23
11. SAFETY REPORTING 23
11.1. Definitions for safety reporting 23
11.2. Procedures for safety reporting 24

12. STATISTICS 24
12.1. Description of Statistical Methods 24
12.2. The Number of Participants 25
12.3. Criteria for the Termination of the Trial 26
12.4. Final Analysis 27
12.5. Inclusion in Analysis 27
12.6. Procedure for Accounting for Missing, Unused, and Spurious Data. 27
12.7. Rationale for statistical design choice 27
13. DATA MANAGEMENT 28
13.1. Data Capture 28
13.2. Access to Data 28
14. QUALITY ASSURANCE PROCEDURES 28
15. ETHICAL AND REGULATORY CONSIDERATIONS 29
15.1. Regulations, Guidelines and Approval 29
15.2. Considerations in Informed Consent 29
15.3. Participant Confidentiality 31
15.4. Sample Confidentiality and Management 31
15.5. Risks and Benefits 32
15.6. Expenses 33
15.7. Contemporaneous Studies 33
15.8. Alternatives to Study Participation 33
15.9. Community Engagement 33
16. FINANCE AND INSURANCE 34
16.1. Funding 34
16.2. Insurance 34
17. PUBLICATION POLICY 34
18. REFERENCES 35

1. KEY TRIAL CONTACTS

**Chief Investigator**	Associate Professor Peter Horby<br><br>Centre for Tropical Medicine and Global Health<br>Nuffield Department of Medicine Research Building<br>University of Oxford<br>Old Road Campus, Roosevelt Drive<br>OXFORD<br>OX3 7FZ<br><br>Tel: 07990 560237<br>Email: Peter.horby@ndm.ox.ac.uk
**Sponsor**	University of Oxford<br>Joint Research Office<br>Block 60, Churchill Hospital<br>Oxford, OX4 7LE
**Statistician**	Professor John Whitehead<br><br>Department of Mathematics and Statistics<br>Fylde College<br>Lancaster University<br>Lancaster LA1 4YF<br><br>Tel: 01524 389967<br>Email: j.whitehead@lancaster.ac.uk
**Committees**	**Trial Steering Committee Voting Members:**<br><br>**Samuel Kargbo**<br>Ministry of Health and Sanitation, Freetown, Sierra Leone<br><br>**Mohamed Samai,**<br>College of Medicine and Allied Health Sciences, Freetown, Sierra Leone<br><br>**Foday Sahr**<br>34 Military Hospital, Ministry of Health and Sanitation Freetown, Sierra Leone<br><br>**Stephen Kennedy**<br>Pacific Institute for Research and Evaluation, Monrovia, Liberia<br><br>**Fred Binka** (Independent Member)<br>University of Health and Allied Sciences, Ho, Ghana<br><br>**Peter Horby** (Chief Investigator)<br>Centre for Tropical Medicine and Global Health, Oxford, United Kingdom<br>International Severe Acute Respiratory and emerging Infections Consortium

	**John Whitehead** (Trial Statistician)<br>Lancaster University, Lancaster, United Kingdom<br><br>**Rob Fowler** (Independent Member)<br>Sunnybrook Health Sciences Centre, Toronto, Canada<br><br>**Nicholas White** (Independent Member - Chairperson)<br>Mahidol-Oxford Research Unit, Bangkok, Thailand<br><br>**Fiona Gannon**<br>GOAL Programme Quality Advisor, Republic of Ireland<br><br>**Non-Voting Members**<br>**Ana Maria Henao-Restrepo** (WHO Representative)<br>World Health Organization, Geneva, Switzerland<br><br>**Independent Data Monitoring Committee (IDMC) Members:**<br><br>**David Lalloo** (Chair), Professor of Tropical Medicine; Dean of Clinical Sciences and International Public Health, Liverpool School of Tropical Medicine<br><br>**Clement Adebamowo,** Director Office of Strategic Information and Research Institute of Human Virology in Nigeria<br><br>**Donald Berry,** Founder and senior Statistical Scientist, Berry Consulting<br><br>**Nicolas Opoku,** Director Onchocerciasis Chemotherapy Research Centre, Hohoe, Ghana<br><br>**Peter Smith,** Professor of Tropical Epidemiology, London School of Hygiene and Tropical Medicine<br><br>**Durodami Radcliffe Lisk,** Professor Consultant Neurologist, Ministry of Health Coordinator, Epilepsy Association of Sierra Leone, College of Medical and Applied Health (COMAH), Sierra Leone

2. SYNOPSIS

Trial Title	**Rapid Assessment of Potential Interventions & Drugs for Ebola (RAPIDE) – TKM**	
Scientific Title	Open-label, single arm trial to investigate the efficacy of TKM-130803 with a concurrent observational study of Ebolavirus Disease in an outbreak setting in West Africa	
Internal ref. no. (or short title)	Evaluation of TKM-130803 Treatment for Ebolavirus Disease	
Clinical Phase	2	
Trial Design	Open-label, single arm trial, with a concurrent observational study	
Trial Participants	Patients with confirmed Ebolavirus disease attending the participating treatment centre	
Planned Sample Size	Up to 100 adult patients (with possible late inclusion of paediatric patients)	
Treatment duration	1 week	
Follow up duration	12 months follow up	
	Objectives	Outcome Measures
Primary	<u>TKM:</u> To evaluate the impact of TKM treatment on early mortality in EVD<br><br><u>Observational:</u> To characterize the early mortality of EVD in an ETC	Mortality at Day (D)14 (in those that survive first 48 hours after admission)<br><br>Mortality at D14 (in those that survive first 48 hours after admission)
Secondary	<u>TKM</u><br>1. To evaluate the impact of TKM-130803 treatment for adults on:<br>a) Overall morality at D14<br>b) Time to recovery<br>c) Late mortality<br>d) Viral load<br>e) EVD symptoms<br>f) EVD antibody response<br>g) Long term clinical recovery<br><br>2. i)To assess the safety of TKM treatment for adults.<br>ii) To measure the pharmacokinetics (PK) of TKM following repeat dosing	<u>TKM</u><br>1. a) D14 mortality in all patients allocated to TKM-130803 treatment (not excluding deaths in first 48 hours).<br>b) Time to meeting ETC discharge criteria.<br>c) Mortality at D30 and months 3,6,12 after first dose of study treatment<br>d) Viral load<br>e) Presence and duration of symptoms (SDs 1-14)<br>f) Convalescent anti-Ebolavirus IgG titer (D30)<br>g) Clinical assessment at months 3,6,12.<br><br>2. Incidence of SARs, key adverse events (SDs 1-14) and monitoring of vital signs (pulse rate, blood pressure, respiratory rate, temperature) pre, during and at 0, 1, 2, 4 and 8 hours post end of infusion<br>ii) PK pre-dose and at the end of infusion on SDs 1, 3, 5 and 7

	<u>Observational</u><br>To characterise the natural history of EVD in an ETC.<br>a) Time to recovery<br>b) Late mortality<br>c) Viral load<br>d) EVD symptoms<br>e) EVD antibody response<br>f) Long term clinical recovery	<u>Observational</u><br>a) Time to meeting ETC discharge criteria.<br>b) Mortality at D30 and months 3,6,12 after admission<br>c) Viral load<br>d) Presence and duration of symptoms (SDs 1-14)<br>e) Convalescent anti-Ebolavirus IgG titer (D30)<br>f) Clinical assessment at months 3,6,12.
Investigational Medicinal Product	TKM-130803 (TKM)	
Formulation, Dose, Route of Administration	TKM is provided as 0.3 mg/kg, administered as a 2-hour intravenous infusion (IV), in a final volume of 150ml, once daily for seven days.<br><br>To assess the feasibility and safety of dosing with TKM-130803 in an ETU, data on the first four enrolled patients (the safety cohort) will be assessed by the IDMC prior to opening enrollment to additional patients. The safety cohort may be expanded following advice from the IDMC.<br><br>In the event of suspected drug-related toxicity or a change in the patient's clinical condition, the dose of TKM-130803 may be reduced to a minimum of 0.24 mg/kg once daily at the discretion of the treating physician. TKM will not be given to children or pregnant women during the initial phase of the study.<br><br>Administration of the drug will adhere to the relevant trial Standard Operating Procedure (SOP) document.	

3. ABBREVIATIONS

AE	Adverse event
CI	Confidence Interval
Coags	Coagulation studies
CRF	Case Report Form
D	Days (since admission)
EBOV	Ebolavirus
EDTA	Ethylenediaminetetraacetic acid
ETC	Ebola Treatment Centre
EVD	Ebolavirus Disease
FBC	Full blood count
GCP	Good Clinical Practice
IB	Investigators Brochure
ICF	Informed Consent Form
ICH	International Conference on Harmonisation
IDMC	Independent Data Monitoring Committee
IMP	Investigational Medicinal Product
ITT	Intention to Treat
IV	Intravenous
LFT	Liver function tests
MAD	Multiple Ascending Dose
MSF	Medecins Sans Frontieres
OXTREC	Oxford Tropical Research Ethics Committee
PI	Principal Investigator
PK	Pharmacokinetic
REC	Research Ethics Committee
RNA	Ribonucleic acid
RT-PCR	Reverse transcription polymerase chain reaction
SAD	Single Ascending Dose
SAE	Serious Adverse Event
SAR	Serious Adverse Reaction
SD	Study days
SOP	Standard Operating Procedure
SUSAR	Suspected Unexpected Serious Adverse Reactions
TKM	TKM-130803
TSC	Trial Steering Committee
UEC	Urea, Electrolytes, Creatinine
WHO	World Health Organisation

4. INTRODUCTION

The size and scale of the on-going Ebola Virus Disease (EVD) outbreak is unprecedented, and has been declared a Public Health Emergency of International Concern.[1] EVD is among the most virulent infectious agents known: an analysis of data on 3343 confirmed and 667 probable EVD cases collected during the current outbreak in Guinea, Liberia, Nigeria, and Sierra Leone estimates a case fatality rate of 70.8% (95% confidence interval [CI], 69 to 73) among persons with known clinical outcome of infection.[2] A figure that is consistent with estimates of other authors.[3] Figure 1 shows the key time points in the clinical course of EVD in this outbreak.[2]

Figure 1. Estimated mean (median) time course in days of Ebolavirus infection in this outbreak[2]

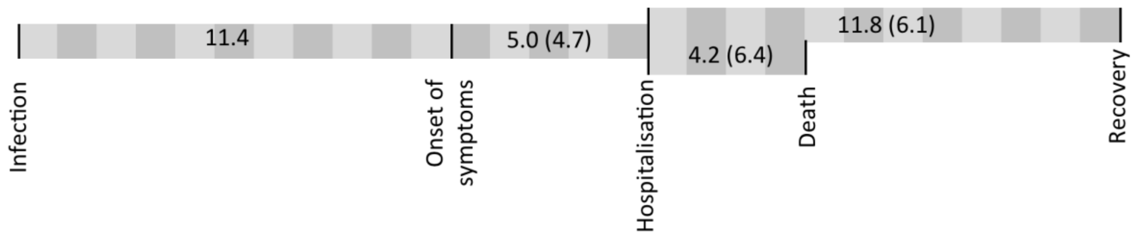

The current Ebolavirus strain causing the outbreak in West Africa is related to the Ebola Zaire strain and genetic analysis suggests the current outbreaks in Guinea, Sierra Leone and Liberia are all related to a single transmission event from a zoonotic source followed by human-to-human transmission.[4] EVD is characterized by a febrile illness dominated by fatigue and gastrointestinal symptoms that can be complicated by shock, haemorrhage and multi-organ failure. However, clinical presentation and severity is variable, with some patients remaining relatively well, able to ambulate and self-feed throughout their illness, whilst others progress rapidly to a fatal outcome.[2,5]

The pathogenesis of EVD is incompletely understood[6,7] but high levels of viral replication and the detection of virus in multiple body tissues is typical of severe disease.[8,9] Coagulopathy, disruption of endothelial function and increased inflammatory responses are also associated with severe EVD.[6,10-12] The association between high levels of viraemia and EVD severity suggests that therapies that target viral replication may benefit patients. Several experimental products that target Ebolavirus replication have shown some efficacy in animal studies.[13-15] However, whilst several therapeutic interventions have shown promise in the laboratory and in animal studies, none have been tested for efficacy and safety in humans with EVD. A World Health Organization (WHO) expert panel recently concluded unanimously that "investigators have a moral duty to evaluate these interventions in the best possible clinical studies that can be conducted under the circumstances of the epidemic."[16]

The aim of this protocol is to assess the effectiveness of the drug TKM-130803 (produced as TKM-130803 by Tekmira Pharmaceuticals) for the treatment of EVD.

The Trial Steering Committee selected TKM as one of the leading drugs suitable for urgent evaluation from a possible list of 10 candidate therapies. The criteria for selection were based upon a Target Product Profile that considered existing clinical and pre-clinical data supporting safety and efficacy, immediate availability and scalability, and an acceptable treatment regimen.

The purpose of this trial is to establish whether TKM is a promising treatment for Ebola Virus Disease.

5. DRUG INFORMATION

TKM-130803 is a drug that has been developed specifically to target EBOV. TKM-130803 is a lipid nanoparticle formulation of 2 small interfering RNAs (siRNAs) directed against Ebolavirus L polymerase and Viral Protein-35 (VP-35). These two proteins are involved in transcription and replication of EBOV. TKM-130803 is a new formulation of drug TKM-100802 (formerly TKM-100201), which has been adapted to improve its specificity to the Guinea variant viral strain causing the current outbreak by a two nucleotide swap in the VP35 siRNA and a single nucleotide swap in the L-pol siRNA to ensure complete match to the emergent strain. TKM-100201 and TKM 100802 have been evaluated in guinea pig, non-human primate, and human Phase 1 clinical trials. TKM-130803 treatment of Rhesus monkeys at 72 hours following challenge with a lethal dose of Ebola virus Guinea 2014 led to 67% survival (2/3 animals). TKM-100802 drug has received authorization for emergency use in EVD patients from the US Food and Drug Administration. 100 treatment courses of TKM-130803 are available for this trial, in a wet (non-lyophilized) formulation.

5.1. Efficacy data against EBOV

In-vitro activity of TKM-100802 was established using two variants of EBOV infecting a human hepatoma cell line. The EC50 ranged between <0.003nM and 0.04nM. The therapeutic index was determined to be between 1513 and >18954 for treatment commencing one hour following viral inoculation. Molecular analysis confirmed the proposed mechanism of activity of the two siRNAs. In-vivo trials have established that TKM-100802 has an inhibitory impact on virus replication in EBOV-infected cells, as well as a survival benefit in EBOV-infected guinea pigs and rhesus macaques[17,18]. In rhesus monkeys infected with a lethal challenge of Zaire EBOV, 100% survival has been observed in animals that received TKM-100802 at 0.5 mg/kg/day for 7 days, with 67% survival at 0.2 mg/kg/day for 7 days. In rhesus monkeys infected with a lethal challenge of Guinea variant EBOV at 0.5 mg/kg/day for 7 days, TKM-130803 (siEbola-3 with LNP1) resulted in 33% survival (1/3) with treatment commencing at 48 hours post inoculation, and 67% (2/3) with treatment commencing at 72 hours post inoculation.

5.2. Safety and tolerability

TKM-130803 has the same formulation as TKM-100802 except for two nucleotide swaps in the VP35 siRNA and a single nucleotide swap in the L-pol siRNA. Therefore the safety and tolerability profile is expected to be similar to TKM-100802. For a detailed description of the safety and tolerability of TKM-100802, please refer to the Investigator Brochure and additional safety summary. Adverse events (AEs) considered most likely to occur with TKM-130803 include discomfort, bruising, and bleeding at the site of infusion, and adverse effects analogous to those observed in animal studies, including coagulopathies, renal impairment, hepatobiliary injury and acute phase reaction.

In a human single ascending dose trial of TKM-100201 administered over one hour to healthy participants, infusion-related reactions, notably cytokine release syndromes (CRS) were observed. Clinical manifestations included flushing, headache, fever, hypotension, chills, nausea, and vomiting. Most of the reported treatment related AEs were consistent with a transient inflammatory response that begins within 6 hours after infusion and dissipates in most cases by 24 hours post-dosing. This interpretation is consistent with laboratory findings of transient elevations in some cytokines (e.g., MCP-1, IL-6, IL-1ra, IL-8) in some patients measured 2 and 6 hours post-dosing, returning to at or near baseline levels by 24 hours. In

addition, focal and reversible pain has been observed in association with liposomal or LNP therapeutics, particularly in the back, sacrum and sternum.

Fertility and reproductive toxicity studies have not been performed with TKM-100802 or TKM-130803.

5.3. Dosing and dosing rationale

In rhesus monkeys infected with a lethal challenge of EBOV, 100% survival has been observed in animals that received TKM-100802 at 0.5 mg/kg/day for 7 days, with 67% survival at 0.2 mg/kg/day for 7 days. In rhesus monkeys infected with a lethal challenge of Guinea variant EBOV at 0.5 mg/kg/day for 7 days, TKM-130803 (siEbola-3 with LNP1) resulted in 33% survival (1/3) with treatment commencing at 48 hours post inoculation, and 67% (2/3) with treatment commencing at 72 hours post inoculation. Comparisons of drug exposure data from monkeys and healthy human volunteers indicate that plasma exposure parameters (C_{max} and AUC) are approximately equivalent between humans and monkeys when dosed at the same dose level of TKM-100802 (based on weight; mg/kg). Thus, it is anticipated that dose levels of at least 0.2 mg/kg/day will be required to meet exposure targets derived from efficacious dose levels currently used in monkey EBOV infection studies.

The no-observed-adverse-effect-level (NOAEL) of TKM-100802 in a repeat-dose toxicity study in cynomolgus monkeys was 0.25 mg/kg/day after daily 1-hour intravenous infusion of TKM-100802 for 14 days. The total cumulative dose (3.5 mg/kg) at the NOAEL is equivalent to the maximum cumulative dose proposed in humans (i.e., 0.5 mg/kg/day × 7 days; 3.5 mg/kg). The highest dose level (1.0 mg/kg/day for 14 days) was well tolerated in monkeys, but resulted in microscopic findings in the liver, spleen, and kidneys, which were not severe, but precluded consideration of this dose level as a NOAEL.

The safety and pharmacokinetics of TKM-100802 have been investigated in a single ascending dose (SAD) phase of Study TKM-EBOV-002 (A Placebo-Controlled, Single-Blind, Single Ascending Dose Study With Additional Multiple Ascending Dose Cohorts To Evaluate The Safety, Tolerability, and Pharmacokinetics of TKM-100802 In Healthy Human Volunteers) over a dose range from 0.075 mg/kg to 0.5 mg/kg. The maximum tolerated dose (MTD) was determined to be 0.3 mg/kg, using a conservative toxicity grading scale for healthy volunteers.

TKM-130803 will be administered by IV infusion at a rate of 1.25 mL/min, administered over approximately 120 minutes for a total volume of 150mls administered. For infusion related adverse reactions, the infusion rate may be slowed or interrupted at the discretion of the treating physician. If the infusion is interrupted and resumed, the total infusion should be completed within 4 hours of infusate preparation. The patient will be in recumbent position for dose administration. TKM will be administered via a dedicated peripheral IV or central line.

For full details of infusion preparation and administration please refer to the relevant trial Standard Operating Procedure manual.

6. OBJECTIVES AND OUTCOME MEASURES

	Objectives	Outcome Measures
Primary	TKM: To evaluate the impact of TKM treatment on early mortality in EVD<br><br>Observational: To characterize the early mortality of EVD in an ETC	Mortality at D14 (in those that survive first 48 hours after admission)<br><br>Mortality at D14 (in those that survive first 48 hours after admission)
Secondary	TKM<br>1. To evaluate the impact of TKM-130803 treatment for adults on:<br>a) Overall morality at D14<br>b) Time to recovery<br>c) Late mortality<br>d) Viral load<br>e) EVD symptoms<br>f) EVD antibody response<br>g) Long term clinical recovery<br><br>2. i)To assess the safety of TKM treatment for adults.<br>ii) To measure the pharmacokinetics (PK) of TKM following repeat dosing<br><br>Observational<br>To characterise the natural history of EVD in an ETC.<br>a) Time to recovery<br>b) Late mortality<br>c) Viral load<br>d) EVD symptoms<br>e) EVD antibody response<br>f) Long term clinical recovery	TKM<br>1. a) D14 mortality in all patients allocated to TKM-130803 treatment (not excluding deaths in first 48 hours).<br>b) Time to meeting ETC discharge criteria.<br>c) Mortality at D30 and months 3,6,12 after first dose of study treatment<br>d) Viral load<br>e) Presence and duration of symptoms (SDs 1-14)<br>f) Convalescent anti-Ebolavirus IgG titer (D30)<br>g) Clinical assessment at months 3,6,12.<br><br>2. Incidence of SARs, key adverse events (SDs 1-14) and monitoring of vital signs (pulse rate, blood pressure, respiratory rate, temperature) pre, during and at 0, 1, 2, 4 and 8 hours post end of infusion<br>ii) PK pre-dose and at the end of infusion on SDs 1, 3, 5 and 7<br><br>Observational<br>a) Time to meeting ETC discharge criteria.<br>b) Mortality at D30 and months 3,6,12 after admission<br>c) Viral load<br>d) Presence and duration of symptoms (SDs 1-14)<br>e) Convalescent anti-Ebolavirus IgG titer (D30)<br>f) Clinical assessment at months 3,6,12.

Note that there are two counting systems for days in the trial: days from admission (prefixed with D) and study days (prefixed with SD) from time of first data collection (see section 9.1).

7. TRIAL DESIGN

7.1. Trial design

This research consists of a single-arm, open-label trial of TKM and a concurrent observational trial.

Ongoing observation of the clinical manifestations and outcomes of patients in ETCs is valuable, especially when data acquisition is undertaken in a systematic manner as part of trial.

7.2. Trial design rationale

Due to production capacity limits there are currently only 100 doses of TKM-130803 available for this trial.

The risk of infusion reactions means that TKM-130803 must be infused over a minimum 2-hour period during which clinical monitoring for infusion reactions is necessary. The intensity of clinical monitoring required means that infusions will, in most cases, only be undertaken in the morning (when the day shift of clinical staff and the trial staff are all on site). Even then, the challenges of clinical care delivery within an ETC means that the number of participants who can receive TKM-130803 infusions and be safely monitored on any one day may be limited by staffing availability. ETCs may also vary in their capacity to administer TKM-130803 based on fluctuating patient-staff ratios, and inpatient numbers. Therefore, the maximum number of patients receiving TKM-130803 on any one day will need to be capped. The lead physician at the ETC will notify the maximum number of monitored beds available for the trial on any given day. Once the bed availability is known, if the number of patients eligible for TKM-130803 exceeds capacity they will be allocated to receive either the drug with standard care (as part of an interventional group) or standard care (as part of an observational cohort). The methodology for allocating cohorts is described in section 7.3.

7.3. Eligibility for TKM

Since TKM-130803 has never been used in children and the bio-distribution and pharmacokinetics is not known in this population, in the initial stage of the study, eligibility to the TKM arm will be restricted to patients 18 years of age or older until the first 15 patients have been enrolled and treated. At that time, all available and relevant safety data will be reviewed by the IDMC and a recommendation may be made to extend the eligibility to patients 5 years or older or to some other intermediate age between 5 to 18 years.

Similarly, there have been no fertility or reproductive toxicity studies on TKM – 130803. Hence pregnant and breastfeeding women will also be initially excluded from the trial. After the first 15 patients have been enrolled and treated, the data will be reviewed by the TSC and the TSC may recommend to include pregnant women or continue to exclude them from this trial. If the TSC recommend inclusion of pregnant women, Ethical Review Board approval will be sought.

TKM is administered as an infusion. If reliable IV access cannot be obtained for a patient, they will not be eligible to receive the trial drug nor take part in the observational cohort.

7.4. Trial enrolment

Trial enrolment and allocation to treatment or observational cohort will take place as described in Figure 2 and below.

1. All ETC patients with a laboratory confirmed case of EBOV who can tolerate trial procedures, do not have organ failure and in whom IV access is possible will be eligible for enrolment into the observational trial.
2. Patients will also be assessed for eligibility for drug allocation according to the criteria in section 7.3.
3. Allocation into this drug eligible cohort will occur by the following process.

i. Following arrival of the trial staff each morning, the maximal number of study beds (b) available for patients to receive study drug will be determined by the ETC clinical lead and the trial clinical lead.

    ii. Whenever possible, patient dosing will be started within 2 hours of the trial team arrival in the morning, in order to provide adequate time for patient monitoring and to complete the required assessments during the working day. Consent and any randomisation will need to be completed before this time. If a patient chooses to delay consent, or consent cannot be obtained e.g. family or proxy not available, consent and randomisation of other patients will proceed. Any patients who cannot consent will be considered for enrolment the following day if this is still within the required 48 hour window, see Section 8.1.

iii. Following consent of patients who have arrived with or received PCR confirmation in the preceding 48hrs, each patient eligible for TKM-130803 treatment will be randomly allocated a number (n) from 1 to N, where N is the number of patients eligible (using a random number allocation program code in R statistical software).

iv. Patients will be allocated to the treatment cohort by sequentially selecting patients by their number (n) from 1 to b. The remainder of patients will remain in the observational cohort (when N>b).

v. If on the calendar day of randomisation a patient allocated to TKM-130803 treatment is unable to receive treatment (e.g. because they refuse, die, or IV access cannot be obtained), the next patient in the sequence may be entered in to the treatment arm. This may only occur on the calendar day of randomisation.

vi. Re-randomisation is not permitted.

Figure 2. Process of assessing eligibility and patient allocation to treatment or observational cohort

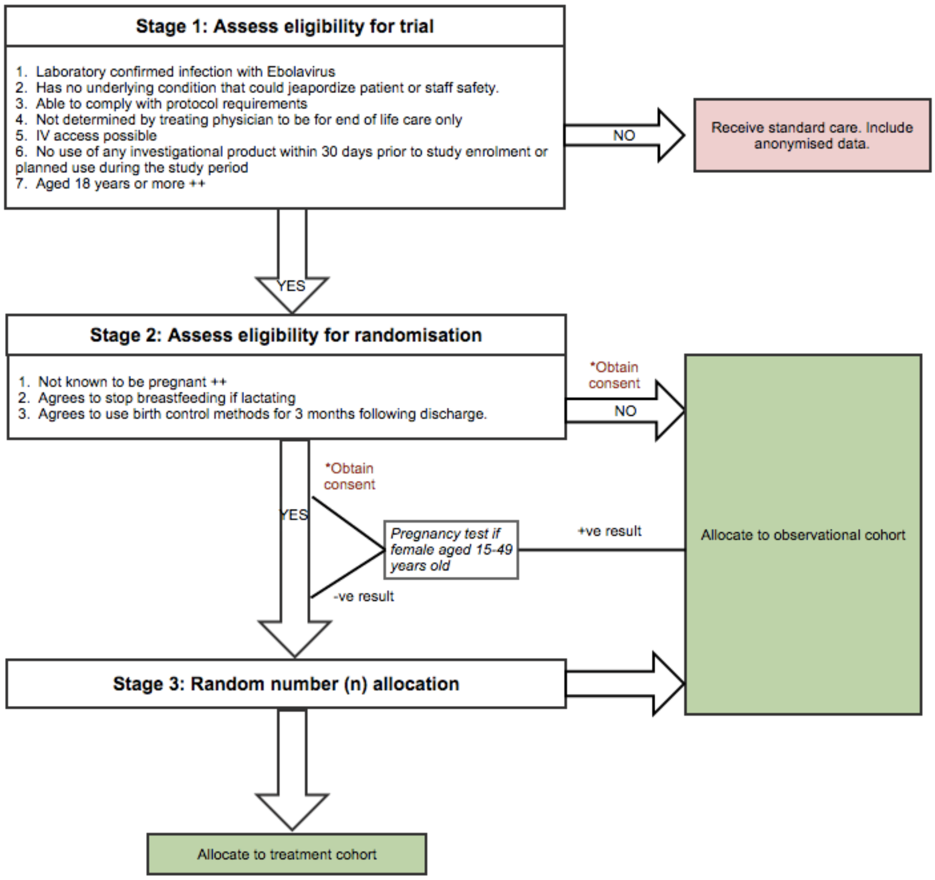

++ = criteria may change following review of initial findings

To assess the feasibility and safety of dosing with TKM-130803 in an ETU, data on the first four enrolled patients will be assessed by the IDMC prior to opening enrollment to additional patients. These patients (termed the safety cohort) may be recruited from one or multiple study sites. Each patient will receive at least 3 doses before dosing of the next patient starts. If a patient dies prior to receiving 3 doses, recruitment of the next patient may occur earlier. The IDMC will review data according to the schedule in Table 1 below. If the 4th patient dies prior to receiving 3 doses, the IDMC review may be brought forward. The safety cohort may be expanded following advice from the IDMC.

Table 1: Safety Cohort recruitment schematic.

													IDMC			
Patient 1	X	X	X	X	X	X	X									
Patient 2				X	X	X	X	X	X	X						
Patient 3							X	X	X	X	X	X	X			
Patient 4										X	X	X	X	X	X	X
Patient 5														X	X	X
Patient 6														X	X	X

**Patients 5 and 6 are recruited once recruitment is open following review by the IDMC.*

7.5. Participant inclusion criteria

The eligibility is a two-step process, first for the trial and then for the allocation to the drug eligible cohort. Patient ages <18 years will be excluded from both TKM treatment and observational cohorts until subsequent time as the IDMC recommended otherwise

Eligibility criteria for the trial (stage 1):

- Laboratory confirmed infection with Ebolavirus
- Adult aged ≥ 18 years (although the age range may be extended to >5years old following IDMC review of initial findings).
- Has no underlying condition that could jeopardize patient or staff safety
- Able to comply with protocol requirements
- Not determined by treating physician to be for end of life care only.
- IV access possible
- No use of any investigational or non-registered product within 30 days prior to study enrolment or planned use during the study period.

From within this cohort, additional eligibility criteria for randomization (stage 2):

- Not pregnant (although this might change after TSC review of the first 15 patients)
- Not breastfeeding (for lactating women)
- Agrees to use birth-control for 3 months post study.

The study of TKM treatment will concern the outcomes of all patients who are allocated to TKM treatment. For these patients, D1 will be the day of admission to the treatment centre. The primary response for the trial will be survival to D14 (yes or no). Patients who are allocated to TKM treatment and survive the first 48 hours after admission but do not actually receive it or who fail to complete the course of therapy, for any reason, will nevertheless be included in this primary analysis.

The observational study will include all patients who are admitted to the treatment centre during the period of the trial, including those considered eligible for TKM treatment but who are not allocated to it due to the limited number of available treatment beds. For the analysis of the outcomes of these patients, D1 will be the day of admission to the treatment centre.

Data from participants who for any reason are ineligible for the trial or refuse to participate will be included (anonymously) in the primary analysis of the observational group. All patient outcome data is routinely recorded in all ETCs and shared anonymously with WHO, therefore the use of anonymised D14 outcome data is in line with current practices.

8. TRIAL PROCEDURES

8.1. Screening and informed Consent

Patients with reverse transcription polymerase chain reaction (RT-PCR) confirmed Ebolavirus RNA (confirmed patients) will be screened for trial eligibility and invited to give informed consent to participate, according to the procedures outlined in section 15.2. Staff will not discuss informed consent with patients (or the parents/representatives of patients <18 years old) who are not eligible for enrolment in any trial

arm. The number of patients who are ineligible will be recorded anonymously. Study dosing, sampling and data collection will begin only after enrolment.

A patient may be consented to take part in the study anytime within 48 hours of first arriving at the ETC with a confirmed diagnosis, or, within 48 hours of being informed of Ebolavirus positive PCR result, if this occurs while already at the centre. Therefore, if a patient has been excluded for a reason that resolves within this period, they may be approached upon resolution of the reason for exclusion. If a patient wishes to delay their decision regarding consent, they may consent at any time within the 48 hour period.

Whenever possible, patient dosing will be started within 2 hours of the trial team arrival in the morning, in order to provide adequate time for patient monitoring and to complete the required post dose assessments. The actual time of dosing will depend on various factors, including number of patients on the study and staffing levels in the ETC. Consent and any randomisation will need to be completed before this time. If a patient chooses to delay consent, or consent cannot be obtained e.g. family or proxy not available, consent and randomisation of other patients will proceed. Any patients who cannot consent will be considered for enrolment the following day if this is still within the required 48 hour window.

A list of eligible patients will be produced each day (following receipt of the laboratory results).

Once randomised, re-randomisation is not permitted.

8.2. Procedures for provision of TKM

Patients randomized into the treatment cohort will receive TKM-130803 in addition to the supportive therapy provided to all patients.

TKM-130803 will be administered at a dose of 0.3mg/kg by IV infusion at a rate of **1.25 mL/min** administered over approximately 120 minutes in a total volume of 150ml. Infusions should be administered using an infusion pump, to ensure a well-controlled rate of infusion.

For full details of infusion preparation and administration please refer to the trial Standard Operating Procedure for drug administration.

The recruitment process for the safety cohort is described in Section 7.4. The IDMC will review data from the safety cohort prior to recruitment being opened to all eligible patients. The safety cohort may be expanded following advice from the IDMC.

Doses will be administered once daily, for seven days. In the event of suspected drug-related toxicity or a change in the patient's clinical condition, the dose of TKM-130803 may be reduced to a minimum of **0.24 mg/kg** once daily at the discretion of the treating physician. If the infusion is interrupted and resumed, the total infusion should be completed within 4 hours of infusate preparation. The patient will be in recumbent position for dose administration.

Standard safety assessments of patients will include monitoring of vital signs (pulse rate, blood pressure, respiratory rate, temperature), symptoms and SARs. Vital signs will be assessed at the following approximate time points: pre-infusion, during the infusion (preferably between 30-90 minutes), at the end of infusion, and at 1, 2, 4 and 8 hours post end of infusion, as well as at additional time points if indicated by the patient's clinical condition.

There are two potential adverse outcomes that require close clinical supervision.

1. Infusion related reactions may occur following commencement of the infusion. As the drug is given once a day, the timing of administration will be set to coincide with the presence of the trial staff in the treatment area. This will allow monitoring of symptoms and a decision to slow or halt the infusion if necessary. Pre-medication such as paracetamol may be given. IM adrenalin will also be made available for the event of an anaphylactic reaction.

2. Cytokine release syndrome may occur several hours after the infusion commences. Health care workers involved in care of patients receiving TKM will receive additional training to recognise the constellation of symptoms that are associated with it (flushing, headache, fever, hypotension, chills, nausea and vomiting). Management of a cytokine related event will prioritise immediate care (i.e. stopping of infusion (if ongoing), provision of IV fluids) and prompt medical review of the infusion speed and dose. Additional drugs (including steroids) may be given to ameliorate symptoms, at the treating physician's discretion. Procedures for safety reporting are outlined in 11.2.

8.3. Laboratory Samples

All patients, both in the observational and TKM cohort, will have regular blood samples drawn and analysis performed; see Section 9.2 Laboratory Assessments. Also, any residual volumes will be stored. Those in the observational cohort will have all the laboratory tests as for those in the TKM cohort; malaria, pregnancy (for women of childbearing age), ebola testing, electrolyte, renal and hepatic function testing. However, they will not have PK samples taken; these will only be taken from patients receiving the trial drug.

8.4. Procedures for standard care

The supportive therapy received by patients will not be affected by participation, or non-participation, in the trial. All patients will receive standard supportive therapy and this will be recorded. Treatment provided by clinical staff is based on the ETC guidelines for management of EVD. This includes oral and/or intravenous fluid resuscitation, empirical antibiotics, antimalarial agents, and also symptom control, which includes antiemetics, antipyretics/analgesics, and anti-diarrhoeal agents, as appropriate to the patient's symptoms.

8.5. Treatment Interruption

In the event of a SUSAR or other significant treatment related event, the treating physicians will determine if study treatment will continue on the basis of clinical picture and severity and resolution of the event. Consultation with the Independent Data Monitoring Committee will be made in the case of uncertainty to determine if the treatment should be discontinued.

If there is an indication of a cytokine response in the safety cohort (first 4 patients) then recruitment will be reviewed. The IDMC will review data from the complete cohort prior to recruitment being opened to all suitable patients. The safety cohort may be expanded following advice from the IDMC.

8.6. Discontinuation of Trial Treatment

Each participant has the right to discontinue use of study treatment at any time. In addition, the Investigator may discontinue treatment at any time if the Investigator considers it necessary for any reason including:

- Ineligibility (either arising during the trial or retrospectively having been overlooked at screening)
- An adverse event which requires discontinuation of the trial medication
- Disease progression which requires discontinuation of the trial medication
- Patient or physician decision

The reason for any discontinuation of treatment will be recorded in the case record form.

8.7. Withdrawal from the Study

Patients, or parents/guardians/representatives when they have given consent on behalf of the patient, are free to withdraw consent and stop study participation at any time for any reason. In this case, the patient's clinical management will continue to be provided according to standard care and will not be affected by the decision to withdraw. Outcome data and the reason for withdrawal will be recorded when possible. Data collected up to the date of withdrawal will be used in study analysis.

9. DATA COLLECTION

9.1. Clinical data acquisition

Study dosing, sampling and data collection will begin after enrolment. Figure 3 shows the clinical data acquisition schedule.

Patient demographic data and medical history will be collected for all patients at the time of enrolment in the trial. While a participant is an inpatient, information will be collected daily on signs and symptoms, medications and blood products received, and the results of non-trial blood samples. For patients who are receiving TKM that day, detailed records of physiological monitoring and adverse event monitoring (outlined in section 8.2) will be provided.

SD1 (Study Day 1) is defined as the first day these data are collected. For patients who are in the TKM cohort this is likely to be the same day the first dose is administered.

These data will be collected daily for participating patients up to D14, or discharge, or death, whichever occurs first. Patients who achieve discharge criteria before D14 will stop the inpatient testing schedule upon discharge.

For patients not enrolled in the study, only anonymous survival data will be collected.

Discharge criteria are:

- 72 hours without fever or significant symptoms AND
- A significant improvement in clinical condition AND
- Able to feed, wash and walk independently AND
- Ebolavirus RNA PCR test negative

Figure 3: Clinical data acquisition procedures

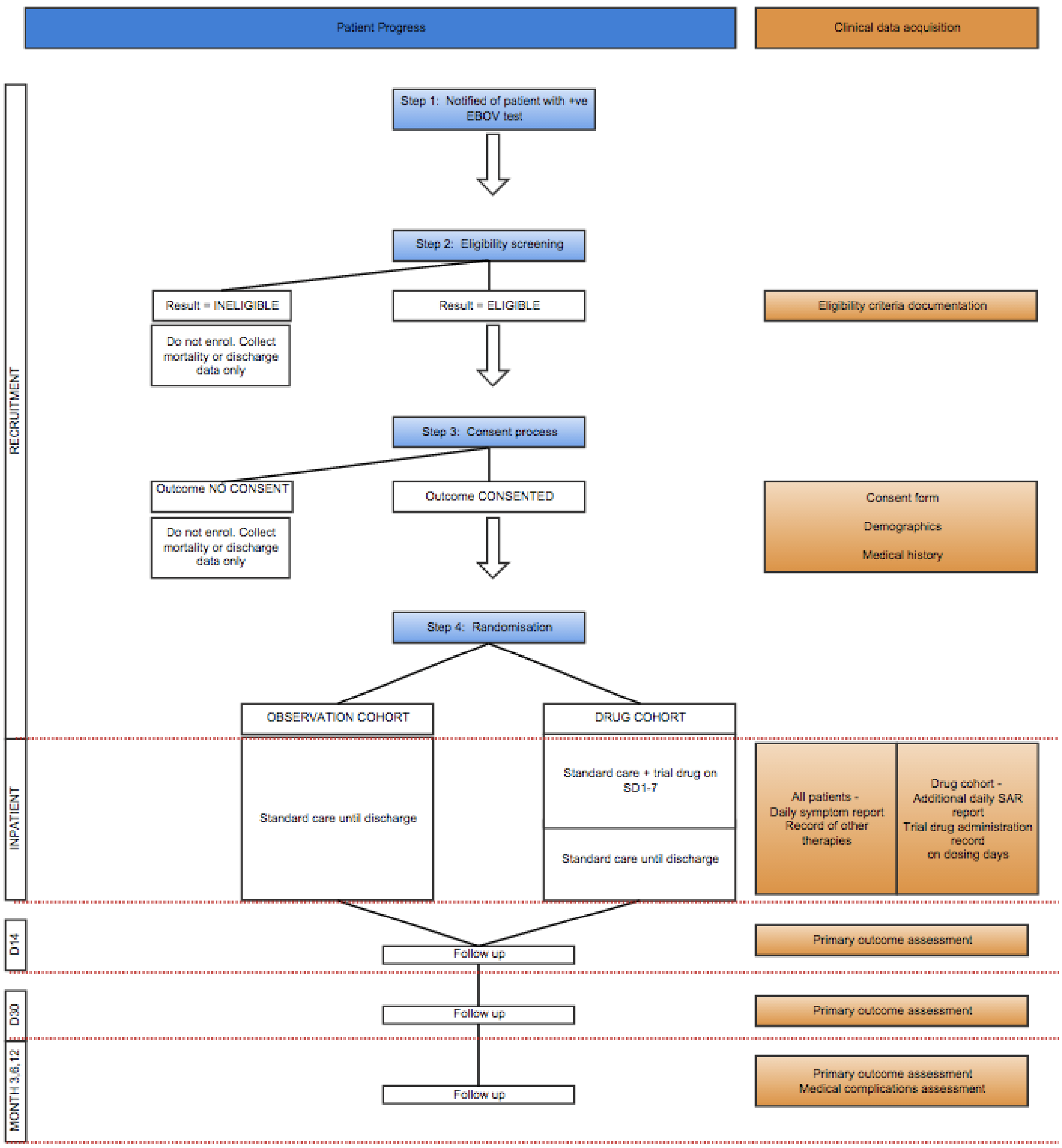

9.2. Follow up Assessments (D14, D30, Months 3, 6, 12)

Survival at D14 is the primary end point of this trial, and defined as survival until 23.59 on Day 14. Follow up assessments will be conducted on D14 and D30 and at 3, 6 and 12 months. Patients who have been discharged before these dates will be issued a mobile telephone for the purposes of follow-up. Patients will be invited to return to the treatment centre for the D14 and D30 visits and then subsequent follow up will occur by phone or at a treatment centre. When patients are not able or willing to attend a follow-up visit on D14 and D30 at the treatment centre, field workers will follow up by phone. Remaining follow up visits will be arranged at a survivor's clinic or suitable local health facility. Patient status on follow-up assessment days will be collected (admitted, met discharge criteria, deceased). In addition, visits at 3, 6, and 12 months will seek information on symptoms post recovery.

Primary/secondary outcome data for these visits can be collected at any point after the respective days.

9.3. Laboratory Assessments

The timing of laboratory sampling for research purposes is outlined in Figure 4.

Figure 4: Scheduling sample for trial specimens – TKM study.

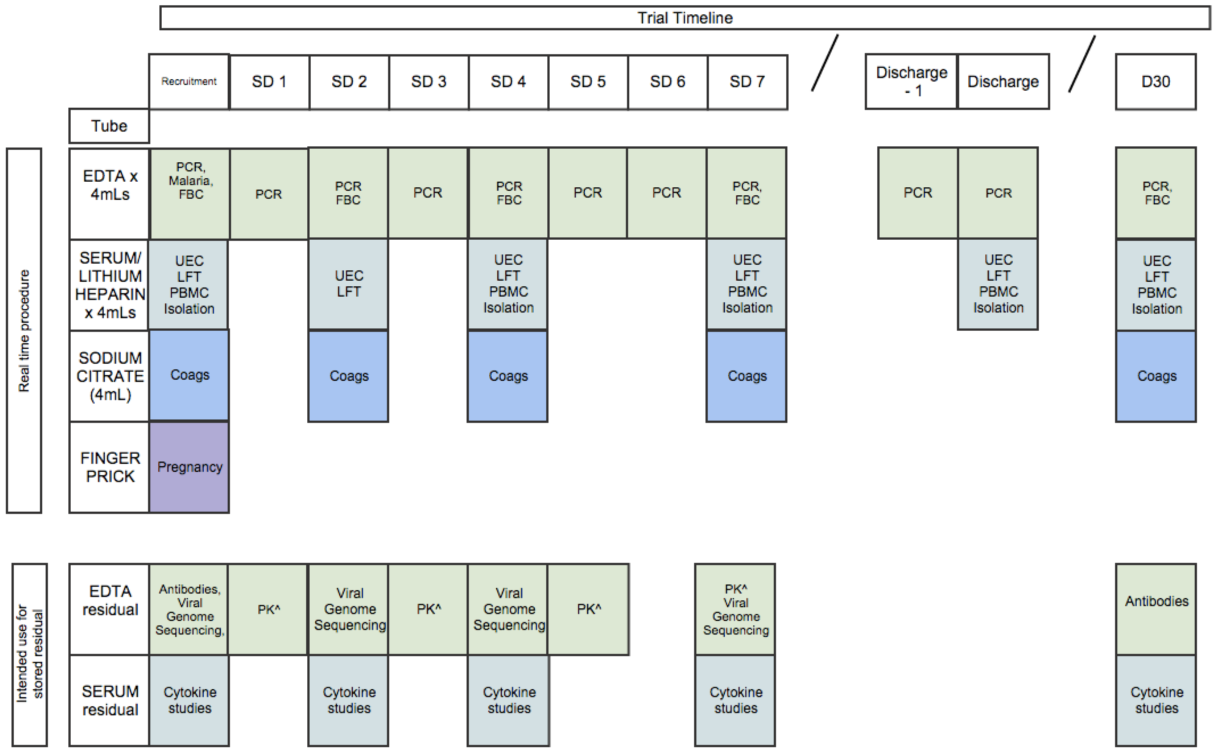

* Timing assumes that patients commence infusion on day 1, following the recruitment day. If commencement of infusion occurs at a later time, the schedule will be altered to reflect this.
^ PK testing if laboratory capacity exists. Patients who have PK testing undertaken will have 2 x EDTA samples drawn.

The only essential blood tests are the diagnostic EBOV positive PCR on admission and EBOV negative PCR prior to discharge (a single, or two negative PCR results will be acceptable, depending on local ETU policy). Adult and pediatric samples will be reduced in volume according to standard procedures such that no patient will have more than 0.6 mls/kg (>1% blood volume) taken on any one day, nor more than 2.4 mls/kg (approximately 3% of blood volume) taken in any four week period. Standard care samples will be prioritised over research samples if volume reduction is required. Ability to take samples is dependent on staff availability, the availability of suitable laboratory facilities and caseload. Research samples and standard care samples may be reduced where required, to maintain care standards and staff safety, and reflect the assays that can be performed by the laboratory attached to the ETC. Other chemical or biological assays may be performed using the same blood volumes if available and useful to inform patient safety or study outcomes.

The same sampling schedule applies to all patients irrespective of their allocation in either the observational or TKM cohort, with the exception of pharmacokinetic drug testing. This will only be performed on patients receiving the trial drug.

Malaria testing: Malaria diagnostic tests will be performed on the triage blood sample as part of standard care. The results of these tests will be recorded.

Pregnancy testing: For women of childbearing age (15-49 years) a chromatographic βHCG test will be performed on the triage blood sample. Urine testing is also acceptable.

Ebola testing: Patients will have blood samples collected at triage for EVD diagnosis by PCR and prior to discharge to confirm clearance of viral RNA, both as standard care. In addition, a study only EDTA will be taken daily for the first 7 days of study involvement (SDs 1-7) for PCR. The PCR result for all samples will be shared with the clinical team in real time. Samples obtained for PCR are typically ≤4 ml whole blood in an EDTA tube; finger or heal-pricks of blood on a dry swab are sometimes obtained when venepuncture is not possible. Ebolavirus RNA will be detected by PCR, as per local clinical laboratory protocols. Additionally, plasma anti-Ebolavirus antibody titers will be determined by analysis of residual volumes by partner laboratories. This is to determine whether the treatment influences the subsequent production of specific antibodies.

Electrolyte, renal and hepatic function testing. Additional testing on SDs 1, 2, 4 and 7 will investigate electrolytes, and renal and hepatic function. Electrolyte disturbances are a common complication of Ebola. Results from toxicological studies indicate TKM may cause hepatic impairment, although this has not been shown in human trials. This information will be provided to clinical staff in real time.

Pharmacokinetic testing. Pharmacokinetic (PK) testing will only be undertaken on patients receiving the study drug. In order to assess the PK of TKM following repeat dosing, it is recommended that blood samples be obtained for PK analysis, if feasible, depending on the capabilities of the ETC, the availability of appropriate laboratory facilities and the clinical condition of the treated individual. It is recommended that PK samples be obtained pre-dose and again at the end of the infusion, on SDs 1, 3, 5, and 7 of treatment. The PK samples should be obtained, processed and stored according to the instructions provided separately, entitled, "Instructions for the Collection, Processing, and Storage of Blood Samples for Pharmacokinetic (PK) Testing" – PK SOP and PK Manual.

Residual volumes. Residual volumes of all samples will be stored and subsequently shipped to international partner laboratories for confirmatory testing and quantification. If volume remains, additional testing relevant to the pathogenesis of EVD or effects of the study treatment will be performed. Residual cells or buffy coat and plasma supernatant buffy coat may be retained for host genetic studies to identify susceptibility and severity markers. Details of sample storage, shipment and custody are in Section 10.2.

10. TRIAL DRUGS

10.1. Formulation

The investigational product, TKM-130803 Injection is presented as an aqueous dispersion of nucleic acid/lipid particles (also referred to as Lipid Nanoparticles, LNP) in a 10 mL United States pharmacopeia (USP) Type I borosilicate glass vial with a Fluorotec®-faced butyl rubber stopper and an aluminum flip-off cap. TKM-130803 is a formulation of siEbola-3 drug substance with lipid excipients. The nucleic acid/lipid particles (LNPs) have an average size of approximately 60 to 90 nm.

The nominal mg/mL drug concentration of the specific batch of TKM-130803 will be recorded on each vial and also on the corresponding storage cartons. **TKM-130803 vials should remain in the cartons protected from light and stored refrigerated at 2 to 8°C** (35.6 to 46.4°F). **DO NOT FREEZE.**

TKM will be administered by a two hour IV infusion. The patient will receive 150mL of TKM-130803 dosing solution in 0.9%NaCl. An infusion pump should be used when administering TKM-130803 to ensure a well-controlled rate of infusion.

For full details of infusion preparation and administration please refer to the relevant trial Standard Operating Procedure manual.

10.2. Storage and Accountability

TKM vials should be stored refrigerated at 2 to 8°C (35.6 to 46.4°F). They must not be frozen.

Inventory, dispensing and accountability of study treatment will be tightly controlled. Treatment dispensed to the Confirmed ward, but not consumed by patients will not be returned due to infection control procedures and will be destroyed. Full accountability procedures and logs will be detailed in the corresponding study reference SOPs.

11. SAFETY REPORTING

Due to the nature of the symptoms of EVD (see **Table 2**) [2] and the relatively small sample size it will be very difficult to differentiate between symptoms of the disease and events due to treatment, and the trial will have limited power to do this. Our investigation of safety will therefore focus on Serious Adverse Reactions (SARs) and Suspected Unexpected Serious Adverse Reaction (SUSARs) and key adverse events [e.g. diarrhoea, vomiting, bleeding, allergic reaction, anaphylaxis, cytokine release, flushing, hypotension and pain (chest pain, back pain, abdominal pain and headache)]. We will collect data using the CRF on these symptoms if they are new or worsening including, as far as possible, a severity grading in line with the CTCAE (Common Terminology Criteria for Adverse Events). These will only be reported to TOG/IDMC in accordance with the safety reporting as described below.

11.1. Definitions for safety reporting

Serious Adverse Reaction (SAR) – A serious untoward and unintended response in a participant to the study treatment, which is related (or has a reasonable possibility of being related) to any dose administered to that participant.

To qualify as “serious” the response must meet one of the following criteria:

- results in death
- is life-threatening
- requires inpatient hospitalisation or prolongation of existing hospitalisation
- results in persistent or significant disability/incapacity
- consists of a congenital anomaly or birth defect.

Other ‘important medical events’ may also be considered serious if they jeopardise the participant or require an intervention to prevent one of the above consequences.

For this protocol, due to the expected high death rate due to EVD, medical events that are not considered related to any dose of study treatment are *not* considered to be Serious Adverse Events (SAEs).

Suspected Unexpected Serious Adverse Reaction (SUSAR) - A serious adverse reaction, the nature and severity of which is not consistent with the information about the medicinal product in question set out in the investigator's brochure (IB).

11.2. Procedures for safety reporting

All SARs will be reported by the site to the operational team within 48 hours. In discussion with the site staff, the operational team will assess the SAR for expectedness and relatedness and report all SUSARs to the independent data monitoring committee (IDMC) immediately. The operational team will also report any SARs and SUSARs to all the relevant parties (such as IRBs, regulatory authorities and Tekmira) as required within 7 days.

Evaluation relatedness of reactions will consider the opinion of the site staff and the expected signs, symptoms and events associated with Ebolavirus disease or trial treatment according to the relevant Investigators' Brochure. Evaluation of expectedness will be made with reference to the Investigator's brochure. Serious infusion reactions will be reported. While the clinical syndrome of cytokine release may mimic other symptoms of EBOV, if suspected and serious, it will be reported to the TOG/IDMC.

Table 2. *Symptoms of Ebolavirus disease (WHO Ebola Response Team, 2014[2]; ISARIC WHO Viral Haemorrhagic Fever CRF)*

Known symptoms and clinical events of EVD:	
Death	Coma or unconsciousness
Fever	Unexplained bleeding
Fatigue	Hematemesis
Loss of appetite	Blood in stool
Vomiting	Bleeding gums
Diarrhoea	Bloody nose
Headache	Bloody cough
Abdominal pain	Other bleeding
Joint pain	Bleeding at injection site
Muscle pain	Blood from vagina
Chest pain	Blood in urine
Cough	Bleeding under the skin/bruising
Difficulty breathing	Back pain
Difficulty swallowing	Decreased urine output
Conjunctivitis	Lower chest wall indrawing
Sore throat	Hearing impairment
Confusion	Tinnitus
Hiccups	Seizures
Jaundice	Hepatomegaly
Eye pain	Splenomegaly
Rash	Lymphadenopathy

12. STATISTICS

12.1. Description of Statistical Methods

The trial is designed to determine whether TKM is a promising treatment for Ebola. No early stopping in the case of evidence of efficacy is proposed, as there are currently only 100 courses of TKM available so

early general roll out of the treatment is not possible. Enrolling the full 100 patients will maximize the precision of the final estimate of efficacy.

A futility design (Whitehead and Matsushita, 2003) is therefore used to allow early stopping in the event of evidence of futility or harm. This approach will recruit up to 100 patients, but will stop if the number of successes observed so far falls below a certain threshold.

In order to avoid early stopping due to enrollment of patients with very severe, late Ebola virus disease, who may not be expected to be salvaged even by an effective antiviral therapy, the stopping rule will be calculated after exclusion of enrolled patients who die within 48 hours of admission to the ETU.

For the purposes of determining futility, the effectiveness of the treatment will be judged in terms of the probability that a patient allocated to TKM treatment will survive to D14, after excluding patients who die within 48 hours of admission to the ETU. This probability will be denoted by p. Note that p represents the true value of the probability rather than any estimate that might be found from the trial data. The value $p = 0.55$ will be used for guidance. If $p \leq 0.55$, then the treatment will be regarded as 'not promising' and the trial will be terminated.

The choice of guide value for p has been made following an analysis of individual level data on patients from a Ebola Treatment Centres from this current outbreak at Gueckedou, Foya, Kailahun and Elwa 3. Data on the outcomes of 1592 patients with laboratory confirmed Ebolavirus infection from these four centres are shown in Table 3. Only data from adults have been included, and data on patients who died on the day of admission or on the following day, or else the day after that have been omitted.

Table 3: *Estimates and 95% confidence intervals for the probability of surviving to Day 14 following admission to the treatment centre, excluding patients who die on the date of admission or on the day after or the day after that. Success is taken to be a difference between the date of death and the date of admission that is > 13 days.*

	**Gueckedou**	**Foya**	**Kailahun**	**Elwa 3**	**Overall**
Lower limit	0.485	0.428	0.514	0.510	0.517
Estimate	0.535	0.495	0.568	0.554	0.543
Upper limit	0.585	0.562	0.622	0.598	0.569

As a result of this analysis, $p = 0.55$ has been set as the success rate threshold above which TKM would be considered promising.

While children are initially excluded from the TKM arm of the trial, if the eligibility criteria is expanded to age >5 following IDMC review of preliminary results, TKM trial data from adults and children will be jointly analysed, since the availability of TKM is restricted to 100 treatment courses and a stratified analysis is infeasible. Case fatality is highest in children aged < 5 years, after which it declines with age reaching a minimum in persons aged about 15-20 years. Therefore the inclusion of subjects aged 5-17 years is not expected to inflate the case fatality, and the futility threshold of $p \leq 0.55$ is expected to remain valid.

12.2. The Number of Participants

The maximum number of participants available to evaluate the drug efficacy is 100. The data will be analysed sequentially and stopping rules applied.

12.3. Criteria for the Termination of the Trial

Research sites will inform the data management centre every time that a patient is allocated to TKM treatment. Fourteen days after admission, they will report to the data management centre whether that patient did, or did not, survive to D14. Every time that a D14 report is received on a patient, a point will be plotted on Figure 5**Error! Reference source not found.**. This is plot of the number of survivals (S) reported o far against the number of D14 reports received (n), after excluding from both S and n those patients allocated to TKM treatment who died within 48 hours of admission. The plot is compared with the lower red boundary shown. If the plot reaches this boundary, the trial will be stopped with the conclusion that treatment with TKM is not promising. This safety plot will ensure a rapid reaction if TKM proves to be ineffective or harmful in the short term.

If the plot continues above the boundary until the green boundary is reached, corresponding to D14 reports being received from 100 patients, then it will be concluded that TKM is a promising treatment. If the trial has to be terminated before 100 patients have received TKM and before the lower red boundary is reached, and if stopping is due to a shortage of eligible patients rather than to any safety concerns, the final dataset will be analysed. If the outcomes of patients recruited are good enough it remains possible for TKM to be found promising, even following an incomplete study. Nevertheless, every effort will be made to complete the study of 100 patients if the lower boundary is not crossed and no important safety concerns emerge.

***Figure 5**. The stopping rule for the trial*

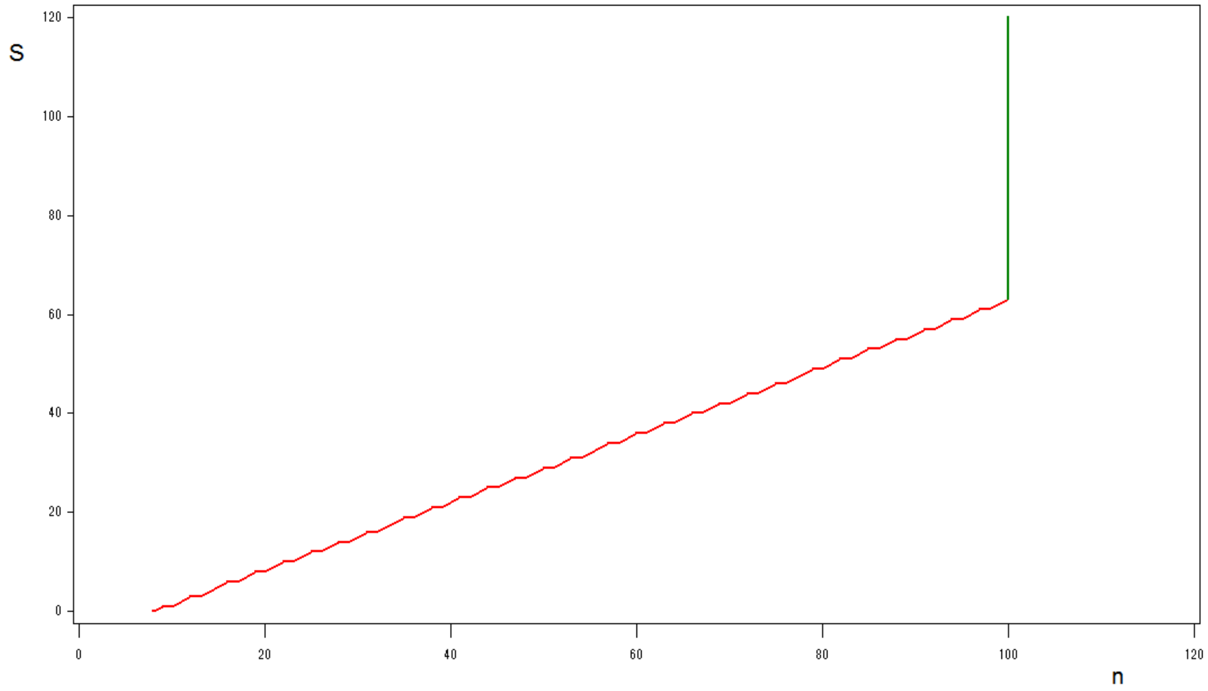

The properties of the design have been calculated exactly, based on the independent Bernoulli distributions of each patient outcome. These are shown in Table 4 and depict the specified probability of reaching the most appropriate conclusion for a range of values of p. It can be seen that the probability is declaring TKM to be promising is set at 0.025 according to this design. Recommending a treatment associated with such a low probability of survival to D14 would be considered a type I error, and the risk

of this has been set at the conventional value of 0.025. The power of correctly identifying TKM as promising when p = 0.70 is 0.827.

Table 4: Probabilities of claiming that TKM is promising for the phase II trial of Treatment TKM

True probability of success (p)	Probability of claiming that TKM is promising
0.50	0.002
0.55	0.025
0.60	0.158
0.65	0.487
0.70	0.827
0.75	0.973
0.80	0.998
0.85	1.000
0.90	1.000

12.4. Final Analysis

When the trial has been completed, a point estimate and a 95% confidence interval will be computed for p using the method of Jovic and Whitehead,[19] which allows for the sequential nature of the trial. If the trial has to be abandoned before the stopping rule is met due to lack of patients, or for any other reason that is independent of the emerging results of the trial, then an underrunning analysis will be used to compute a point estimate and a 95% confidence interval for p. This will also allow for the futility stopping rule imposed (Whitehead, 1992).

12.5. Inclusion in Analysis

It is important to note that the primary sequential analysis described uses only data from patients who were admitted more than 14 days previously. Although it may be known that some more recently recruited patients have already died, their data will not yet be used, to avoid bias due to information on death being available sooner than information on survival. Enrolled patients who die within 48 hours of admission to the ETU will be excluded from the analysis.

12.6. Procedure for Accounting for Missing, Unused, and Spurious Data.

While considerable effort will be made to ensure all patients are followed up at D14, D30, month 3, 6 and 12 (through return visits to clinic, phone, email or field-researcher visits), some patients may not be contactable. Leeway will have to be allowed for late reporting, but this should be limited: it may be that data on survival to D14 is not entered until Day 16 in order to avoid remaining bias due to late reporting of survival. However, if it turns out that a patient died on Day 15 or later, *then they are survivors* for the purpose of this study. If they walked away on Day 7, seemingly cured, it might be assumed that they survived to Day 14. Some judgment will necessarily be used here.

12.7. Rationale for statistical design choice

In the context of the current EVD outbreak, a simple, robust and rapid trial is essential. Mortality dominates all other patient endpoints. Most deaths occur within 14 days of diagnosis, and so survival (or

not) to D14 represents the most relevant clinical endpoint. The situation at Ebola Treatment Centres is highly challenging, and so this study places minimal additional responsibilities on them.

A design with a lower stopping boundary is proposed so that, if the trial is to conclude that TKM is not promising, this can be done as quickly as possible. If all treated patients die before D14, then only 8 patients will be required. No early stopping in the case of evidence of efficacy is proposed, as there are currently only 100 courses of TKM available so that no early general roll out of the treatment is possible. Rapid reporting of minimal information would appear to be feasible, and in keeping with the urgency of the situation. Until the trial is terminated, data managers need only compare the number of survivals observed so far with the stopping boundary**Error! Reference source not found.**. A table of values will be rovided for this purpose.

The approach described is a form of the futility design, as described by Whitehead and Matsushita [23] The analysis is based on an orderings approach introduced by Fairbanks and Madsen.[24] Exact calculations have been made for this particular design, using SAS, and this package will be used for the analysis.

13. DATA MANAGEMENT

13.1. Data Capture

Patient files will serve as source clinical and drug dosing data for the study. Due to infection control procedures, paper documents cannot be taken outside of the high-risk containment zone; therefore records may be dictated to a secondary document outside of the zone and the primary document incinerated. Where possible, electronic data entry will capture source data directly or source documents will be photographed/scanned and archived. Original data files from the site and research laboratories will provide source data for laboratory testing results. Data recorded by clinical staff as a part of the standard case investigation form will be used to support the classification of disease severity as well as for validation and comparison of study data.

Data will be entered to a central study database on MACRO software. Data management, reporting and storage within this trial will comply with the requirements of European Union data protection laws, ICH Good Clinical Practice and FDA 21 CFR Part 11.

13.2. Access to Data

Data sharing will be under the aegis of the Trial Steering Committee and will adhere to the principles of rapid, open access as outlined in the World Health Organisation report on Ethical considerations for use of unregistered Interventions for Ebolavirus disease.[25]

14. QUALITY ASSURANCE PROCEDURES

Governance of the trial will be through the Trial Steering Committee (TSC), the Trial Operation Group (TOG) and the Independent Data Monitoring Committee (IDMC).

- The TSC will be the primary decision making body, with membership from each partner institution, a senior representative from each participating country, and other independent experts. Members of the TSC are given in Section 1 (members may change if necessary). Decisions of the Committee

will be by consensus. WHO will be part of the Committee on an ex-officio basis. Terms of reference for the TSC will set out the full details of the membership, operations and remit.

- The TOG will be led from the ISARIC Coordination Centre in Oxford and report to the TSC. This team will provide operational oversight and day-to-day management of the trial to ensure compliance to follow up and critical factors such a retention and data quality. This team will comprise those with responsibility for the operational activities and so include representation from each site, data management, trial operations, training and logistical support, laboratory sample processing and handling.
- The IDMC will be responsible for reviewing the data from the study. Membership will include individuals with statistical, clinical and trial expertise. An IDMC charter will fully set out the committee's remit, membership and full definition of their operational conduct in terms of reviewing efficacy and safety data and reporting their recommendations to the TSC.

The trial will be conducted in accordance with the current approved protocol, relevant regulations and standard operating procedures. Regular monitoring will be performed to ensure quality control. Data will be evaluated for compliance with the protocol and accuracy. Following written standard operating procedures, the monitors will verify that the clinical trial is conducted and data are generated, documented and reported in compliance with the protocol and the applicable regulatory requirements.

The MACRO software will be configured to automatically cross-validate across entire patient records according to the specific requirements. Data Clarification requests will be built in to the system. The data validation and quality processes will comply with the new FDA and EU recommendations for data level trial monitoring and therefore allow for data quality to be assured in real time as a mechanism for onsite monitoring.

Investigators and clinical staff treating centre are required to provide direct access to source data/documents for trial-related monitoring, audits, ethics committee review and regulatory inspection. All trial related and source documents must be kept for at least five years after the end of the trial.

15. ETHICAL AND REGULATORY CONSIDERATIONS

15.1. Regulations, Guidelines and Approval

The Investigators will ensure that this study is conducted in compliance with the current revision of the Declaration of Helsinki and the applicable principles of International Committee on Harmonization Good Clinical Practice Guidelines. Regulatory approval to conduct the study and import investigational products will be obtained from national authorities. The protocol, informed consent form and investigators brochure will be submitted to the Sierra Leone Ethics and Scientific Review Committee , the Oxford Tropical Research Ethics Committee, and where necessary, the Ebola Treatment Centre funding organisations ethics board, for written approval.

The Chief Investigator will submit and, where necessary, obtain approval from the above parties for all substantial amendments to the original approved documents.

15.2. Considerations in Informed Consent

STANDARD CARE UPON PRESENTATION: Patients are triaged to Suspected and Probable wards upon presentation based on symptom and contact history. Blood is drawn for Ebolavirus testing according to standard care at the site. Results are reported back to the clinical staff, who then visit patients to discuss the results. When laboratory results positive for Ebolavirus are received from the laboratory, a senior member of medical staff and a psycho-social worker visit the patient to discuss the results. Clinical and social counseling are provided as the patient is moved to the Confirmed ward. Recruitment to the study will not interfere with the standard care process. Research staff will be available to support ETC workers for study related tasks and to ensure that standard care is not disrupted by study procedures.

PROCESS FOR TAKING INFORMED CONSENT: Written informed consent to participate in the observational and randomise cohorts will be required from all participants or their representative. The staff who undertake the discussion of test results will decide when the patient should be approached regarding the study. This discussion should occur as soon as possible after the availability of confirmed results, but at a time when the patient is emotionally stable and the staff feel that they are capable of considering the risks and benefits of participation. Informed consent can be delayed for up to 48 hours following arrival to the ETC.

All eligible patients (section 7) should be offered the opportunity to participate in the study. When the clinical staff and/or social workers agree that an adult patient is mentally and physically fit to discuss and consider participation in the study, the patient or the parent/guardian of a patient under 18 years, will be approached. If an eligible patient has reduced consciousness or is unresponsive to discussion due to illness severity, an appropriate representative will be approached for consent if available. Appropriate representatives will be selected according to the standard Ebola Treatment Centre processes for determining who can make decisions on a patient's behalf. In the case of a child who has no parent/guardian or in the case of a patient who is unfit to consent and there are no appropriate representatives available proxy consent will be sought from an independent adult, or group of adults (two or three) who have agreed to act together to give proxy consent. This person/ group member could be a doctor who is not a member of the research team, or other suitable adult willing to act in this role, who has already agreed to undertake this role and has been fully briefed on the trial.

Consent will be requested by the local study staff or another suitable qualified and trained person authorised to do so by the Principal Investigator.

ADULTS: Adult patients for the purpose of consent are defined as ≥18 years of age. Adults who are deemed fit to discuss and consider participation by the medical and social staff will give consent independently. If an adult is not considered mentally and physically fit to give informed consent, consent will be sought from a close relative who attends the clinic or an appointed representative. When consent is obtained from a relative, the patient will be approached for consent if at any time during study participation their condition improves such that they can consider consent.

CHILDREN: Children are patients less than 18 years of age. Consent for children to participate in the study will be sought from the parents or guardians of the child or an appointed representative in the case of a child without a contactable parent/guardian. Study participation will be discussed at the discretion of the clinical and social staff with children of suitable physical and mental fitness as well as emotional maturity. When consent is obtained from a representative, a parent/guardian will be approached for consent if at any time during study participation the study staff are able to contact them.

DISCUSSING CONSENT: Participants or their representative will be presented with an informed consent form that explains the purpose, methods, risks and benefits of the study. The form will be available in

relevant written local languages. If the patient or representative cannot read, the form will be read to them in the presence of a witness when available. If a consent form is not available in a language familiar to the participant, staff will verbally translate the form into the language of the patient. Standardization of translation will be supported by the use of recorded verbal translations with appropriate language and explanations in local terms that will be used to train study staff. When possible, a second member of staff will be present during the informed consent discussion to ensure that translations are appropriate, the form is read accurately, that all of the patient's questions have been addressed and to act as witness when possible. This will be facilitated by the paired working system at the treatment centres, but may not always be possible due to human resource limitations and limited shift times.

DOCUMENTING CONSENT: Patients or their representative will be asked to sign and date an informed consent form prior to participation. Those unable to write will be asked to make a mark on the signature line only. If a witness was present during the consent process s/he will co-sign the consent form to confirm the accurate reading of the form and the consent of the participant/parent/guardian/representative. If consent for an adult patient is given by a representative, patients will be asked to sign a separate informed consent form when/if they are deemed physically and mentally fit at a later point during the study. When a second member of staff is present for the informed consent discussion, s/he will sign the informed consent form. A child's assent to participate will not be recorded.

ARCHIVING OF INFORMED CONSENT FORMS: Due to infection control procedures, paper cannot be transferred externally from the confirmed wards. Therefore informed consent forms will be photographed and archived electronically, then destroyed according to waste disposal procedures for contaminated substances. Patients will be given either a printed copy of the original signed form if possible or an unsigned copy to retain upon discharge.

15.3. Participant Confidentiality

The trial staff will ensure that the participants' anonymity is maintained. All study documents and samples will be labeled with an anonymous study code. Identifying information collected as a part of this study will remain confidential. For the purpose of ensuring data integrity and to facilitate quality assurance, study records will link to clinic files, which include patient's name and other identifying information. Participants' names will be recorded at the time of enrolment to allow for their identification at follow-up visits. However identifiable information will be linked to stored data or samples only by a protected Master List. This list will not be shared outside of the site study staff and no identifying information will be transferred between sites. All CRFs and samples will be labeled with a study identification number only and stored in suitable secure locations. Only persons who have signed the locally appropriate data protection training will have access to the password-protected computer where entered data is stored. After conclusion of the project, data will be removed from the computers and stored in a safe place. Any scientific publications or reports will not identify any patient by name or initials. When the research team reviews their notes, they are also bound by professional confidentiality. All study data will be stored in secure databases only accessible to study staff. Study sponsors and health authorities will be given controlled access for the purpose of audit when necessary.

15.4. Sample Confidentiality and Management

Aliquots of blood will be stored initially at the site clinical diagnostic laboratory. Subsequently, aliquots will be shipped to national and/or international partner laboratories according to national and international

regulations. Samples will be anonymised with a unique study identifier prior to shipping and any patient identifiable data will have been removed. The Country Principal Investigator and Chief Investigator will have access to the enrolment log linking study identifiers with patient identifiers. Material transfer agreements and export/import licenses (where required) will be obtained, and international regulations on storage and shipping of hazardous samples will be followed. Sample custody will be maintained by the investigators and decisions regarding use and transfer of samples will be made by the TSC. Research samples will be stored indefinitely and approval from the sponsor and/or ethics committees, as appropriate, will be sought prior to destruction. The study investigators are responsible for biological deactivation and ensuring appropriate destruction of any residual materials.

15.5. Risks and Benefits

There are no approved treatments for EVD and the standard of care remains supportive care and treatment of complications. An analysis of data on 3343 confirmed and 667 probable EVD cases estimates a case fatality rate of 70.8% (95% confidence interval [CI], 69 to 73).[2] Unpublished data from MSF indicates that the survival rate in laboratory confirmed cased of Ebolavirus infection admitted to Gueckedou Ebola Treatment Centre is not likely to be more than 50% overall (see statistics section).

The potential efficacy, compared with safety and tolerability of the trial drug, is outlined in section 5.2. Potential AEs that require increased clinical surveillance are outlined in 8.2.

Of note, the potential teratogenicity of TKM is unknown. To date, the fatality in foetus' of pregnant women with EVD is not known to be less than 100%. Pregnant women will not be included in the TKM trial initially. This will be reviewed by the TSC following enrolment and treatment of the first 15 patients.

TKM has never been used in children. In the initial stage of the study eligibility to the TKM arm will be restricted to patients 18 years of age or older until the first 15 patients are enrolled and treated. At that time all available, relevant safety data will be reviewed by the IDMC and a recommendation may be made to extend the eligibility to patients 5 years or older or to some other intermediate age between 5 to 18 years, as recommended by the IDMC.

Potential provision to younger patients, children under the age of 5, will be discussed with an expert panel.

The risks of conducting this trial include the possibility that the operation of the Ebola Treatment Centre (ETC) where the trial is conducted is compromised by any of the following events: a.) the ETC is overwhelmed by suspected EVD cases referred or self-referring in the belief that an effective treatment is available at the ETC; b.) the ETC security is breached by persons seeking access to the investigation product; c.) the security and safety of the ETC and ETC staff are compromised if there is a perception that the investigational product or the clinical trial are harmful to individual or community interests.

Whilst these are significant risks they are not in themselves arguments for not conducting this trial, since these risks will exist for all ETCs operating in this setting, the risks may be most acute for the earliest trials conducted.

Mitigation of these risks will be achieved by the following activities: A) Community sensitization and participation in the run up to trial initiation. Experienced health promotion and community outreach workers will conduct this activity. B) Study drug will be stored in a locked metal cabinet within a secure building. C) Contingency plans for handling an increase in the number of suspected cases will be developed, including strict criteria for closing to new admissions at the site conducting the study and the

identification of satellite ETC centers to accept patients that cannot be acceptable at the ETC conducting the trial. D) Evacuation plans will be in place in the event of a major security breach.

15.6. Expenses

Clinic and treatment costs are covered by the ETC funding organisation (and partner laboratories) for all patients presenting to the Ebola Treatment Centre regardless of study participation. The study drug will be supplied without cost to the patient. Patients will be issued a mobile phone for the purposes of follow-up. This phone and SIM card will become the property of the patient. Reasonable travel expenses and compensation for loss of earnings will be paid for attending follow-up at the Ebola Treatment Centre. No other funding or incentives for participation will be given to the patients.

15.7. Contemporaneous Studies

Patients cannot enroll to any other clinical trial that involves a therapeutic or care intervention while they are participating in this study. If participating sites are undertaking observational studies, patients may be co-enrolled provided the combined study procedures are considered safe and appropriate for the patients and there is no conflict of outcomes or endpoints between the studies.

15.8. Alternatives to Study Participation

All patients will be treated with the best available care regardless of study participation. Patients are free to decline participation in this study without effect on the standard care provided.

15.9. Community Engagement

Standard interventions likely to be used by the managing organisation include dissemination of health promotion messages to health authorities and communities regarding ETC activities and information on EVD transmission, prevention, reporting and response. Information on this research study would be added to these messages.

Messages to the community in EVD regions are disseminated by networks of Health Promotion workers who engage with local leaders that serve as primary sources of information for the communities. Radio broadcasting and printed information sheets and posters are also common tools. This standard protocol on dissemination of critical messages would be used to inform the communities about research done at the Ebola Treatment Centre. Information regarding the nature and purpose of the research would be distributed with a variety of tools across these networks.

Education on the current development status of EVD treatments, clinical research studies ongoing in West Africa and the scientific background and methods of the current project would be distributed to and discussed with the health authorities already engaged as a part of the Health Promotion activities.

Most treatment centres also provide psychological and social support to EVD patients. When presenting to the Ebola Treatment Centre, patients and families will be approached by a team social worker to facilitate the psychological process throughout admission, EVD confirmation, treatment and when required, death and bereavement. Patients who are discharged from the Treatment Centre will be supported in their return to the community. This team will be engaged in the research study according to

the requirements of the sites to assist with explaining the nature and methods of the research, obtain informed consent, and discuss the study with the family.

16. FINANCE AND INSURANCE

16.1. Funding

The trial is funded by the Wellcome Trust.

16.2. Insurance

The University of Oxford has arranged appropriate insurances to provide for the University's responsibilities, as Sponsor, to research subjects; and, to cover the legal liabilities of the University to those engaged by the University in the performance of this research. The University will also arrange, or arrange in conjunction with other participating partners those emergency medical repatriation facilities which can be achieved, subject to the exigencies of arranging such at the material time.

17. PUBLICATION POLICY

Results from the trial will be published in open access and the data will be available for sharing.

18. REFERENCES

1. WHO. Ebola Response Roadmap Situation Report 1. 29 August 2014. http://www.who.int/csr/disease/ebola/situation-reports/29-august-2014.pdf?ua=1. Accessed 3rd September 2014.
2. WHO Ebola Response Team. Ebola virus disease in West Africa--the first 9 months of the epidemic and forward projections. *N Engl J Med.* 2014;371(16):1481-1495.
3. Kucharski AJ, Edmunds WJ. Case fatality rate for Ebola virus disease in west Africa. *Lancet.* 2014;384(9950):1260.
4. Gire SK, Goba A, Andersen KG, et al. Genomic surveillance elucidates Ebola virus origin and transmission during the 2014 outbreak. *Science.* 2014;345(6202):1369-1372.
5. Fowler RA, Fletcher T, Fischer WA, 2nd, et al. Caring for critically ill patients with ebola virus disease. Perspectives from west Africa. *Am J Respir Crit Care Med.* 2014;190(7):733-737.
6. Mahanty S, Bray M. Pathogenesis of filoviral haemorrhagic fevers. *Lancet Infect Dis.* 2004;4(8):487-498.
7. Takada A, Kawaoka Y. The pathogenesis of Ebola hemorrhagic fever. *Trends Microbiol.* 2001;9(10):506-511.
8. Bausch DG, Towner JS, Dowell SF, et al. Assessment of the risk of Ebola virus transmission from bodily fluids and fomites. *J Infect Dis.* 2007;196 Suppl 2:S142-147.
9. Towner JS, Rollin PE, Bausch DG, et al. Rapid diagnosis of Ebola hemorrhagic fever by reverse transcription-PCR in an outbreak setting and assessment of patient viral load as a predictor of outcome. *J Virol.* 2004;78(8):4330-4341.
10. Ebihara H, Rockx B, Marzi A, et al. Host response dynamics following lethal infection of rhesus macaques with Zaire ebolavirus. *J Infect Dis.* 2011;204 Suppl 3:S991-999.
11. Rollin PE, Bausch DG, Sanchez A. Blood chemistry measurements and D-Dimer levels associated with fatal and nonfatal outcomes in humans infected with Sudan Ebola virus. *J Infect Dis.* 2007;196 Suppl 2:S364-371.
12. Wauquier N, Becquart P, Padilla C, et al. Human fatal zaire ebola virus infection is associated with an aberrant innate immunity and with massive lymphocyte apoptosis. *PLoS Negl Trop Dis.* 2010;4(10).
13. Oestereich L, Ludtke A, Wurr S, et al. Successful treatment of advanced Ebola virus infection with T-705 (favipiravir) in a small animal model. *Antiviral Res.* 2014;105:17-21.
14. Qiu X, Wong G, Audet J, et al. Reversion of advanced Ebola virus disease in nonhuman primates with ZMapp. *Nature.* 2014.
15. Warren TK, Wells J, Panchal RG, et al. Protection against filovirus diseases by a novel broad-spectrum nucleoside analogue BCX4430. *Nature.* 2014;508(7496):402-405.
16. Sayburn A. WHO gives go ahead for experimental treatments to be used in Ebola outbreak. *BMJ.* 2014;349:g5161.
17. Geisbert TW, Lee AC, Robbins M, et al. Postexposure protection of non-human primates against a lethal Ebola virus challenge with RNA interference: a proof-of-concept study. *Lancet.* 2010;375(9729):1896-1905.
18. Geisbert TW, Hensley LE, Kagan E, et al. Postexposure protection of guinea pigs against a lethal ebola virus challenge is conferred by RNA interference. *J Infect Dis.* 2006;193(12):1650-1657.
19. Jovic G, Whitehead J. An exact method for analysis following a two-stage phase II cancer clinical trial. *Stat Med.* 2010;29(30):3118-3125.
20. Clopper CJ, Pearson ES. The use of confidence or fiducial limits illustrated in the case of the binomial. *Biometrika.* 1934;26:404-413.
21. Bross IJ, Delaney MM. Significance tests for certain 2 x 2 tables. *Am J Hyg.* 1952;55(3):357-362.
22. Whitehead J. *The Design and Analysis of Sequential Clinical Trials*. Revised 2nd edition ed. Chichester: Wiley; 1997.

23. Whitehead J, Todd S. The double triangular test in practice. *Pharmaceutical Statistics.* 2004;3(1):39-49.

24. Fairbanks K, Madsen R. P values for tests using a repeated significance test design. *Biometrika.* 1982;69:69-74.

25. WHO. *Ethical considerations for use of unregistered interventions for Ebola viral disease. Report of an advisory panel to WHO.* [Online]. 2014. Available from: http://apps.who.int/iris/bitstream/10665/130997/1/WHO_HIS_KER_GHE_14.1_eng.pdf?ua=1. [Accessed 9th September 2014].

26 Whitehead, J. Overrunning and underrunning in sequential clinical trials. Controlled Clinical Trials 13, 106-121 (1992).

Trial Title: Rapid Assessment of Potential Interventions & Drugs for Ebola (RAPIDE) – TKM

Scientific Title: Open-label, single arm trial to investigate the efficacy of TKM-130803 with a concurrent observational study of Ebolavirus Disease in Sierra Leone.

Short title: Evaluation of TKM Treatment for Ebolavirus Disease

Date and Version No: 2nd June, 2015 v 2.9

Chief Investigator: **Peter Horby**
Centre for Tropical Medicine and Global Health
Nuffield Department of Medicine Research Building
University of Oxford
Old Road Campus, Roosevelt Drive
OXFORD, OX3 7FZ

Country Principal Investigator: **Foday Sahr** 34 Military Hospital, Ministry of Health and Sanitation Freetown, Sierra Leone

Co-Investigators: **James Russell**, Sierra Leone College of Medicine and Allied Health Sciences

G.F. Deen, Sierra Leone College of Medicine and Allied Health Sciences

Tim Brooks, Rare and Imported Pathogens Laboratory, Public Health England

Trudie Lang Global Health Network, Centre for Tropical Medicine and Global Health, University of Oxford

Piero L Olliaro Newton-Abraham Visiting Professor, University of Oxford.

Jake Dunning (Field Clinical Lead) Centre for Tropical Medicine and Global Health, University of Oxford

John Whitehead Department of Mathematics and Statistics, Lancaster University

Sponsor: University of Oxford

Joint Research Office, Block 60, Churchill Hospital, Oxford, OX4 7LE

Funder: Wellcome Trust

PARCT: PACTR201501000997429

TABLE OF CONTENTS

1. KEY TRIAL CONTACTS 4
2. SYNOPSIS 6
3. ABBREVIATIONS 8
4. INTRODUCTION 9
5. DRUG INFORMATION 10
5.1. Efficacy data against EBOV 10
5.2. Safety and tolerability 10
5.3. Dosing and dosing rationale 11
6. OBJECTIVES AND OUTCOME MEASURES 12
7. TRIAL DESIGN 12
7.1. Trial design 12
7.2. Trial design rationale 13
7.3. Eligibility for TKM 13
7.4. Trial enrolment 13
7.5. Participant inclusion criteria 16
8. TRIAL PROCEDURES 17
8.1. Screening and informed Consent 17
8.2. In-patient Days 17
8.3. Follow up Assessments (D14, D30, Months 3, 6, 12) 18
8.4. Procedures for provision of TKM 18
8.5. Laboratory Samples 19
8.6. Procedures for standard care 19
8.7. Treatment Interruption 19
8.8. Discontinuation of Trial Treatment 19
8.9. Withdrawal from the Study 20
9. DATA COLLECTION 20
9.1. Clinical data acquisition 20
9.2. Laboratory Assessments 21
10. TRIAL DRUGS 23
10.1. Formulation 23
10.2. Storage and Accountability 24
11. SAFETY REPORTING 24

11.1. Definitions for safety reporting 24
11.2. Procedures for safety reporting 25
12. STATISTICS 25
12.1. Description of Statistical Methods 26
12.2. The Number of Participants 26
12.3. Criteria for the Termination of the Trial 27
12.4. Final Analysis 28
12.5. Inclusion in Analysis 28
12.6. Procedure for Accounting for Missing, Unused, and Spurious Data. 28
12.7. Rationale for statistical design choice 28
13. DATA MANAGEMENT 29
13.1. Data Capture 29
13.2. Access to Data 29
14. QUALITY ASSURANCE PROCEDURES 29
15. ETHICAL AND REGULATORY CONSIDERATIONS 30
15.1. Regulations, Guidelines and Approval 30
15.2. Considerations in Informed Consent 30
15.3. Participant Confidentiality 32
15.4. Sample Confidentiality and Management 32
15.5. Risks and Benefits 33
15.6. Expenses 34
15.7. Contemporaneous Studies 34
15.8. Alternatives to Study Participation 34
15.9. Community Engagement 34
16. FINANCE AND INSURANCE 35
16.1. Funding 35
16.2. Insurance 35
17. PUBLICATION POLICY 35
18. REFERENCES 36

1. KEY TRIAL CONTACTS

**Chief Investigator**	Associate Professor Peter Horby<br><br>Centre for Tropical Medicine and Global Health<br>Nuffield Department of Medicine Research Building<br>University of Oxford<br>Old Road Campus, Roosevelt Drive<br>OXFORD<br>OX3 7FZ<br><br>Tel: 07990 560237<br>Email: Peter.horby@ndm.ox.ac.uk
**Sponsor**	University of Oxford<br>Joint Research Office<br>Block 60, Churchill Hospital<br>Oxford, OX4 7LE
**Statistician**	Professor John Whitehead<br><br>Department of Mathematics and Statistics<br>Fylde College<br>Lancaster University<br>Lancaster LA1 4YF<br><br>Tel: 01524 389967<br>Email: j.whitehead@lancaster.ac.uk
**Committees**	**Trial Steering Committee Voting Members:**<br><br>**Samuel Kargbo**<br>Ministry of Health and Sanitation, Freetown, Sierra Leone<br><br>**Mohamed Samai,**<br>College of Medicine and Allied Health Sciences, Freetown, Sierra Leone<br><br>**Foday Sahr**<br>34 Military Hospital, Ministry of Health and Sanitation Freetown, Sierra Leone<br><br>**Stephen Kennedy**<br>Pacific Institute for Research and Evaluation, Monrovia, Liberia<br><br>**Fred Binka** (Independent Member)<br>University of Health and Allied Sciences, Ho, Ghana<br><br>**Peter Horby** (Chief Investigator)

	Centre for Tropical Medicine and Global Health, Oxford, United Kingdom<br>International Severe Acute Respiratory and emerging Infections Consortium<br><br>**John Whitehead** (Trial Statistician)<br>Lancaster University, Lancaster, United Kingdom<br><br>**Rob Fowler** (Independent Member)<br>Sunnybrook Health Sciences Centre, Toronto, Canada<br><br>**Nicholas White** (Independent Member - Chairperson)<br>Mahidol-Oxford Research Unit, Bangkok, Thailand<br><br>**Fiona Gannon**<br>GOAL Programme Quality Advisor, Republic of Ireland<br><br>**Non-Voting Members**<br>**Ana Maria Henao-Restrepo** (WHO Representative)<br>World Health Organization, Geneva, Switzerland<br><br>**Independent Data Monitoring Committee (IDMC) Members:**<br><br>**David Lalloo** (Chair), Professor of Tropical Medicine; Dean of Clinical Sciences and International Public Health, Liverpool School of Tropical Medicine<br><br>**Clement Adebamowo,** Director Office of Strategic Information and Research Institute of Human Virology in Nigeria<br><br>**Donald Berry,** Founder and senior Statistical Scientist, Berry Consulting<br><br>**Nicolas Opoku,** Director Onchocerciasis Chemotherapy Research Centre, Hohoe, Ghana<br><br>**Peter Smith,** Professor of Tropical Epidemiology, London School of Hygiene and Tropical Medicine<br><br>**Durodami Radcliffe Lisk,** Professor Consultant Neurologist, Ministry of Health Coordinator, Epilepsy Association of Sierra Leone, College of Medicine and Allied Health Sciences (COMAHS), Sierra Leone

2. SYNOPSIS

Trial Title	**Rapid Assessment of Potential Interventions & Drugs for Ebola (RAPIDE) – TKM**	
Scientific Title	Open-label, single arm trial to investigate the efficacy of TKM-130803 with a concurrent observational study of Ebolavirus Disease in an outbreak setting in West Africa	
Internal ref. no. (or short title)	Evaluation of TKM-130803 Treatment for Ebolavirus Disease	
Clinical Phase	2	
Trial Design	Open-label, single arm trial, with a concurrent observational study	
Trial Participants	Patients with confirmed Ebolavirus disease attending the participating treatment centre	
Planned Sample Size	Up to 100 adult patients (with possible late inclusion of paediatric patients)	
Treatment duration	1 week	
Follow up duration	12 months follow up	
	Objectives	Outcome Measures
Primary	TKM: To evaluate the impact of TKM treatment on early mortality in EVD<br><br>Observational: To characterize the early mortality of EVD in an ETC	Mortality at Day (D)14 (in those that survive first 48 hours after admission)<br><br>Mortality at D14 (in those that survive first 48 hours after admission)
Secondary	TKM<br>1. To evaluate the impact of TKM-130803 treatment for adults on:<br>a) Overall morality at D14<br>b) Time to recovery<br>c) Late mortality<br>d) Viral load<br>e) EVD symptoms<br>f) EVD antibody response<br>g) Long term clinical recovery<br><br>2. i)To assess the safety of TKM treatment for adults.<br>ii) To measure the pharmacokinetics (PK) of TKM following repeat dosing	TKM<br>1. a) D14 mortality in all patients allocated to TKM-130803 treatment (not excluding deaths in first 48 hours).<br>b) Time to meeting ETC discharge criteria.<br>c) Mortality at D30 and months 3,6,12 after first dose of study treatment<br>d) Viral load<br>e) Presence and duration of symptoms (SDs 1-14)<br>f) Anti-Ebolavirus IgG titre (recruitment and D30)titre<br>g) Clinical assessment at D30 and months 3, 6, 12.<br><br>2. Incidence of SARs, key adverse events (SDs 1-14) and monitoring of vital signs (pulse rate, blood pressure, respiratory rate, temperature) pre, during and at 0, 1, 2, 4 and 8 hours post end of infusion

	Observational<br>To characterise the natural history of EVD in an ETC.<br>a) Time to recovery<br>b) Late mortality<br>c) Viral load<br>d) EVD symptoms<br>e) EVD antibody response<br>f) Long term clinical recovery	ii) PK pre-dose and at the end of infusion on SDs 1, 3, 5 and 7<br><br>Observational<br>a) Time to meeting ETC discharge criteria.<br>b) Mortality at D30 and months 3,6,12 after admission<br>c) Viral load<br>d) Presence and duration of symptoms (SDs 1-14)<br>e) Anti-Ebolavirus IgG titre (recruitment and D30)titref) Clinical assessment at D30 and months 3, 6, 12.
Investigational Medicinal Product	TKM-130803 (TKM)	
Formulation, Dose, Route of Administration	TKM is provided as 0.3 mg/kg, administered as a 2-hour intravenous infusion (IV), in a final volume of 150ml, once daily for seven days.<br><br>To assess the feasibility and safety of dosing with TKM-130803 in an ETU, data on the first four enrolled patients (the safety cohort) will be assessed by the IDMC prior to opening enrollment to additional patients. The safety cohort may be expanded following advice from the IDMC.<br><br>In the event of suspected drug-related toxicity or a change in the patient's clinical condition, the dose of TKM-130803 may be reduced to a minimum of 0.24 mg/kg once daily at the discretion of the treating physician. TKM will not be given to children or pregnant women during the initial phase of the study.<br><br>Administration of the drug will adhere to the relevant trial Standard Operating Procedure (SOP) document.	

3. ABBREVIATIONS

AE	Adverse event
CI	Confidence Interval
Coags	Coagulation studies
CRF	Case Report Form
D	Days (since admission)
EBOV	Ebolavirus
EDTA	Ethylenediaminetetraacetic acid
ETC	Ebola Treatment Centre
EVD	Ebolavirus Disease
FBC	Full blood count
GCP	Good Clinical Practice
IB	Investigators Brochure
ICF	Informed Consent Form
ICH	International Conference on Harmonisation
IDMC	Independent Data Monitoring Committee
IMP	Investigational Medicinal Product
ITT	Intention to Treat
IV	Intravenous
LFT	Liver function tests
MAD	Multiple Ascending Dose
MSF	Medecins Sans Frontieres
OXTREC	Oxford Tropical Research Ethics Committee
PI	Principal Investigator
PK	Pharmacokinetic
REC	Research Ethics Committee
RNA	Ribonucleic acid
RT-PCR	Reverse transcription polymerase chain reaction
SAD	Single Ascending Dose
SAE	Serious Adverse Event
SAR	Serious Adverse Reaction
SD	Study days
SOP	Standard Operating Procedure
SUSAR	Suspected Unexpected Serious Adverse Reactions
TKM	TKM-130803
TSC	Trial Steering Committee
UEC	Urea, Electrolytes, Creatinine
WHO	World Health Organisation

4. INTRODUCTION

The size and scale of the on-going Ebola Virus Disease (EVD) outbreak is unprecedented, and has been declared a Public Health Emergency of International Concern.[1] EVD is among the most virulent infectious agents known: an analysis of data on 3343 confirmed and 667 probable EVD cases collected during the current outbreak in Guinea, Liberia, Nigeria, and Sierra Leone estimates a case fatality rate of 70.8% (95% confidence interval [CI], 69 to 73) among persons with known clinical outcome of infection.[2] A figure that is consistent with estimates of other authors.[3] Figure 1 shows the key time points in the clinical course of EVD in this outbreak.[2]

Figure 1. Estimated mean (median) time course in days of Ebolavirus infection in this outbreak[2]

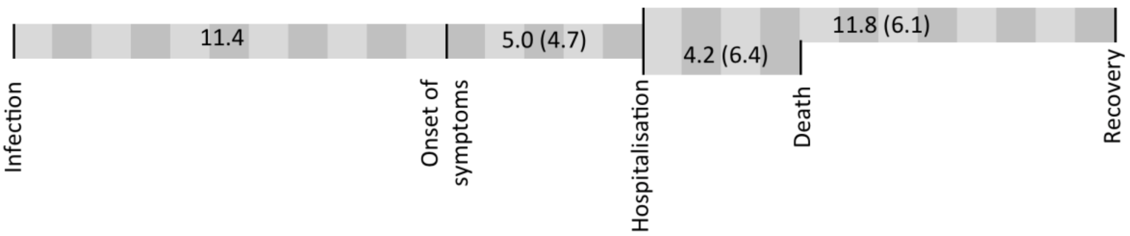

The current Ebolavirus strain causing the outbreak in West Africa is related to the Ebola Zaire strain and genetic analysis suggests the current outbreaks in Guinea, Sierra Leone and Liberia are all related to a single transmission event from a zoonotic source followed by human-to-human transmission.[4] EVD is characterized by a febrile illness dominated by fatigue and gastrointestinal symptoms that can be complicated by shock, haemorrhage and multi-organ failure. However, clinical presentation and severity is variable, with some patients remaining relatively well, able to ambulate and self-feed throughout their illness, whilst others progress rapidly to a fatal outcome.[2,5]

The pathogenesis of EVD is incompletely understood[6,7] but high levels of viral replication and the detection of virus in multiple body tissues is typical of severe disease.[8,9] Coagulopathy, disruption of endothelial function and increased inflammatory responses are also associated with severe EVD.[6,10-12] The association between high levels of viraemia and EVD severity suggests that therapies that target viral replication may benefit patients. Several experimental products that target Ebolavirus replication have shown some efficacy in animal studies.[13-15] However, whilst several therapeutic interventions have shown promise in the laboratory and in animal studies, none have been tested for efficacy and safety in humans with EVD. A World Health Organization (WHO) expert panel recently concluded unanimously that “investigators have a moral duty to evaluate these interventions in the best possible clinical studies that can be conducted under the circumstances of the epidemic.”[16]

The aim of this protocol is to assess the effectiveness of the drug TKM-130803 (produced as TKM-130803 by Tekmira Pharmaceuticals) for the treatment of EVD.

The Trial Steering Committee selected TKM as one of the leading drugs suitable for urgent evaluation from a possible list of 10 candidate therapies. The criteria for selection were based upon a Target Product Profile that considered existing clinical and pre-clinical data supporting safety and efficacy, immediate availability and scalability, and an acceptable treatment regimen.

The purpose of this trial is to establish whether TKM is a promising treatment for Ebola Virus Disease.

5. DRUG INFORMATION

TKM-130803 is a drug that has been developed specifically to target EBOV. TKM-130803 is a lipid nanoparticle formulation of 2 small interfering RNAs (siRNAs) directed against Ebolavirus L polymerase and Viral Protein-35 (VP-35). These two proteins are involved in transcription and replication of EBOV. TKM-130803 is a new formulation of drug TKM-100802 (formerly TKM-100201), which has been adapted to improve its specificity to the Guinea variant viral strain causing the current outbreak by a two nucleotide swap in the VP35 siRNA and a single nucleotide swap in the L-pol siRNA to ensure complete match to the emergent strain. TKM-100201 and TKM 100802 have been evaluated in guinea pig, non-human primate, and human Phase 1 clinical trials. TKM-130803 treatment of Rhesus monkeys at 72 hours following challenge with a lethal dose of Ebola virus Guinea 2014 led to 67% survival (2/3 animals). TKM-100802 drug has received authorization for emergency use in EVD patients from the US Food and Drug Administration. 100 treatment courses of TKM-130803 are available for this trial, in a wet (non-lyophilized) formulation.

5.1. Efficacy data against EBOV

In-vitro activity of TKM-100802 was established using two variants of EBOV infecting a human hepatoma cell line. The EC50 ranged between <0.003nM and 0.04nM. The therapeutic index was determined to be between 1513 and >18954 for treatment commencing one hour following viral inoculation. Molecular analysis confirmed the proposed mechanism of activity of the two siRNAs. In-vivo trials have established that TKM-100802 has an inhibitory impact on virus replication in EBOV-infected cells, as well as a survival benefit in EBOV-infected guinea pigs and rhesus macaques[17,18]. In rhesus monkeys infected with a lethal challenge of Zaire EBOV, 100% survival has been observed in animals that received TKM-100802 at 0.5 mg/kg/day for 7 days, with 67% survival at 0.2 mg/kg/day for 7 days. In rhesus monkeys infected with a lethal challenge of Guinea variant EBOV at 0.5 mg/kg/day for 7 days, TKM-130803 (siEbola-3 with LNP1) resulted in 33% survival (1/3) with treatment commencing at 48 hours post inoculation, and 67% (2/3) with treatment commencing at 72 hours post inoculation.

5.2. Safety and tolerability

TKM-130803 has the same formulation as TKM-100802 except for two nucleotide swaps in the VP35 siRNA and a single nucleotide swap in the L-pol siRNA. Therefore the safety and tolerability profile is expected to be similar to TKM-100802. For a detailed description of the safety and tolerability of TKM-100802, please refer to the Investigator Brochure and additional safety summary. Adverse events (AEs) considered most likely to occur with TKM-130803 include discomfort, bruising, and bleeding at the site of infusion, and adverse effects analogous to those observed in animal studies, including coagulopathies, renal impairment, hepatobiliary injury and acute phase reaction.

In a human single ascending dose trial of TKM-100201 administered over one hour to healthy participants, infusion-related reactions, notably cytokine release syndromes (CRS) were observed. Clinical manifestations included flushing, headache, fever, hypotension, chills, nausea, and vomiting. Most of the reported treatment related AEs were consistent with a transient inflammatory response that begins within 6 hours after infusion and dissipates in most cases by 24 hours post-dosing. This interpretation is consistent with laboratory findings of transient elevations in some cytokines (e.g., MCP-1, IL-6, IL-1ra, IL-8) in some patients measured 2 and 6 hours post-dosing, returning to at or near baseline levels by 24 hours. In

addition, focal and reversible pain has been observed in association with liposomal or LNP therapeutics, particularly in the back, sacrum and sternum.

Fertility and reproductive toxicity studies have not been performed with TKM-100802 or TKM-130803.

5.3. Dosing and dosing rationale

In rhesus monkeys infected with a lethal challenge of EBOV, 100% survival has been observed in animals that received TKM-100802 at 0.5 mg/kg/day for 7 days, with 67% survival at 0.2 mg/kg/day for 7 days. In rhesus monkeys infected with a lethal challenge of Guinea variant EBOV at 0.5 mg/kg/day for 7 days, TKM-130803 (siEbola-3 with LNP1) resulted in 33% survival (1/3) with treatment commencing at 48 hours post inoculation, and 67% (2/3) with treatment commencing at 72 hours post inoculation. Comparisons of drug exposure data from monkeys and healthy human volunteers indicate that plasma exposure parameters (C_{max} and AUC) are approximately equivalent between humans and monkeys when dosed at the same dose level of TKM-100802 (based on weight; mg/kg). Thus, it is anticipated that dose levels of at least 0.2 mg/kg/day will be required to meet exposure targets derived from efficacious dose levels currently used in monkey EBOV infection studies.

The no-observed-adverse-effect-level (NOAEL) of TKM-100802 in a repeat-dose toxicity study in cynomolgus monkeys was 0.25 mg/kg/day after daily 1-hour intravenous infusion of TKM-100802 for 14 days. The total cumulative dose (3.5 mg/kg) at the NOAEL is equivalent to the maximum cumulative dose proposed in humans (i.e., 0.5 mg/kg/day × 7 days; 3.5 mg/kg). The highest dose level (1.0 mg/kg/day for 14 days) was well tolerated in monkeys, but resulted in microscopic findings in the liver, spleen, and kidneys, which were not severe, but precluded consideration of this dose level as a NOAEL.

The safety and pharmacokinetics of TKM-100802 have been investigated in a single ascending dose (SAD) phase of Study TKM-EBOV-002 (A Placebo-Controlled, Single-Blind, Single Ascending Dose Study With Additional Multiple Ascending Dose Cohorts To Evaluate The Safety, Tolerability, and Pharmacokinetics of TKM-100802 In Healthy Human Volunteers) over a dose range from 0.075 mg/kg to 0.5 mg/kg. The maximum tolerated dose (MTD) was determined to be 0.3 mg/kg, using a conservative toxicity grading scale for healthy volunteers.

TKM-130803 will be administered by IV infusion at a rate of 1.25 mL/min, administered over approximately 120 minutes for a total volume of 150mls administered. For infusion related adverse reactions, the infusion rate may be slowed or interrupted at the discretion of the treating physician. If the infusion is interrupted and resumed, the total infusion should be completed within 4 hours of infusate preparation. The patient will be in recumbent position for dose administration. TKM will be administered via a dedicated peripheral IV or central line.

For full details of infusion preparation and administration please refer to the relevant trial Standard Operating Procedure manual.

6. OBJECTIVES AND OUTCOME MEASURES

	Objectives	Outcome Measures
Primary	TKM: To evaluate the impact of TKM treatment on early mortality in EVD<br><br>Observational: To characterize the early mortality of EVD in an ETC	Mortality at D14 (in those that survive first 48 hours after admission)<br><br>Mortality at D14 (in those that survive first 48 hours after admission)
Secondary	TKM<br>1. To evaluate the impact of TKM-130803 treatment for adults on:<br>a) Overall morality at D14<br>b) Time to recovery<br>c) Late mortality<br>d) Viral load<br>e) EVD symptoms<br>f) EVD antibody response<br>g) Long term clinical recovery<br><br>2. i)To assess the safety of TKM treatment for adults.<br>ii) To measure the pharmacokinetics (PK) of TKM following repeat dosing<br><br>Observational<br>To characterise the natural history of EVD in an ETC.<br>a) Time to recovery<br>b) Late mortality<br>c) Viral load<br>d) EVD symptoms<br>e) EVD antibody response<br>f) Long term clinical recovery	TKM<br>1. a) D14 mortality in all patients allocated to TKM-130803 treatment (not excluding deaths in first 48 hours).<br>b) Time to meeting ETC discharge criteria.<br>c) Mortality at D30 and months 3,6,12 after first dose of study treatment<br>d) Viral load<br>e) Presence and duration of symptoms (SDs 1-14)<br>f) Anti-Ebolavirus IgG titre (recruitment and D30)titre<br>g) Clinical assessment at D30 and months 3, 6, 12.<br><br>2. Incidence of SARs, key adverse events (SDs 1-14) and monitoring of vital signs (pulse rate, blood pressure, respiratory rate, temperature) pre, during and at 0, 1, 2, 4 and 8 hours post end of infusion<br>ii) PK pre-dose and at the end of infusion on SDs 1, 3, 5 and 7<br><br>Observational<br>a) Time to meeting ETC discharge criteria.<br>b) Mortality at D30 and months 3,6,12 after admission<br>c) Viral load<br>d) Presence and duration of symptoms (SDs 1-14)<br>e) Anti-Ebolavirus IgG titre (recruitment and D30)titre<br>f) Clinical assessment at D30 and months 3, 6, 12.

Note that there are two counting systems for days in the trial: days from admission (prefixed with D) and study days (prefixed with SD) from time of first data collection (see section 9.1).

7. TRIAL DESIGN

7.1. Trial design

This research consists of a single-arm, open-label trial of TKM and a concurrent observational trial.

Ongoing observation of the clinical manifestations and outcomes of patients in ETCs is valuable, especially when data acquisition is undertaken in a systematic manner as part of trial.

7.2. Trial design rationale

Due to production capacity limits there are currently only 100 treatment courses of TKM-130803 available for this trial.

The risk of infusion reactions means that TKM-130803 must be infused over a minimum 2-hour period during which clinical monitoring for infusion reactions is necessary. The intensity of clinical monitoring required means that infusions will, in most cases, only be undertaken in the morning (when the day shift of clinical staff and the trial staff are all on site). Even then, the challenges of clinical care delivery within an ETC means that the number of participants who can take part in the study, either receiving TKM-130803 infusions or in the observational cohort, and be safely monitored on any one day may be limited by staffing availability. ETCs may also vary in their capacity to recruit study participants based on fluctuating patient-staff ratios, and inpatient numbers. Therefore, the maximum number of patients receiving TKM-130803 on any one day, and the total number of observational patients, will need to be capped. The lead physician at the ETC will notify the maximum number of monitored beds available for the trial on any given day. Once the bed availability is known, if the number of patients eligible for TKM-130803 exceeds capacity they will be allocated to receive either the drug with standard care (as part of an interventional group) or standard care (as part of an observational cohort) or standard care. The methodology for allocating cohorts is described in section 7.3.

7.3. Eligibility for TKM

Since TKM-130803 has never been used in children and the bio-distribution and pharmacokinetics is not known in this population, in the initial stage of the study, eligibility to the TKM arm will be restricted to patients 18 years of age or older until the first 15 patients have been enrolled and treated. At that time, all available and relevant safety data will be reviewed by the IDMC and a recommendation may be made to extend the eligibility to patients 5 years or older or to some other intermediate age between 5 to 18 years.

Similarly, there have been no fertility or reproductive toxicity studies on TKM – 130803. Hence pregnant and breastfeeding women will also be initially excluded from the trial. After the first 15 patients have been enrolled and treated, the data will be reviewed by the TSC and the TSC may recommend to include pregnant women or continue to exclude them from this trial. If the TSC recommend inclusion of pregnant women, Ethical Review Board approval will be sought.

TKM is administered as an infusion. If reliable IV access cannot be obtained for a patient, they will not be eligible to receive the trial drug nor take part in the observational cohort.

7.4. Trial enrolment

Trial enrolment and allocation to treatment or observational cohort will take place as described in Figure 2 and below.

1. All ETC patients with a laboratory confirmed case of EBOV who can tolerate trial procedures and in whom IV access is possible will be eligible for randomisation into the observational trial.

Patients who are for end of life care only, or who are a late transfer from another facility (and already recovering from EVD) will not be eligible.

2. Patients will also be assessed for eligibility for drug allocation according to the criteria in section 7.3.
3. Allocation into this drug eligible cohort will occur by the following process.
    i. Following arrival of the trial staff each morning, the maximal number of study beds (b) available for patients to receive study drug will be determined by the ETC clinical lead and the trial clinical lead.
    ii. Whenever possible, patient dosing will be started within 2 hours of the trial team arrival in the morning, in order to provide adequate time for patient monitoring and to complete the required assessments during the working day. Consent and any randomisation will need to be completed before this time. If a patient chooses to delay consent, or consent cannot be obtained e.g. family or proxy not available, consent and randomisation of other patients will proceed. Any patients who cannot consent will be considered for enrolment the following day if this is still within the required 48 hour window, see Section 8.1.
    iii. Following consent of patients who have arrived with or received PCR confirmation in the preceding 48hrs, each patient eligible for TKM-130803 treatment will be randomly allocated a number (n) from 1 to N, where N is the number of patients eligible (using a random number allocation program code in R statistical software).
    iv. Patients will be allocated to the treatment cohort by sequentially selecting patients by their number (n) from 1 to b. The remainder of patients will be put forward for the observational cohort (when N>b).
    v. If on the calendar day of randomisation a patient allocated to TKM-130803 treatment is unable to receive treatment (e.g. because they refuse, die, or IV access cannot be obtained), the next patient in the sequence may be entered in to the treatment arm. This may only occur on the calendar day of randomisation.
    vi. Re-randomisation to receive study drug is not permitted.
4. Allocation into the observational cohort will occur by the following process.
    i. Following arrival of the trial staff each morning, the maximal number of observational study beds (b) available for patients will be determined by the ETC clinical lead and the trial clinical lead.
    ii. Patients who are eligible to receive study drug and have been randomised to the observational cohort will be again randomly allocated a number (n) from 1 to N, where N is the number of patients eligible (using a random number allocation program code in R statistical software).
    iii. Patients will be allocated to the observational cohort by sequentially selecting patients by their number (n) from 1 to b. The remainder of patients will be put forward for standard care (when N>b).
        iv. If after randomisation there is one or more observational beds unallocated, randomisation of other patients identified as eligible for the observational cohort will be performed (e.g. patients that are not eligible for randomization into TKM, but only ever directly eligible for the observational cohort such as pregnant women). These patients will also be allocate a number (n) from 1 to N , where N is the number of patients eligible (again using a random number allocation program code in R statistical software).

v. Patients will again be allocated to the observational cohort by sequentially selecting patients by their number (n) from 1 to b, until the unallocated observational beds have been filled.

vi. If on the calendar day of randomisation a patient allocated to the observational arm is unable to take part (e.g. because they refuse, die, or IV access cannot be obtained), the next patient in the sequence may be entered in to the observational arm. This may only occur on the calendar day of randomisation.

5. Patients who are eligible for either the treatment or observational cohorts but are not randomised to either arm will not be included in the study and will receive standard care.

Figure 2. Process of assessing eligibility and patient allocation to treatment or observational cohort

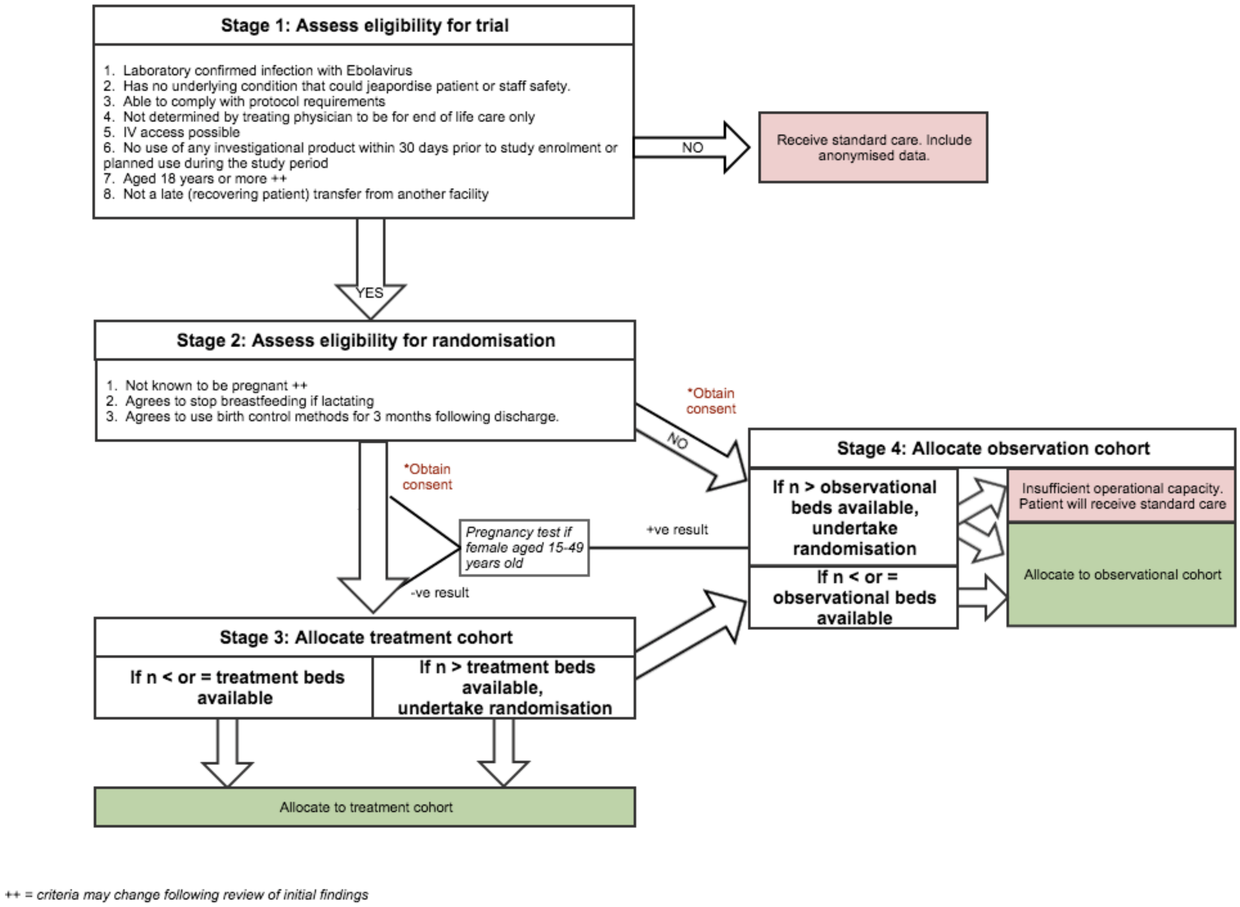

To assess the feasibility and safety of dosing with TKM-130803 in an ETU, data on the first four enrolled patients will be assessed by the IDMC prior to opening enrollment to additional patients. These patients (termed the safety cohort) may be recruited from one or multiple study sites. Each patient will receive at least 3 doses before dosing of the next patient starts. If a patient dies prior to receiving 3 doses, recruitment of the next patient may occur earlier. The IDMC will review data according to the schedule in Table 1 below. If the 4th patient dies prior to receiving 3 doses, the IDMC review may be brought forward. The safety cohort may be expanded following advice from the IDMC.

Table 1: Safety Cohort recruitment schematic.

													IDMC			
Patient 1	X	X	X	X	X	X	X									
Patient 2				X	X	X	X	X	X	X						
Patient 3							X	X	X	X	X	X	X			
Patient 4										X	X	X	X	X	X	X
Patient 5														X	X	X
Patient 6														X	X	X

**Patients 5 and 6 are recruited once recruitment is open following review by the IDMC.*

7.5. Participant inclusion criteria

The eligibility is a two-step process, first for the trial and then for the allocation to the drug eligible and observational cohorts. Patient ages <18 years will be excluded from both TKM treatment and observational cohorts until subsequent time as the IDMC recommends otherwise.

Eligibility criteria for the trial (stage 1):

Inclusion criteria

- Laboratory confirmed infection with Ebolavirus
- Adult aged ≥ 18 years (although the age range may be extended to >5years old following IDMC review of initial findings).
- Has no underlying condition that could jeopardize patient or staff safety
- Able to comply with protocol requirements
- Not determined by treating physician to be for end of life care only.
- IV access possible
- No use of any investigational or non-registered product within 30 days prior to study enrolment or planned use during the study period.

Exclusion criteria

- Determined, through a collaborative decision between the TCL and ETC medical lead, to be clinically recovering .

From within this cohort, additional inclusion criteria for randomization to the TKM cohort (stage 2):

- Not pregnant (although this might change after TSC review of the first 15 patients)
- Not breastfeeding (for lactating women)
- Agrees to use birth-control for 3 months post study.

The study of TKM treatment will concern the outcomes of all patients who are allocated to TKM treatment. For these patients, D1 will be the day of admission to the treatment centre. The primary response for the trial will be survival to D14 (yes or no). Patients who are allocated to TKM treatment and survive the first 48 hours after admission but do not actually receive it or who fail to complete the course of therapy, for any reason, will nevertheless be included in this primary analysis.

The observational study will include those considered eligible for TKM treatment but who are not allocated to it due to the limited number of available treatment beds or those patients otherwise eligible for the

study but do not comply with stage 2 of the eligibility criteria, up to the maximum number of available observational beds. For the analysis of the outcomes of these patients, D1 will be the day of admission to the treatment centre.

Data from participants who for any reason are ineligible for the trial or refuse to participate will be included (anonymously) in the primary analysis of the observational group. All patient outcome data is routinely recorded in all ETCs and shared anonymously with WHO, therefore the use of anonymised D14 outcome data is in line with current practices.

8. TRIAL PROCEDURES

8.1. Screening and informed Consent

Patients with RT-PCR confirmed Ebolavirus RNA (confirmed patients) will be screened for trial eligibility and invited to give informed consent to participate, according to the procedures outlined in section 15.2. Staff will not discuss informed consent with patients (or the parents/representatives of patients <18 years old) who are not eligible for enrolment in any trial arm. The number of patients who are ineligible will be recorded anonymously. Study dosing, sampling and data collection will begin only after enrolment.

A patient may be consented to take part in the study anytime within 48 hours of first arriving at the ETC with a confirmed diagnosis, or, within 48 hours of being informed of Ebolavirus positive PCR result, if this occurs while already at the centre. Therefore, if a patient has been excluded for a reason that resolves within this period, they may be approached upon resolution of the reason for exclusion. If a patient wishes to delay their decision regarding consent, they may consent at any time within the 48 hour period.

Whenever possible, patient dosing will be started within 2 hours of the trial team arrival in the morning, in order to provide adequate time for patient monitoring and to complete the required post dose assessments. The actual time of dosing will depend on various factors, including number of patients on the study and staffing levels in the ETC. Consent and any randomisation will need to be completed before this time. If a patient chooses to delay consent, or consent cannot be obtained e.g. family or proxy not available, consent and randomisation of other patients will proceed. Any patients who cannot consent will be considered for enrolment the following day if this is still within the required 48 hour window.

A list of eligible patients will be produced each day (following receipt of the laboratory results).

Once the randomisation process is complete, to either the TKM treatment or observational cohorts, re-randomisation is not permitted.

8.2. In-patient Days

Standard safety assessments of patients will include daily monitoring of vital signs (pulse rate, blood pressure, respiratory rate, temperature), symptoms and SARs whilst they remain in the ETC. Any non-study medication received will also be recorded.

In addition, for the TKM treatment cohort, vital signs and assessment of possible infusion related reactions will be assessed at the following approximate time points: pre-infusion, during the infusion (preferably between 30-90 minutes), at the end of infusion (0 hour), and at 1, 2, 4 and 8 hours post end of infusion, as well as at additional time points if indicated by the patient's clinical condition.

The recruitment process for the safety cohort is described in Section 7.4. The IDMC will review data from the safety cohort prior to recruitment being opened to all eligible patients. The safety cohort may be expanded following advice from the IDMC.

8.3. Follow up Assessments (D14, D30, Months 3, 6, 12)

Survival at D14 is the primary end point of this trial, and defined as survival until 23.59 on Day 14. Follow up assessments will be conducted on D14 and D30 and at 3, 6 and 12 months. Patients who have been discharged before these dates will be issued a mobile telephone for the purposes of follow-up. Patients will be invited to return to the treatment centre for the D14 and D30 visits and then subsequent follow up will occur by phone or at a treatment centre. When patients are not able or willing to attend a follow-up visit on D14 and D30 at the treatment centre, field workers will follow up by phone. Remaining follow up visits will be arranged at a survivor's clinic or suitable local health facility. An outreach team may attempt to visit participants who are unable to attend a clinic or cannot be reached by phone, if it is considered safe and feasible to do so. Reasonable expenses for travel and loss of earnings will be paid at these follow up visits.

Patient status on follow-up assessment days will be collected (admitted, met discharge criteria, deceased). In addition, visits at D30 and months 3, 6, and 12 will seek information on symptoms post recovery. Trial staff will notify appropriate healthcare providers if the patient reports new symptoms that require further clinical assessment.

Primary/secondary outcome data for these visits can be collected at any point after the respective days.

8.4. Procedures for provision of TKM

Patients randomized into the treatment cohort will receive TKM-130803 in addition to the supportive therapy provided to all patients.

TKM-130803 will be administered at a dose of 0.3mg/kg by IV infusion at a rate of **1.25 mL/min** administered over approximately 120 minutes in a total volume of 150ml. Infusions should be administered using an infusion pump, to ensure a well-controlled rate of infusion.

For full details of infusion preparation and administration please refer to the trial Standard Operating Procedure for drug administration.

Doses will be administered once daily, for seven days. In the event of suspected drug-related toxicity or a change in the patient's clinical condition, the dose of TKM-130803 may be reduced to a minimum of **0.24 mg/kg** once daily at the discretion of the treating physician. If the infusion is interrupted and resumed, the total infusion should be completed within 4 hours of infusate preparation. The patient will be in recumbent position for dose administration.

There are two potential adverse outcomes that require close clinical supervision.

1. Infusion related reactions may occur following commencement of the infusion. As the drug is given once a day, the timing of administration will be set to coincide with the presence of the trial staff in the treatment area. This will allow monitoring of symptoms and a decision to slow or halt the infusion if

necessary. Pre-medication such as paracetamol may be given. IM adrenalin will also be made available for the event of an anaphylactic reaction.

2. Cytokine release syndrome may occur several hours after the infusion commences. Health care workers involved in care of patients receiving TKM will receive additional training to recognise the constellation of symptoms that are associated with it (flushing, headache, fever, hypotension, chills, nausea and vomiting). Management of a cytokine related event will prioritise immediate care (i.e. stopping of infusion (if ongoing), provision of IV fluids) and prompt medical review of the infusion speed and dose. Additional drugs (including steroids) may be given to ameliorate symptoms, at the treating physician's discretion. Procedures for safety reporting are outlined in 11.2.

8.5. Laboratory Samples

All patients, both in the observational and TKM cohort, will have regular blood samples drawn and analysis performed; see Section 9.2 Laboratory Assessments. Also, any residual volumes will be stored. Those in the observational cohort will have all the laboratory tests as for those in the TKM cohort; malaria, pregnancy (for women of childbearing age), ebola testing, electrolyte, renal and hepatic function testing. However, they will not have PK samples taken; these will only be taken from patients receiving the trial drug.

8.6. Procedures for standard care

The supportive therapy received by patients will not be affected by participation, or non-participation, in the trial. All patients will receive standard supportive therapy and for those patients in the study, this will be recorded. Treatment provided by clinical staff is based on the ETC guidelines for management of EVD. This includes oral and/or intravenous fluid, resuscitation, empirical antibiotics, antimalarial agents, and also symptom control, which includes antiemetics, antipyretics/analgesics, and anti-diarrhoeal agents, as appropriate to the patient's symptoms.

8.7. Treatment Interruption

In the event of a SUSAR or other significant treatment related event, the treating physicians will determine if study treatment will continue on the basis of clinical picture and severity and resolution of the event. Consultation with the Independent Data Monitoring Committee will be made in the case of uncertainty to determine if the treatment should be discontinued.

If there is an indication of a cytokine response in the safety cohort (first 4 patients) then recruitment will be reviewed. The IDMC will review data from the complete cohort prior to recruitment being opened to all suitable patients. The safety cohort may be expanded following advice from the IDMC.

8.8. Discontinuation of Trial Treatment

Each participant has the right to discontinue use of study treatment at any time. In addition, the Investigator may discontinue treatment at any time if the Investigator considers it necessary for any reason including:

- Ineligibility (either arising during the trial or retrospectively having been overlooked at screening)
- An adverse event which requires discontinuation of the trial medication

- Disease progression which requires discontinuation of the trial medication
- Patient or physician decision

The reason for any discontinuation of treatment will be recorded in the case record form.

8.9. Withdrawal from the Study

Patients, or parents/guardians/representatives when they have given consent on behalf of the patient, are free to withdraw consent and stop study participation at any time for any reason. In this case, the patient's clinical management will continue to be provided according to standard care and will not be affected by the decision to withdraw. Outcome data and the reason for withdrawal will be recorded when possible. Data collected up to the date of withdrawal will be used in study analysis.

9. DATA COLLECTION

9.1. Clinical data acquisition

Study dosing, sampling and data collection will begin after enrolment. Figure 3 shows the clinical data acquisition schedule.

Patient demographic data and medical history will be collected for all patients at the time of enrolment in the trial. While a participant is an inpatient, information will be collected daily on signs and symptoms, medications and blood products received, and the results of non-trial blood samples. For patients who are receiving TKM that day, detailed records of physiological monitoring and adverse event monitoring (outlined in section 8.2) will be provided.

SD1 (Study Day 1) is defined as the first day these data are collected. For patients who are in the TKM cohort this is likely to be the same day the first dose is administered.

These data will be collected daily for participating patients up to D14, or discharge, or death, whichever occurs first. Patients who achieve discharge criteria before D14 will stop the inpatient testing schedule upon discharge.

For patients not enrolled in the study, only anonymous survival data will be collected.

Discharge criteria are:

- 72 hours without fever or significant symptoms AND
- A significant improvement in clinical condition AND
- Able to feed, wash and walk independently AND
- Ebolavirus RNA PCR test negative

Figure 3: Clinical data acquisition procedures

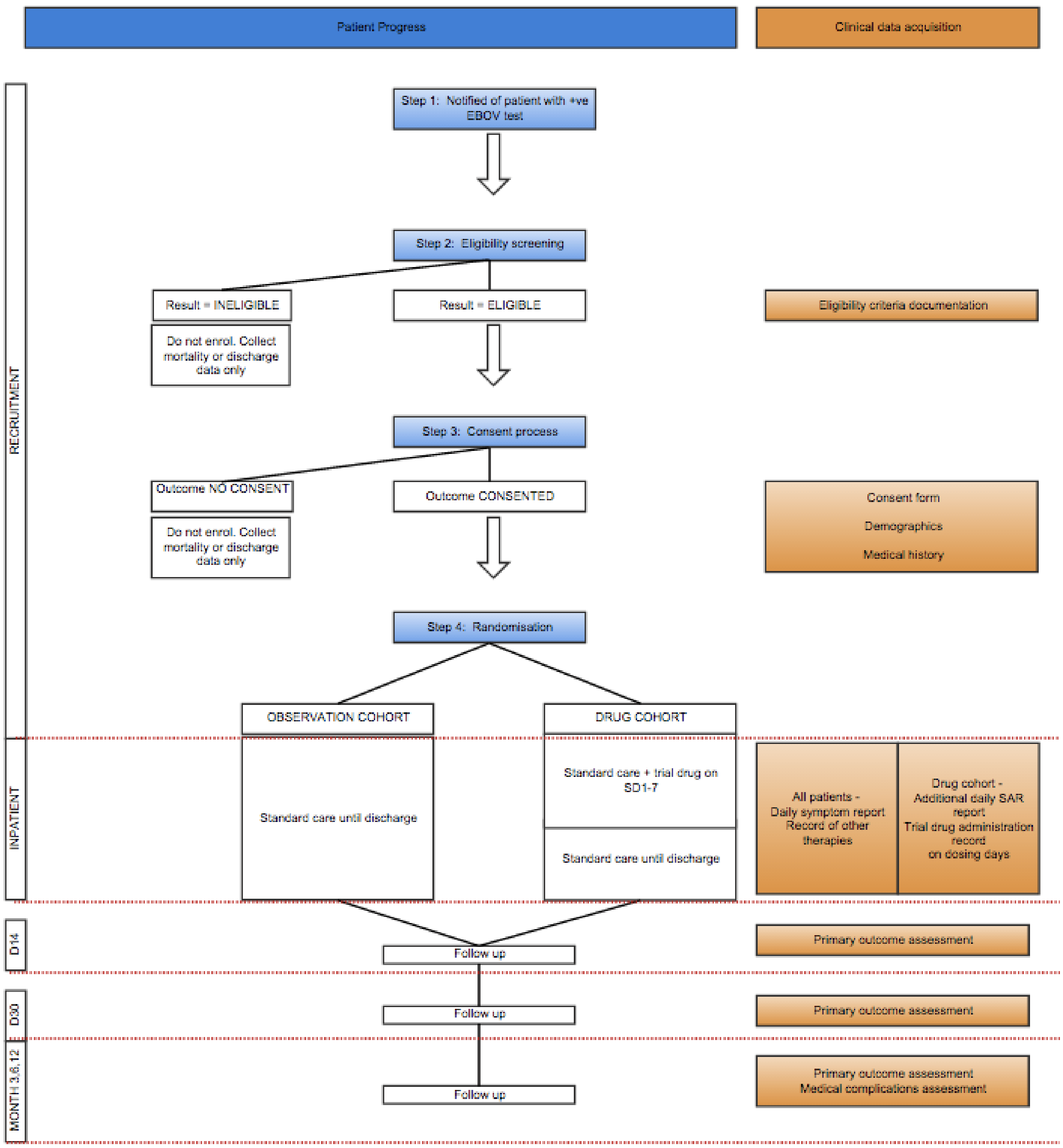

9.2. Laboratory Assessments

The timing of laboratory sampling for research purposes is outlined in Figure 4. The 'recruitment' time point for laboratory samples is defined as after consent is signed and screening completed but before first dose. This could be 1 calendar day before first dose, or be same day (SD1).

The only essential blood tests are the diagnostic EBOV positive PCR on admission and EBOV negative PCR prior to discharge (a single, or two negative PCR results will be acceptable, depending on local ETC policy). Adult and pediatric samples will be reduced in volume according to standard procedures such that no patient will have more than 0.6 mls/kg (>1% blood volume) taken on any one day, nor more than 2.4 mls/kg (approximately 3% of blood volume) taken in any four week period. Standard care samples will be prioritised over research samples if volume reduction is required. Ability to take samples is dependent on

staff availability, the availability of suitable laboratory facilities, caseload and, particularly at discharge once patients are released to go home, the willingness of the patient. Research samples and standard care samples may be reduced where required, to maintain care standards and staff safety, and reflect the assays that can be performed by the laboratory attached to the ETC. Study samples may also be reduced if the patient refuses them or is not available. Other chemical or biological assays may be performed using the same blood volumes if available and useful to inform patient safety or study outcomes.

Figure 4: Sample scheduling for trial specimens – TKM study.

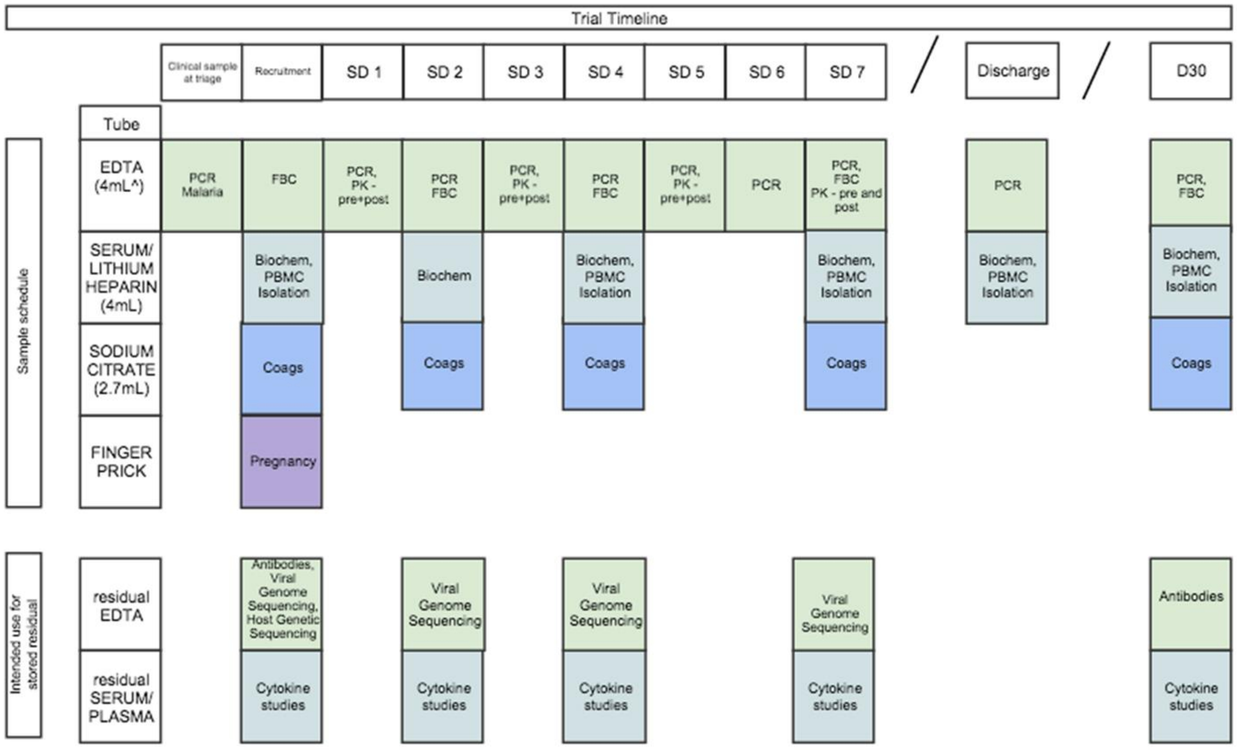

* Timing assumes that patients commence infusion on day 1, following the recruitment day. If commencement of infusion occurs at a later time, the schedule will be altered to reflect this.
^ 1 x 4 mL EDTA sample is obtained for PCR and malaria. An additional 1 x 4 mL EDTA sample will be obtained on study days when FBC is required. Patients who have PK testing undertaken will have 2 x EDTA samples drawn

Depending on laboratory capacity, it may not be possible to perform the following tests or complete assays. Where possible, tested parameters may include:

Biochemistry: Renal profile (sodium, potassium, chloride, blood urea nitrogen, creatinine), Bone profile (calcium, magnesium), liver function tests (ALT, AST, alkaline phosphatase, total bilirubin, albumin, total protein), other (C-reactive protein, total carbon dioxide, glucose, total creatinine kinase, CK-MB)

Coagulation studies: prothrombin time, activated partial thromboplastin time, and international normalised ratio

Full blood count: haemoglobin, total white cell count, platelet count, lymphocyte count, granulocyte (neutrophil) count, monocyte count, haematocrit, mean corpuscular volume

The same sampling schedule applies to all patients irrespective of their allocation in either the observational or TKM cohort, with the exception of pharmacokinetic drug testing. This will only be performed on patients receiving the trial drug.

Malaria testing: Malaria diagnostic tests will be performed on the triage blood sample as part of standard care. The results of these tests will be recorded. Trial staff will only request a malaria test at recruitment if it has not been done by the clinical treating staff at triage.

Pregnancy testing: For women of childbearing age (15-49 years) a chromatographic βHCG test will be performed on the triage blood sample. Urine testing is also acceptable.

Ebolavirus testing: Patients will have blood samples collected at triage for EVD diagnosis by PCR and prior to discharge to confirm clearance of viral RNA, both as standard care. In addition, a study only EDTA will

be taken daily for the first 7 days of study involvement (SDs 1-7) and on SD30 for PCR. The PCR result for all samples will be shared with the clinical team in real time. Samples obtained for PCR are typically ≤4 ml whole blood in an EDTA tube; finger or heal-pricks of blood on a dry swab are sometimes obtained when venepuncture is not possible. Ebolavirus RNA will be detected by PCR, as per local clinical laboratory protocols. Additionally, plasma anti-Ebolavirus antibody titres will be determined by analysis performed by partner laboratories of residual volumes obtained at recruitment and SD30.. This is to determine whether the treatment influences the subsequent production of specific antibodies.

Biochemistry: A 4ml sample will be drawn for additional testing on recruitment, SDs 2, 4 and 7, discharge and 30 to investigate electrolytes, renal, metabolic and hepatic function. Electrolyte disturbances are a common complication of Ebola. Results from toxicological studies indicate TKM may cause hepatic impairment, although this has not been shown in human trials. This information will be provided to clinical staff in real time.

Heamatology (Full blood count and differential white cell count): A 4ml EDTA sample will be drawn for additional testing on recruitment, SDs 2, 4, 7 and SD30 to measure wherever possible haemoglobin, total white cell count, platelet count, lymphocyte count, granulocyte (neutrophil) count, monocyte count, haematocrit and mean corpuscular volume (indicated as FBC in Figure 4).

Pharmacokinetic testing. Pharmacokinetic (PK) testing will only be undertaken on patients receiving the study drug. In order to assess the PK of TKM following repeat dosing, it is recommended that blood samples be obtained for PK analysis, if feasible, depending on the capabilities of the ETC, the availability of appropriate laboratory facilities and the clinical condition of the treated individual. It is recommended that PK samples (each one 4ml EDTA tube) be obtained pre-dose and again at the end of the infusion, on SDs 1, 3, 5, and 7 of treatment. The PK samples should be obtained, processed and stored according to the instructions provided separately in the laboratory manual.

Peripheral blood mononuclear cell (PBMC). If it is possible for the laboratory to perform PBMC separation and store separated PBMCs appropriately, then a proportion of EDTA blood will be used for this purpose. Separated PBMCs may be used subsequently for cytokines mRNA analysis, B cell repertoire sequencing, or any other type of genetic analysis based on the outcome of infection.

Residual volumes. Residual volumes of all samples will be stored and subsequently shipped to international partner laboratories for confirmatory testing and quantification. If volume remains, additional testing relevant to the pathogenesis of EVD or effects of the study treatment will be performed. Residual cells or buffy coat and plasma supernatant buffy coat may be retained for host genetic studies to identify susceptibility and severity markers.

10. TRIAL DRUGS

10.1. Formulation

The investigational product, TKM-130803 Injection is presented as an aqueous dispersion of nucleic acid/lipid particles (also referred to as Lipid Nanoparticles, LNP) in a 10 mL United States pharmacopeia (USP) Type I borosilicate glass vial with a Fluorotec®-faced butyl rubber stopper and an aluminum flip-off cap. TKM-130803 is a formulation of siEbola-3 drug substance with lipid excipients. The nucleic acid/lipid particles (LNPs) have an average size of approximately 60 to 90 nm.

The nominal mg/mL drug concentration of the specific batch of TKM-130803 will be recorded on each vial and also on the corresponding storage cartons. **TKM-130803 vials should remain in the cartons protected from light and stored refrigerated at 2 to 8°C** (35.6 to 46.4°F). **DO NOT FREEZE.**

TKM will be administered by a two hour IV infusion. The patient will receive 150mL of TKM-130803 dosing solution in 0.9%NaCl. An infusion pump should be used when administering TKM-130803 to ensure a well-controlled rate of infusion.

For full details of infusion preparation and administration please refer to the relevant trial Standard Operating Procedure manual.

10.2. Storage and Accountability

TKM vials should be stored refrigerated at 2 to 8°C (35.6 to 46.4°F). They must not be frozen.

Inventory, dispensing and accountability of study treatment will be tightly controlled. Treatment dispensed to the Confirmed ward, but not consumed by patients will not be returned due to infection control procedures and will be destroyed. Full accountability procedures and logs will be detailed in the corresponding study reference SOPs.

11. SAFETY REPORTING

Due to the nature of the symptoms of EVD (see **Table 2**) [2] and the relatively small sample size it will be very difficult to differentiate between symptoms of the disease and events due to treatment, and the trial will have limited power to do this. Our investigation of safety will therefore focus on Serious Adverse Reactions (SARs) and Suspected Unexpected Serious Adverse Reaction (SUSARs) and key adverse events [e.g. diarrhoea, vomiting, bleeding, allergic reaction, anaphylaxis, cytokine release, flushing, hypotension and pain (chest pain, back pain, abdominal pain and headache)]. We will collect data using the CRF on these symptoms if they are new or worsening including, as far as possible, a severity grading in line with the CTCAE (Common Terminology Criteria for Adverse Events). These will only be reported to TOG/IDMC in accordance with the safety reporting as described below.

11.1. Definitions for safety reporting

Serious Adverse Reaction (SAR) – A serious untoward and unintended response in a participant to the study treatment, which is related (or has a reasonable possibility of being related) to any dose administered to that participant.

To qualify as “serious” the response must meet one of the following criteria:

- results in death
- is life-threatening
- requires inpatient hospitalisation or prolongation of existing hospitalisation
- results in persistent or significant disability/incapacity
- consists of a congenital anomaly or birth defect.

Other ‘important medical events’ may also be considered serious if they jeopardise the participant or require an intervention to prevent one of the above consequences.

For this protocol, due to the expected high death rate due to EVD, medical events that are not considered related to any dose of study treatment are *not* considered to be Serious Adverse Events (SAEs).

Suspected Unexpected Serious Adverse Reaction (SUSAR) - A serious adverse reaction, the nature and severity of which is not consistent with the information about the medicinal product in question set out in the investigator's brochure (IB).

11.2. Procedures for safety reporting

All SARs will be reported by the site to the operational team within 48 hours. In discussion with the site staff, the operational team will assess the SAR for expectedness and relatedness and report all SUSARs to the independent data monitoring committee (IDMC) immediately. The operational team will also report any SARs and SUSARs to all the relevant parties (such as IRBs, regulatory authorities and Tekmira) as required within 7 days.

Evaluation relatedness of reactions will consider the opinion of the site staff and the expected signs, symptoms and events associated with Ebolavirus disease or trial treatment according to the relevant Investigators' Brochure. Evaluation of expectedness will be made with reference to the Investigator's brochure. Serious infusion reactions will be reported. While the clinical syndrome of cytokine release may mimic other symptoms of EBOV, if suspected and serious, it will be reported to the TOG/IDMC.

Table 2. *Symptoms of Ebolavirus disease (WHO Ebola Response Team, 2014[2]; ISARIC WHO Viral Haemorrhagic Fever CRF)*

Known symptoms and clinical events of EVD:	
Death	Coma or unconsciousness
Fever	Unexplained bleeding
Fatigue	Hematemesis
Loss of appetite	Blood in stool
Vomiting	Bleeding gums
Diarrhoea	Bloody nose
Headache	Bloody cough
Abdominal pain	Other bleeding
Joint pain	Bleeding at injection site
Muscle pain	Blood from vagina
Chest pain	Blood in urine
Cough	Bleeding under the skin/bruising
Difficulty breathing	Back pain
Difficulty swallowing	Decreased urine output
Conjunctivitis	Lower chest wall indrawing
Sore throat	Hearing impairment
Confusion	Tinnitus
Hiccups	Seizures
Jaundice	Hepatomegaly
Eye pain	Splenomegaly
Rash	Lymphadenopathy

12. STATISTICS

12.1. Description of Statistical Methods

The trial is designed to determine whether TKM is a promising treatment for Ebola. No early stopping in the case of evidence of efficacy is proposed, as there are currently only 100 courses of TKM available so early general roll out of the treatment is not possible. Enrolling the full 100 patients will maximize the precision of the final estimate of efficacy.

A futility design (Whitehead and Matsushita, 2003) is therefore used to allow early stopping in the event of evidence of futility or harm. This approach will recruit up to 100 patients, but will stop if the number of successes observed so far falls below a certain threshold.

In order to avoid early stopping due to enrollment of patients with very severe, late Ebola virus disease, who may not be expected to be salvaged even by an effective antiviral therapy, the stopping rule will be calculated after exclusion of enrolled patients who die within 48 hours of admission to the ETU.

For the purposes of determining futility, the effectiveness of the treatment will be judged in terms of the probability that a patient allocated to TKM treatment will survive to D14, after excluding patients who die within 48 hours of admission to the ETU. This probability will be denoted by p. Note that p represents the true value of the probability rather than any estimate that might be found from the trial data. The value $p = 0.55$ will be used for guidance. If $p \leq 0.55$, then the treatment will be regarded as 'not promising' and the trial will be terminated.

The choice of guide value for p has been made following an analysis of individual level data on patients from a Ebola Treatment Centres from this current outbreak at Gueckedou, Foya, Kailahun and Elwa 3. Data on the outcomes of 1592 patients with laboratory confirmed Ebolavirus infection from these four centres are shown in Table 3. Only data from adults have been included, and data on patients who died on the day of admission or on the following day, or else the day after that have been omitted.

Table 3: *Estimates and 95% confidence intervals for the probability of surviving to Day 14 following admission to the treatment centre, excluding patients who die on the date of admission or on the day after or the day after that. Success is taken to be a difference between the date of death and the date of admission that is > 13 days.*

	Gueckedou	Foya	Kailahun	Elwa 3	Overall
Lower limit	0.485	0.428	0.514	0.510	0.517
Estimate	0.535	0.495	0.568	0.554	0.543
Upper limit	0.585	0.562	0.622	0.598	0.569

As a result of this analysis, $p = 0.55$ has been set as the success rate threshold above which TKM would be considered promising.

While children are initially excluded from the TKM arm of the trial, if the eligibility criteria is expanded to age >5 following IDMC review of preliminary results, TKM trial data from adults and children will be jointly analysed, since the availability of TKM is restricted to 100 treatment courses and a stratified analysis is infeasible. Case fatality is highest in children aged < 5 years, after which it declines with age reaching a minimum in persons aged about 15-20 years. Therefore the inclusion of subjects aged 5-17 years is not expected to inflate the case fatality, and the futility threshold of $p \leq 0.55$ is expected to remain valid.

12.2. The Number of Participants

The maximum number of participants available to evaluate the drug efficacy is 100. The data will be analysed sequentially and stopping rules applied.

12.3. Criteria for the Termination of the Trial

Research sites will inform the data management centre every time that a patient is allocated to TKM treatment. Fourteen days after admission, they will report to the data management centre whether that patient did, or did not, survive to D14. Every time that a D14 report is received on a patient, a point will be plotted on Figure 5**Error! Reference source not found.**. This is plot of the number of survivals (S) reported so far against the number of D14 reports received (n), after excluding from both S and n those patients allocated to TKM treatment who died within 48 hours of admission. The plot is compared with the lower red boundary shown. If the plot reaches this boundary, the trial will be stopped with the conclusion that treatment with TKM is not promising. This safety plot will ensure a rapid reaction if TKM proves to be ineffective or harmful in the short term.

If the plot continues above the boundary until the green boundary is reached, corresponding to D14 reports being received from 100 patients, then it will be concluded that TKM is a promising treatment. If the trial has to be terminated before 100 patients have received TKM and before the lower red boundary is reached, and if stopping is due to a shortage of eligible patients rather than to any safety concerns, the final dataset will be analysed. If the outcomes of patients recruited are good enough it remains possible for TKM to be found promising, even following an incomplete study. Nevertheless, every effort will be made to complete the study of 100 patients if the lower boundary is not crossed and no important safety concerns emerge.

***Figure 5**. The stopping rule for the trial*

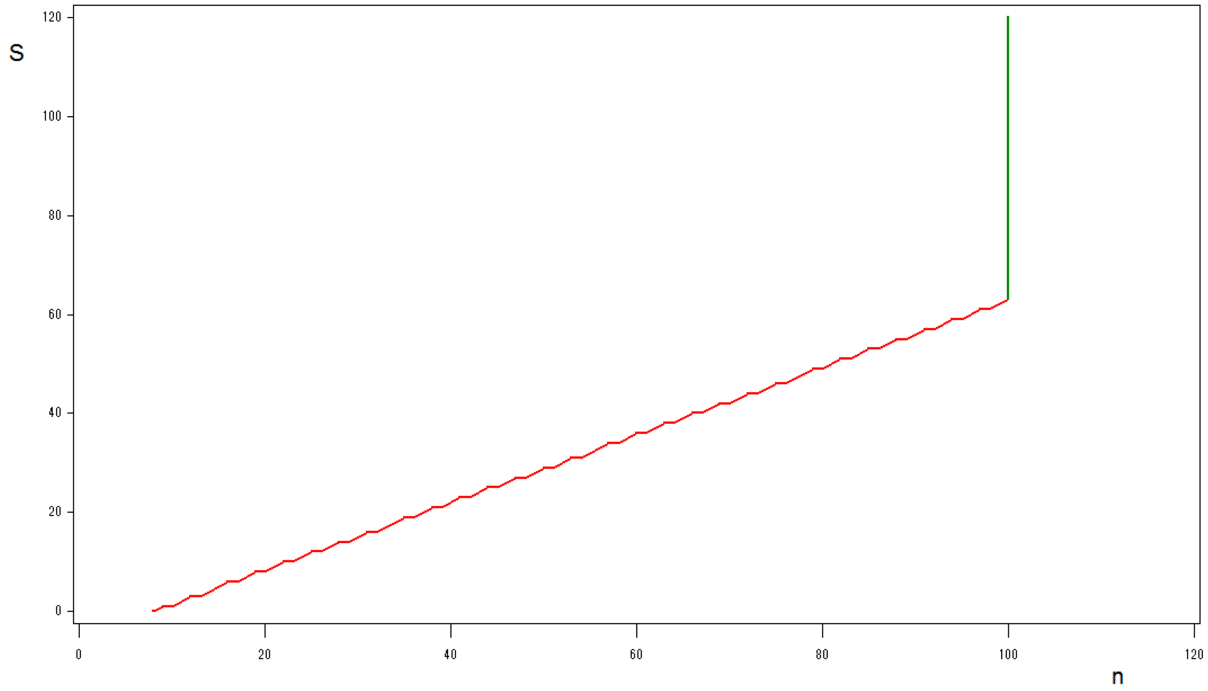

The properties of the design have been calculated exactly, based on the independent Bernoulli distributions of each patient outcome. These are shown in Table 4 and depict the specified probability of reaching the most appropriate conclusion for a range of values of p. It can be seen that the probability is declaring TKM to be promising is set at 0.025 according to this design. Recommending a treatment associated with such a low probability of survival to D14 would be considered a type I error, and the risk

of this has been set at the conventional value of 0.025. The power of correctly identifying TKM as promising when p = 0.70 is 0.827.

Table 4: Probabilities of claiming that TKM is promising for the phase II trial of Treatment TKM

True probability of success (p)	Probability of claiming that TKM is promising
0.50	0.002
0.55	0.025
0.60	0.158
0.65	0.487
0.70	0.827
0.75	0.973
0.80	0.998
0.85	1.000
0.90	1.000

12.4. Final Analysis

When the trial has been completed, a point estimate and a 95% confidence interval will be computed for p using the method of Jovic and Whitehead,[19] which allows for the sequential nature of the trial. If the trial has to be abandoned before the stopping rule is met due to lack of patients, or for any other reason that is independent of the emerging results of the trial, then an underrunning analysis will be used to compute a point estimate and a 95% confidence interval for p. This will also allow for the futility stopping rule imposed (Whitehead, 1992).

12.5. Inclusion in Analysis

It is important to note that the primary sequential analysis described uses only data from patients who were admitted more than 14 days previously. Although it may be known that some more recently recruited patients have already died, their data will not yet be used, to avoid bias due to information on death being available sooner than information on survival. Enrolled patients who die within 48 hours of admission to the ETU will be excluded from the analysis.

12.6. Procedure for Accounting for Missing, Unused, and Spurious Data.

While considerable effort will be made to ensure all patients are followed up at D14, D30, month 3, 6 and 12 (through return visits to clinic, phone, email or field-researcher visits), some patients may not be contactable. Leeway will have to be allowed for late reporting, but this should be limited: it may be that data on survival to D14 is not entered until Day 16 in order to avoid remaining bias due to late reporting of survival. However, if it turns out that a patient died on Day 15 or later, *then they are survivors* for the purpose of this study. If they walked away on Day 7, seemingly cured, it might be assumed that they survived to Day 14. Some judgment will necessarily be used here.

12.7. Rationale for statistical design choice

In the context of the current EVD outbreak, a simple, robust and rapid trial is essential. Mortality dominates all other patient endpoints. Most deaths occur within 14 days of diagnosis, and so survival (or

not) to D14 represents the most relevant clinical endpoint. The situation at Ebola Treatment Centres is highly challenging, and so this study places minimal additional responsibilities on them.

A design with a lower stopping boundary is proposed so that, if the trial is to conclude that TKM is not promising, this can be done as quickly as possible. If all treated patients die before D14, then only 8 patients will be required. No early stopping in the case of evidence of efficacy is proposed, as there are currently only 100 courses of TKM available so that no early general roll out of the treatment is possible. Rapid reporting of minimal information would appear to be feasible, and in keeping with the urgency of the situation. Until the trial is terminated, data managers need only compare the number of survivals observed so far with the stopping boundary**Error! Reference source not found.**. A table of values will be provided for this purpose.

The approach described is a form of the futility design, as described by Whitehead and Matsushita [23] The analysis is based on an orderings approach introduced by Fairbanks and Madsen.[24] Exact calculations have been made for this particular design, using SAS, and this package will be used for the analysis.

13. DATA MANAGEMENT

13.1. Data Capture

Patient files will serve as source clinical and drug dosing data for the study. Due to infection control procedures, paper documents cannot be taken outside of the high-risk containment zone; therefore records may be dictated to a secondary document outside of the zone and the primary document incinerated. Where possible, electronic data entry will capture source data directly or source documents will be photographed/scanned and archived. Original data files from the site and research laboratories will provide source data for laboratory testing results. Data recorded by clinical staff as a part of the standard case investigation form will be used to support the classification of disease severity as well as for validation and comparison of study data.

Data will be entered to a central study database on MACRO software. Data management, reporting and storage within this trial will comply with the requirements of European Union data protection laws, ICH Good Clinical Practice and FDA 21 CFR Part 11.

13.2. Access to Data

Data sharing will be under the aegis of the Trial Steering Committee and will adhere to the principles of rapid, open access as outlined in the World Health Organisation report on Ethical considerations for use of unregistered Interventions for Ebolavirus disease.[25]

14. QUALITY ASSURANCE PROCEDURES

Governance of the trial will be through the Trial Steering Committee (TSC), the Trial Operation Group (TOG) and the Independent Data Monitoring Committee (IDMC).

- The TSC will be the primary decision making body, with membership from each partner institution, a senior representative from each participating country, and other independent experts. Members of the TSC are given in Section 1 (members may change if necessary). Decisions of the Committee

will be by consensus. WHO will be part of the Committee on an ex-officio basis. Terms of reference for the TSC will set out the full details of the membership, operations and remit.

- The TOG will be led from the ISARIC Coordination Centre in Oxford and report to the TSC. This team will provide operational oversight and day-to-day management of the trial to ensure compliance to follow up and critical factors such a retention and data quality. This team will comprise those with responsibility for the operational activities and so include representation from each site, data management, trial operations, training and logistical support, laboratory sample processing and handling.
- The IDMC will be responsible for reviewing the data from the study. Membership will include individuals with statistical, clinical and trial expertise. An IDMC charter will fully set out the committee's remit, membership and full definition of their operational conduct in terms of reviewing efficacy and safety data and reporting their recommendations to the TSC.

The trial will be conducted in accordance with the current approved protocol, relevant regulations and standard operating procedures. Regular monitoring will be performed to ensure quality control. Data will be evaluated for compliance with the protocol and accuracy. Following written standard operating procedures, the monitors will verify that the clinical trial is conducted and data are generated, documented and reported in compliance with the protocol and the applicable regulatory requirements.

The MACRO software will be configured to automatically cross-validate across entire patient records according to the specific requirements. Data Clarification requests will be built in to the system. The data validation and quality processes will comply with the new FDA and EU recommendations for data level trial monitoring and therefore allow for data quality to be assured in real time as a mechanism for onsite monitoring.

Investigators and clinical staff treating centre are required to provide direct access to source data/documents for trial-related monitoring, audits, ethics committee review and regulatory inspection. All trial related and source documents must be kept for at least five years after the end of the trial.

15. ETHICAL AND REGULATORY CONSIDERATIONS

15.1. Regulations, Guidelines and Approval

The Investigators will ensure that this study is conducted in compliance with the current revision of the Declaration of Helsinki and the applicable principles of International Committee on Harmonization Good Clinical Practice Guidelines. Regulatory approval to conduct the study and import investigational products will be obtained from national authorities. The protocol, informed consent form and investigators brochure will be submitted to the Sierra Leone Ethics and Scientific Review Committee , the Oxford Tropical Research Ethics Committee, and where necessary, the Ebola Treatment Centre funding organisations ethics board, for written approval.

The Chief Investigator will submit and, where necessary, obtain approval from the above parties for all substantial amendments to the original approved documents.

15.2. Considerations in Informed Consent

STANDARD CARE UPON PRESENTATION: Patients are triaged to Suspected and Probable wards upon presentation based on symptom and contact history. Blood is drawn for Ebolavirus testing according to standard care at the site. Results are reported back to the clinical staff, who then visit patients to discuss the results. When laboratory results positive for Ebolavirus are received from the laboratory, a senior member of medical staff and a psycho-social worker visit the patient to discuss the results. Clinical and social counseling are provided as the patient is moved to the Confirmed ward. Recruitment to the study will not interfere with the standard care process. Research staff will be available to support ETC workers for study related tasks and to ensure that standard care is not disrupted by study procedures.

PROCESS FOR TAKING INFORMED CONSENT: Written informed consent to participate in the observational and randomise cohorts will be required from all participants or their representative. The staff who undertake the discussion of test results will decide when the patient should be approached regarding the study. This discussion should occur as soon as possible after the availability of confirmed results, but at a time when the patient is emotionally stable and the staff feel that they are capable of considering the risks and benefits of participation. A patient may be consented to take part in the study anytime within 48 hours of first arriving at the ETC with a confirmed diagnosis, or, within 48 hours of being informed of Ebolavirus positive PCR result, if this occurs while already at the centre.

All eligible patients (section 7) should be offered the opportunity to participate in the study. When the clinical staff and/or social workers agree that an adult patient is mentally and physically fit to discuss and consider participation in the study, the patient or the parent/guardian of a patient under 18 years, will be approached. If an eligible patient has reduced consciousness or is unresponsive to discussion due to illness severity, an appropriate representative will be approached for consent if available. Appropriate representatives will be selected according to the standard Ebola Treatment Centre processes for determining who can make decisions on a patient's behalf. In the case of a child who has no parent/guardian or in the case of a patient who is unfit to consent and there are no appropriate representatives available proxy consent will be sought from an independent adult, or group of adults (two or three) who have agreed to act together to give proxy consent. This person/ group member could be a doctor who is not a member of the research team, or other suitable adult willing to act in this role, who has already agreed to undertake this role and has been fully briefed on the trial.

Consent will be requested by the local study staff or another suitable qualified and trained person authorised to do so by the Principal Investigator.

ADULTS: Adult patients for the purpose of consent are defined as ≥18 years of age. Adults who are deemed fit to discuss and consider participation by the medical and social staff will give consent independently. If an adult is not considered mentally and physically fit to give informed consent, consent will be sought from a close relative who attends the clinic or an appointed representative. When consent is obtained from a relative, the patient will be approached for consent if at any time during study participation their condition improves such that they can consider consent.

CHILDREN: Children are patients less than 18 years of age. Consent for children to participate in the study will be sought from the parents or guardians of the child or an appointed representative in the case of a child without a contactable parent/guardian. Study participation will be discussed at the discretion of the clinical and social staff with children of suitable physical and mental fitness as well as emotional maturity. When consent is obtained from a representative, a parent/guardian will be approached for consent if at any time during study participation the study staff are able to contact them.

DISCUSSING CONSENT: Participants or their representative will be presented with an informed consent form that explains the purpose, methods, risks and benefits of the study. The form will be available in relevant written local languages. If the patient or representative cannot read, the form will be read to them in the presence of a witness when available. If a consent form is not available in a language familiar to the participant, staff will verbally translate the form into the language of the patient. When possible, a second member of staff will be present during the informed consent discussion to ensure that translations are appropriate, the form is read accurately, that all of the patient's questions have been addressed and to act as witness when possible. This will be facilitated by the paired working system at the treatment centres, but may not always be possible due to human resource limitations and limited shift times.

DOCUMENTING CONSENT: Patients or their representative will be asked to sign and date an informed consent form prior to participation. Those unable to write will be asked to make a mark on the signature line only. If a witness was present during the consent process s/he will co-sign the consent form to confirm the accurate reading of the form and the consent of the participant/ parent/ guardian/ representative. If consent for an adult patient is given by a representative, patients will be asked to sign a separate informed consent form when/if they are deemed physically and mentally fit at a later point during the study. When a second member of staff is present for the informed consent discussion, s/he will sign the informed consent form. A child's assent to participate will not be recorded.

ARCHIVING OF INFORMED CONSENT FORMS: Due to infection control procedures, paper cannot be transferred externally from the confirmed wards. Therefore informed consent forms will be photographed and archived electronically, then destroyed according to waste disposal procedures for contaminated substances. Patients will be given either a printed copy of the original signed form if possible or an unsigned copy to retain upon discharge.

15.3. Participant Confidentiality

The trial staff will ensure that the participants' anonymity is maintained. All study documents and samples will be labeled with an anonymous study code. Identifying information collected as a part of this study will remain confidential. For the purpose of ensuring data integrity and to facilitate quality assurance, study records will link to clinic files, which include patient's name and other identifying information. Participants' names will be recorded at the time of enrolment to allow for their identification at follow-up visits. However identifiable information will be linked to stored data or samples only by a protected Master List. This list will not be shared outside of the site study staff and no identifying information will be transferred between sites. All CRFs and samples will be labeled with a study identification number only and stored in suitable secure locations. Only persons who have signed the locally appropriate data protection training will have access to the password-protected computer where entered data is stored. After conclusion of the project, data will be removed from the computers and stored in a safe place. Any scientific publications or reports will not identify any patient by name or initials. When the research team reviews their notes, they are also bound by professional confidentiality. All study data will be stored in secure databases only accessible to study staff. Study sponsors and health authorities will be given controlled access for the purpose of audit when necessary.

15.4. Sample Confidentiality and Management

Aliquots of blood will be stored initially at the site clinical diagnostic laboratory. Subsequently, aliquots will be shipped to national and/or international partner laboratories according to national and international

regulations. Samples will be anonymised with a unique study identifier prior to shipping and any patient identifiable data will have been removed. The Country Principal Investigator and Chief Investigator will have access to the enrolment log linking study identifiers with patient identifiers. Material transfer agreements and export/import licenses (where required) will be obtained, and international regulations on storage and shipping of hazardous samples will be followed. Sample custody will be maintained by the investigators and decisions regarding use and transfer of samples will be made by the TSC. Research samples will be stored indefinitely and approval from the sponsor and/or ethics committees, as appropriate, will be sought prior to destruction. The study investigators are responsible for biological deactivation and ensuring appropriate destruction of any residual materials.

15.5. Risks and Benefits

There are no approved treatments for EVD and the standard of care remains supportive care and treatment of complications. An analysis of data on 3343 confirmed and 667 probable EVD cases estimates a case fatality rate of 70.8% (95% confidence interval [CI], 69 to 73).[2] Unpublished data from MSF indicates that the survival rate in laboratory confirmed cased of Ebolavirus infection admitted to Gueckedou Ebola Treatment Centre is not likely to be more than 50% overall (see statistics section).

The potential efficacy, compared with safety and tolerability of the trial drug, is outlined in section 5.2. Potential AEs that require increased clinical surveillance are outlined in 8.2.

Of note, the potential teratogenicity of TKM is unknown. To date, the fatality in foetus' of pregnant women with EVD is not known to be less than 100%. Pregnant women will not be included in the TKM trial initially. This will be reviewed by the TSC following enrolment and treatment of the first 15 patients.

TKM has never been used in children. In the initial stage of the study eligibility to the TKM arm will be restricted to patients 18 years of age or older until the first 15 patients are enrolled and treated. At that time all available, relevant safety data will be reviewed by the IDMC and a recommendation may be made to extend the eligibility to patients 5 years or older or to some other intermediate age between 5 to 18 years, as recommended by the IDMC.

Potential provision to younger patients, children under the age of 5, will be discussed with an expert panel.

The risks of conducting this trial include the possibility that the operation of the Ebola Treatment Centre (ETC) where the trial is conducted is compromised by any of the following events: a.) the ETC is overwhelmed by suspected EVD cases referred or self-referring in the belief that an effective treatment is available at the ETC; b.) the ETC security is breached by persons seeking access to the investigation product; c.) the security and safety of the ETC and ETC staff are compromised if there is a perception that the investigational product or the clinical trial are harmful to individual or community interests.

Whilst these are significant risks they are not in themselves arguments for not conducting this trial, since these risks will exist for all ETCs operating in this setting, the risks may be most acute for the earliest trials conducted.

Mitigation of these risks will be achieved by the following activities: A) Community sensitization and participation in the run up to trial initiation. Experienced health promotion and community outreach workers will conduct this activity. B) Study drug will be stored in a locked metal cabinet within a secure building. C) Contingency plans for handling an increase in the number of suspected cases will be developed, including strict criteria for closing to new admissions at the site conducting the study and the

identification of satellite ETC centers to accept patients that cannot be acceptable at the ETC conducting the trial. D) Evacuation plans will be in place in the event of a major security breach.

15.6. Expenses

Clinic and treatment costs are covered by the ETC funding organisation (and partner laboratories) for all patients presenting to the Ebola Treatment Centre regardless of study participation. The study drug will be supplied without cost to the patient. Patients will be issued a mobile phone for the purposes of follow-up. This phone and SIM card will become the property of the patient. Reasonable travel expenses and compensation for loss of earnings will be paid for attending follow-up at the Ebola Treatment Centre. No other funding or incentives for participation will be given to the patients.

15.7. Contemporaneous Studies

Patients cannot enroll to any other clinical trial that involves a therapeutic or care intervention while they are participating in this study. If participating sites are undertaking observational studies, patients may be co-enrolled provided the combined study procedures are considered safe and appropriate for the patients and there is no conflict of outcomes or endpoints between the studies.

15.8. Alternatives to Study Participation

All patients will be treated with the best available care regardless of study participation. Patients are free to decline participation in this study without effect on the standard care provided.

15.9. Community Engagement

Standard interventions likely to be used by the managing organisation include dissemination of health promotion messages to health authorities and communities regarding ETC activities and information on EVD transmission, prevention, reporting and response. Information on this research study would be added to these messages.

Messages to the community in EVD regions are disseminated by networks of Health Promotion workers who engage with local leaders that serve as primary sources of information for the communities. Radio broadcasting and printed information sheets and posters are also common tools. This standard protocol on dissemination of critical messages would be used to inform the communities about research done at the Ebola Treatment Centre. Information regarding the nature and purpose of the research would be distributed with a variety of tools across these networks.

Education on the current development status of EVD treatments, clinical research studies ongoing in West Africa and the scientific background and methods of the current project would be distributed to and discussed with the health authorities already engaged as a part of the Health Promotion activities.

Most treatment centres also provide psychological and social support to EVD patients. When presenting to the Ebola Treatment Centre, patients and families will be approached by a team social worker to facilitate the psychological process throughout admission, EVD confirmation, treatment and when required, death and bereavement. Patients who are discharged from the Treatment Centre will be supported in their return to the community. This team will be engaged in the research study according to

the requirements of the sites to assist with explaining the nature and methods of the research, obtain informed consent, and discuss the study with the family.

16. FINANCE AND INSURANCE

16.1. Funding

The trial is funded by the Wellcome Trust.

16.2. Insurance

The University of Oxford has arranged appropriate insurances to provide for the University's responsibilities, as Sponsor, to research subjects; and, to cover the legal liabilities of the University to those engaged by the University in the performance of this research. The University will also arrange, or arrange in conjunction with other participating partners those emergency medical repatriation facilities which can be achieved, subject to the exigencies of arranging such at the material time.

17. PUBLICATION POLICY

Results from the trial will be published in open access and the data will be available for sharing.

18. REFERENCES

1. WHO. Ebola Response Roadmap Situation Report 1. 29 August 2014. http://www.who.int/csr/disease/ebola/situation-reports/29-august-2014.pdf?ua=1. Accessed 3rd September 2014.
2. WHO Ebola Response Team. Ebola virus disease in West Africa--the first 9 months of the epidemic and forward projections. *N Engl J Med.* 2014;371(16):1481-1495.
3. Kucharski AJ, Edmunds WJ. Case fatality rate for Ebola virus disease in west Africa. *Lancet.* 2014;384(9950):1260.
4. Gire SK, Goba A, Andersen KG, et al. Genomic surveillance elucidates Ebola virus origin and transmission during the 2014 outbreak. *Science.* 2014;345(6202):1369-1372.
5. Fowler RA, Fletcher T, Fischer WA, 2nd, et al. Caring for critically ill patients with ebola virus disease. Perspectives from west Africa. *Am J Respir Crit Care Med.* 2014;190(7):733-737.
6. Mahanty S, Bray M. Pathogenesis of filoviral haemorrhagic fevers. *Lancet Infect Dis.* 2004;4(8):487-498.
7. Takada A, Kawaoka Y. The pathogenesis of Ebola hemorrhagic fever. *Trends Microbiol.* 2001;9(10):506-511.
8. Bausch DG, Towner JS, Dowell SF, et al. Assessment of the risk of Ebola virus transmission from bodily fluids and fomites. *J Infect Dis.* 2007;196 Suppl 2:S142-147.
9. Towner JS, Rollin PE, Bausch DG, et al. Rapid diagnosis of Ebola hemorrhagic fever by reverse transcription-PCR in an outbreak setting and assessment of patient viral load as a predictor of outcome. *J Virol.* 2004;78(8):4330-4341.
10. Ebihara H, Rockx B, Marzi A, et al. Host response dynamics following lethal infection of rhesus macaques with Zaire ebolavirus. *J Infect Dis.* 2011;204 Suppl 3:S991-999.
11. Rollin PE, Bausch DG, Sanchez A. Blood chemistry measurements and D-Dimer levels associated with fatal and nonfatal outcomes in humans infected with Sudan Ebola virus. *J Infect Dis.* 2007;196 Suppl 2:S364-371.
12. Wauquier N, Becquart P, Padilla C, et al. Human fatal zaire ebola virus infection is associated with an aberrant innate immunity and with massive lymphocyte apoptosis. *PLoS Negl Trop Dis.* 2010;4(10).
13. Oestereich L, Ludtke A, Wurr S, et al. Successful treatment of advanced Ebola virus infection with T-705 (favipiravir) in a small animal model. *Antiviral Res.* 2014;105:17-21.
14. Qiu X, Wong G, Audet J, et al. Reversion of advanced Ebola virus disease in nonhuman primates with ZMapp. *Nature.* 2014.
15. Warren TK, Wells J, Panchal RG, et al. Protection against filovirus diseases by a novel broad-spectrum nucleoside analogue BCX4430. *Nature.* 2014;508(7496):402-405.
16. Sayburn A. WHO gives go ahead for experimental treatments to be used in Ebola outbreak. *BMJ.* 2014;349:g5161.
17. Geisbert TW, Lee AC, Robbins M, et al. Postexposure protection of non-human primates against a lethal Ebola virus challenge with RNA interference: a proof-of-concept study. *Lancet.* 2010;375(9729):1896-1905.
18. Geisbert TW, Hensley LE, Kagan E, et al. Postexposure protection of guinea pigs against a lethal ebola virus challenge is conferred by RNA interference. *J Infect Dis.* 2006;193(12):1650-1657.
19. Jovic G, Whitehead J. An exact method for analysis following a two-stage phase II cancer clinical trial. *Stat Med.* 2010;29(30):3118-3125.
20. Clopper CJ, Pearson ES. The use of confidence or fiducial limits illustrated in the case of the binomial. *Biometrika.* 1934;26:404-413.
21. Bross IJ, Delaney MM. Significance tests for certain 2 x 2 tables. *Am J Hyg.* 1952;55(3):357-362.
22. Whitehead J. *The Design and Analysis of Sequential Clinical Trials*. Revised 2nd edition ed. Chichester: Wiley; 1997.

23. Whitehead J, Todd S. The double triangular test in practice. *Pharmaceutical Statistics.* 2004;3(1):39-49.

24. Fairbanks K, Madsen R. P values for tests using a repeated significance test design. *Biometrika.* 1982;69:69-74.

25. WHO. *Ethical considerations for use of unregistered interventions for Ebola viral disease. Report of an advisory panel to WHO.* [Online]. 2014. Available from: http://apps.who.int/iris/bitstream/10665/130997/1/WHO_HIS_KER_GHE_14.1_eng.pdf?ua=1. [Accessed 9th September 2014].

26 Whitehead, J. Overrunning and underrunning in sequential clinical trials. Controlled Clinical Trials 13, 106-121 (1992).

REPIDE TKM PROTOCOL AMENDMENT HISTORY

Protocol version	Date issued	Approved by	Author(s) of changes	Details of Changes made
**V2.7**	**18FEB2015**	Oxford Tropical Research Ethics Committee (OxTREC) - approval day 20FEB2015<br><br>Sierra Leone Ethics and Scientific Research Committee (SLESRC) - approval day 24FEB2015	Chief Investigator and Co-Investigators<br><br>Takes into account Tekmira suggestions	**Protocol v2.7 18FEB2015** was the protocol approved at the start of the trial on the 11 March 2015
**v2.8**	**19MAR2015**	OXTREC – approval day 23MAR2015<br><br>SLESRC – approval day 07APR2015	Chief Investigator and Co-Investigators	**Protocol v2.7 to v2.8 summary of changes:**<br>• In Section 7.4 there is a new paragraph (ii) to clarify the timing for consent and randomisation:<br>• Figure 2 has been amended to reflect this eligibility criteria<br>• Section 8.1 a paragraph was included to clarify the timing of consent and randomisation<br>• In section 7.5, eligibility criteria has been added:<br>• Patient ages <18 years will be excluded from both the TKM treatment and observational cohorts until subsequent time as the IDMC recommended otherwise.<br>• The eligibility list in section 7.5 and Figure 2 have been amended to reflect the above change.<br>• A new member of the Trial Steering Committee has been added, this is Fiona Gannon Quality Programme Quality Advisor from GOAL International.<br>• The list of abbreviations has been updated to clarify the two counting system used throughout the study: “D” to refer to days since admission; and “SD” for study days. All throughout the protocol the abbreviations D or SD has been included accordingly.

				• In Section 6, a note has been added to clarify the two counting systems D or SD used in the trial.<br>• Section 7.3 Eligibility for TKM, the role of the Trial Steering Committee (TSC) has been clarified to be consistent with Sections 14 and 15.5<br>• In Section 8.2 Procedure for provision of TKM, this paragraph has been amended to agree with other sections in the protocol relating to the safety cohort.<br>• Section 9 (9.1, 9.2 and 9.3) has been revised to include the D, SD abbreviations, including an update in Figure 4.<br>• Section 12.1 table 3 that presents the data analysis of adult patients from Ebola Treatment Centres from Gueckedou, Foya, Kailahun and Elwa 3 has been updated.<br>• Section 12.5 has been revised to be precise about inclusion in the primary sequential analysis; using only data from patients who were admitted more than 14 days previously.<br>• Section 12.4 Final Analysis, a paragraph was expanded to clarify the form of analysis to be used in the trial.
**v2.9**	**02JUN2015**	OxTREC – approval day 05JUN2015	Chief Investigator and Co-Investigators	**Protocol v2.8 to 2.9 summary of changes:**<br><br>1] In section 8.1 follow up Assessments (D14, D30, Months 3, 6, 12), a clarification of the process for these follow up assessments.<br>This process is describe in the SOP for follow up assessments. For visits at D30 and month 3, 6, and 12 information on symptoms post recovery will be collected in the CRF.<br><br>2] In section 9.2 Laboratory Assessment<br>The timing of laboratory sampling for research purposes has been clarified in Figure 4 including the collection at discharged and follow up samples in D30. This has been revised to include an explanatory paragraph for recruitment samples:<br>To clarify 'recruitment' time point for laboratory samples is defined as after consent form is signed and screening completed but before first dose. This could be 1 calendar day before first dose, or be same day (SD1).<br>Figure 4: Scheduling sample for trial specimens – TKM study, has been up dated to include samples schedule at discharged and in D30<br>For Malaria testing, a paragraph has been added to clarify that if Malaria testing is not performed at triage, this will be requested by the trial staff on the recruitment sample

				For Biochemistry Electrolyte, renal, metabolic and hepatic function testing: It has been clarified that additional testing will take place on recruitment, SDs 2, 4, and 7, discharge and D30 to measure wherever possible haemoglobin, total white cell count, platelet count, lymphocyte count, granulocyte (neutrophil) count, monocyte count, haematocrit and mean corpuscular volume. This is indicated as full blood count (FBC) in Figure 4. This requires 8mls EDTA (two tubes 4ml) for PCR and FBC.<br><br>Pharmacokinetic testing paragraph has been modified to specified that: PK samples should be obtained, processed and stored according to the instructions provided separately in the laboratory manual., entitled, "Instructions for the Collection, Processing, and Storage of Blood Samples for Pharmacokinetic (PK) Testing" – PK SOP and PK Manual.<br><br>And a new paragraph has been inserted for Peripheral blood mononuclear cell (PBMC). This specifies that:<br>If it is possible for the laboratory to perform PBMC separation and store separated PBMCs appropriately, then a proportion of EDTA blood will be used for this purpose. Separated PBMCs may be used subsequently for cytokines mRNA analysis, B cell repertoire sequencing, or any other type of genetic analysis based on the outcome of infection.<br><br>3] In Section 7.4 a new paragraph has been added to clarify that patients who are at the end of life care or who are late transfers from another facility and recovering from Ebolavirus disease are not eligible for the trial.<br><br>A new section has been included to explain the allocation process to observational cohort:<br>4. Allocation into the observational cohort will occur by the following process.<br>i. Following arrival of the trial staff each morning, the maximal number of observational study beds (b) available for patients will be determined by the ETC clinical lead and the trial clinical lead.<br>ii. Patients who are eligible to receive study drug and have been randomised to the observational cohort will be again randomly allocated a number (n) from 1 to N, where N is the number of patients eligible (using a random number allocation program code in R statistical software).

				iii. Patients will be allocated to the observational cohort by sequentially selecting patients by their number (n) from 1 to b. The remainder of patients will be put forward for standard care (when N>b).<br>iv. If after randomisation there is one or more observational beds unallocated, randomisation of other patients identified as eligible for the observational cohort will be performed (e.g. patients that are not eligible for randomization into TKM, but only ever directly eligible for the observational cohort such as pregnant women). These patients will also be allocate a number (n) from 1 to N , where N is the number of patients eligible (again using a random number allocation program code in R statistical software).<br>v. Patients will again be allocated to the observational cohort by sequentially selecting patients by their number (n) from 1 to b, until the unallocated observational beds have been filled.<br>vi. If on the calendar day of randomisation a patient allocated to the observational arm is unable to take part (e.g. because they refuse, die, or IV access cannot be obtained), the next patient in the sequence may be entered in to the observational arm. This may only occur on the calendar day of randomisation.<br><br>A new paragraph has been added to define the enrolment time point:<br>Enrolment time point in the trial is defined as after consent form is signed, screening completed and allocation to treatment or observational cohort is completed, but before first dose for treatment patients and data collection for observational patients.<br><br>Figure 2 has been amended to reflect the above eligibility criteria and stage process (with stages 1, 2, 3 and 4)<br><br>4] Change in co-investigators<br>Stephan Gunter is no longer a co-investigator as his institutions Bernard Nocht Institute for Tropical Medicine is no longer co-participant in the study.<br><br>5] The synopsis table has been up dated to reflect one clarification in the outcome measure for secondary outcomes that specifies: Anti-Ebolavirus IgG titer (recruitment and D30), and clinical assessment at D30 and months 3, 6, 12, will be performed for treatment and observational cohort patients.

				6] In Section 6 Objectives and Outcome Measures, the table has been updated to reflect a clarification in the outcome measure for secondary outcomes that specifies: Anti-Ebolavirus IgG titer (recruitment and D30), and clinical assessment at D30 and months 3, 6, 12, will be performed for treatment and observational cohort patients (to be consistence with the synopsis table and with protocol sections 8.1 and 9.2).<br><br>7] In Section 7.2 sentences for clarification of trial designed and rational have been added to specified organisation of treatment and observational cohorts.<br><br>8] In section 7.5 the eligibility and exclusion criteria in two step process has been clarified for the treatment and observational cohorts in line with sections 7.2 and 7.4<br>9] A paragraph has been also included in Section 8.1 to clarify the timing of consent and randomisation, specified that re-randomisation is not permitted for both treatment and observational cohorts<br>10] Section 8.2 In-patient days, it is a new section, this has been included to clarify the daily management of patient enrolled in the treatment or observational cohorts:<br><br>Standard safety assessments of patients will include daily monitoring of vital signs (pulse rate, blood pressure, respiratory rate, temperature), symptoms and SARs whilst they remain in the ETC. Any non-study medication received will also be recorded.<br><br>In addition, for the TKM treatment cohort, vital signs and assessment of possible infusion related reactions will be assessed at the following approximate time points: pre-infusion, during the infusion (preferably between 30-90 minutes), at the end of infusion (0 hour), and at 1, 2, 4 and 8 hours post end of infusion, as well as at additional time points if indicated by the patient's clinical condition.<br><br>The recruitment process for the safety cohort is described in Section 7.4. The IDMC will review data from the safety cohort prior to recruitment being opened to all eligible patients. The safety cohort may be expanded following advice from the IDMC.<br><br>11] Section 8.3 Follow up Assessments (D14, D30, Months 3, 6, 12) has been expanded to give clear guidance on assessments and sampling taken

				place on these days. This section was previously section 9.3, the new wording reads as follows:<br><br>Survival at D14 is the primary end point of this trial, and defined as survival until 23.59 on Day 14. Follow up assessments will be conducted on D14 and D30 and at 3, 6 and 12 months. Patients who have been discharged before these dates will be issued a mobile telephone for the purposes of follow-up. Patients will be invited to return to the treatment centre for the D14 and D30 visits and then subsequent follow up will occur by phone or at a treatment centre. When patients are not able or willing to attend a follow-up visit on D14 and D30 at the treatment centre, field workers will follow up by phone. Remaining follow up visits will be arranged at a survivor's clinic or suitable local health facility. An outreach team may attempt to visit participants who are unable to attend a clinic or cannot be reached by phone, if it is considered safe and feasible to do so. Reasonable expenses for travel and loss of earnings will be paid at these follow up visits.<br><br>Patient status on follow-up assessment days will be collected (admitted, met discharge criteria, and deceased). In addition, visits at D30 and months 3, 6, and 12 will seek information on symptoms post recovery. Trial staff will notify appropriate healthcare providers if the patient reports new symptoms that require further clinical assessment.<br><br>Primary/secondary outcome data for these visits can be collected at any point after the respective days.<br><br>12] In Section 8.2 is now Section 8.4 Procedure for provision of TKM, the fourth paragraph has been deleted as this is already mentioned in section 7.4:<br>The recruitment process for the safety cohort is described in Section 7.4. The IDMC will review data from the safety cohort prior to recruitment being opened to all eligible patients. The safety cohort may be expanded following advice from the IDMC.<br>The following sixth paragraph was also deleted as this safety assessments are already covered in section 11:<br>Standard safety assessments of patients will include monitoring of vital signs (pulse rate, blood pressure, respiratory rate, temperature), symptoms and SARs. Vital signs will be assessed at the following approximate time points: pre-infusion, during the infusion (preferably between 30-90 minutes), at the end of infusion, and at 1,

				2, 4 and 8 hours post end of infusion, as well as at additional time points if indicated by the patient's clinical condition.<br>13] Due to the new sections 8.2 and 8.3, the number of the following sections has been changed:<br>Section 8.4 is now Section 8.5 Laboratory Samples<br>Section 8.5 is now Section 8.6 Procedures of standard car<br>Section 8.6 is now Section 8.7 Treatment interruption<br>Section 8.7 is now Section 8.8 Discontinuation of trial treatment<br>Section 8.8 is now Section 8.9 Withdrawal from the study<br>14] Section 9.3 has been deleted and moved to Section 8.3 Follow up Assessments (D14, D30, Months 3, 6, 12).<br>15] Section 9.4 is now Section 9.2 Laboratory Assessments, this has been revised to include an explanatory paragraph for recruitment samples and Figure 4 up dated accordingly.<br>16] In Section 15.2 Consideration in Informed Consent, a paragraph has been included to clarify the process for taken consent within the time frame of 48hrs, this read as follows:<br>A patient may be consented to take part in the study anytime within 48 hours of first arriving at the ETC with a confirmed diagnosis, or, within 48 hours of being informed of Ebolavirus positive PCR result, if this occurs while already at the centre.<br>Within the section Discussing Consent, the following paragraph has been deleted, as this is not necessary in the present context, all translations (if necessary) are verbal from a member of staff who translate to the patient as indicated in protocol:<br>Standardization of translation will be supported by the use of recorded verbal translations with appropriate language and explanations in local terms that will be used to train study staff.<br>In line with Pharmacy Board of Sierra Leone (PBSL) guidelines section 3.1.5 Protocol Amendment, we will notified the authorities of this amendment with a telephone call and we are submitting this letter to notify all parties of these changes to the protocol for their consideration and approval, without stopping enrolment of patients into the trial.
